# Prediction of VRC01 neutralization sensitivity by HIV-1 gp160 sequence features

**DOI:** 10.1371/journal.pcbi.1006952

**Published:** 2019-04-01

**Authors:** Craig A. Magaret, David C. Benkeser, Brian D. Williamson, Bhavesh R. Borate, Lindsay N. Carpp, Ivelin S. Georgiev, Ian Setliff, Adam S. Dingens, Noah Simon, Marco Carone, Christopher Simpkins, David Montefiori, Galit Alter, Wen-Han Yu, Michal Juraska, Paul T. Edlefsen, Shelly Karuna, Nyaradzo M. Mgodi, Srilatha Edugupanti, Peter B. Gilbert

**Affiliations:** 1 Vaccine and Infectious Disease Division and Statistical Center for HIV/AIDS Research and Prevention, Fred Hutchinson Cancer Research Center, Seattle, Washington, United States of America; 2 Department of Biostatistics and Bioinformatics, Rollins School of Public Health, Emory University, Atlanta, Georgia, United States of America; 3 Department of Biostatistics, University of Washington, Seattle, Washington, United States of America; 4 Vanderbilt Vaccine Center, Vanderbilt University Medical Center, Nashville, Tennessee, United States of America; 5 Department of Pathology, Microbiology, and Immunology, Vanderbilt University Medical Center, Nashville, Tennessee, United States of America; 6 Department of Electrical Engineering and Computer Science, Vanderbilt University, Nashville, Tennessee, United States of America; 7 Program in Chemical & Physical Biology, Vanderbilt University Medical Center, Nashville, Tennessee, United States of America; 8 Division of Basic Sciences and Computational Biology Program, Fred Hutchinson Cancer Research Center, Seattle, Washington, United States of America; 9 Division of Human Biology and Epidemiology Program, Fred Hutchinson Cancer Research Center, Seattle, Washington, United States of America; 10 Molecular and Cellular Biology PhD Program, University of Washington, Seattle, Washington, United States of America; 11 Duke University School of Medicine, Duke University, Durham, North Carolina, United States of America; 12 Ragon Institute of MGH, MIT and Harvard, Cambridge, Massachusetts, United States of America; 13 University of Zimbabwe College of Health Sciences Clinical Trials Research Centre, Harare, Zimbabwe; 14 Department of Medicine, Division of Infectious Diseases, Emory University, Atlanta, Georgia, United States of America; Max Planck Institute for Informatics, GERMANY

## Abstract

The broadly neutralizing antibody (bnAb) VRC01 is being evaluated for its efficacy to prevent HIV-1 infection in the Antibody Mediated Prevention (AMP) trials. A secondary objective of AMP utilizes sieve analysis to investigate how VRC01 prevention efficacy (PE) varies with HIV-1 envelope (Env) amino acid (AA) sequence features. An exhaustive analysis that tests how PE depends on every AA feature with sufficient variation would have low statistical power. To design an adequately powered primary sieve analysis for AMP, we modeled VRC01 neutralization as a function of Env AA sequence features of 611 HIV-1 gp160 pseudoviruses from the CATNAP database, with objectives: (1) to develop models that best predict the neutralization readouts; and (2) to rank AA features by their predictive importance with classification and regression methods. The dataset was split in half, and machine learning algorithms were applied to each half, each analyzed separately using cross-validation and hold-out validation. We selected Super Learner, a nonparametric ensemble-based cross-validated learning method, for advancement to the primary sieve analysis. This method predicted the dichotomous resistance outcome of whether the IC_50_ neutralization titer of VRC01 for a given Env pseudovirus is right-censored (indicating resistance) with an average validated AUC of 0.868 across the two hold-out datasets. Quantitative log IC_50_ was predicted with an average validated R^2^ of 0.355. Features predicting neutralization sensitivity or resistance included 26 surface-accessible residues in the VRC01 and CD4 binding footprints, the length of gp120, the length of Env, the number of cysteines in gp120, the number of cysteines in Env, and 4 potential N-linked glycosylation sites; the top features will be advanced to the primary sieve analysis. This modeling framework may also inform the study of VRC01 in the treatment of HIV-infected persons.

## Author summary

The two Antibody Mediated Prevention (AMP) clinical trials are testing whether intravenous infusion of VRC01 (an antibody that can neutralize most HIV-1 viruses) can prevent HIV-1 infection. Since the outer envelope (Env) protein of HIV-1 is highly genetically diverse, the AMP trials will evaluate in an “amino acid sequence sieve analysis” whether VRC01 prevents infection differentially depending on Env amino acid features of exposing viruses. To maximize power of sieve analysis, the number of amino acid features tested should be limited to those most likely associated with whether the virus is sensitive to neutralization by VRC01. We used machine learning to analyze a database of 611 HIV-1 Envelope pseudoviruses, with data on how well VRC01 neutralizes each pseudovirus, to identify models that best predict neutralization sensitivity from the amino acid features and to identify the most predictive features. We identified models that could predict HIV-1 sensitivity (as opposed to resistance) to VRC01 very well, and found that several amino acid residues in Env locations where both VRC01 and the CD4 receptor bind were important for making correct predictions. Our modeling approach will enable a focused AMP sieve analysis and may be useful for studying the use of VRC01 in the treatment of HIV-infected persons.

## Introduction

The immense genetic and antigenic diversity of the trimeric HIV-1 envelope (Env) glycoprotein spike [precursor form = (gp160)_3_, proteolytically cleaved to (gp120/gp41)_3_], the major target of neutralizing antibodies, poses a significant problem in the development of an effective prophylactic vaccine. Broadly neutralizing monoclonal antibodies (bnAbs) isolated from individuals with chronic HIV-1 infection have demonstrated significant promise by targeting a wide spectrum of this diversity [1–5]. These bnAbs generally target conserved elements in one of five regions of gp160: the V2 variable region, the N332 region in the V3 region, the CD4 binding site (CD4bs), the gp120–gp41 interface, and the membrane proximal external region [6]. Knowledge of Env amino acid (AA) signature patterns that are associated with a neutralization phenotype of interest [7] informs our understanding of the relevant immunogenic characteristics of HIV-1 and has important implications for bnAb regimen and HIV-1 vaccine design.

The IgG1 monoclonal antibody (mAb) VRC01 neutralizes more than 80% of 600 viral strains tested *in vitro* [1, 8], and targets a region in the relatively conserved CD4bs [1, 2, 9]. Evidence from several experimental animal infection models [10–13] highlights the potential of bnAbs such as VRC01 to prevent HIV-1 infection when administered via passive immunization in pre-exposure or post-exposure prophylactic strategies [14, 15]. VRC01 has moved through four phase 1 clinical trials (VRC601 [16], VRC602 [17], A5340 [18], and HVTN 104 [19]) and is now being evaluated in the phase 2b Antibody Mediated Prevention (AMP) trials [HVTN 704/HPTN 085 (ClinicalTrials.gov identifier NCT02716675) and HVTN 703/HPTN 081 (ClinicalTrials.gov identifier NCT02568215)] [20], the first proof-of-concept efficacy trials in adults to determine whether passive administration of a bnAb can prevent HIV-1 acquisition in men who have sex with men and transgender persons, and women who are at risk of HIV infection. A detailed description of the AMP trials and the statistical considerations of their designs can be found in Gilbert et al. [20].

Following the conclusion of the AMP trials, we will conduct a series of “sieve analyses,” which investigate the extent to which intervention-mediated protection from infection varies with phenotypic (phenotypic sieve analysis) and AA sequence (genotypic sieve analysis) characteristics of the exposing viruses [21]. The phenotypic sieve analysis in the AMP trials will compare functional properties (such as sensitivity to VRC01-mediated neutralization) of the breakthrough founding viruses from infected VRC01 recipients versus infected placebo recipients; this kind of analysis was previously conducted in the VAX004 efficacy trial of a candidate preventive HIV-1 gp120 vaccine [22]. The other type of sieve analysis to be conducted in the AMP trials–AA sequence or genotypic sieve analysis–compares AA features of breakthrough founding Env sequences from infected VRC01 recipients versus infected placebo recipients, similar to what we and others have done in preventive vaccine efficacy trials for HIV-1 [23–29], malaria [30], and dengue [31]. An AA sequence sieve effect for a particular HIV-1 AA sequence feature is defined as significant variation in prevention efficacy against viruses with different levels of this feature.

A major challenge posed to AA sequence sieve analysis is the large number of Env AA sequence features that could be considered for analysis, as an exhaustive search for sieve effects would have low statistical power after multiple-testing adjustment. Therefore, “down-selection” of a set of top-ranked AA sequence features to the primary sieve analysis is important for conserving statistical power. To address this challenge in vaccine efficacy trials, our approach first conducts pre-specified primary analyses that focus on a limited subset of AA features based primarily on knowledge of the specificity of the vaccine-elicited immune responses, or aggregates AA information into distances to vaccine-insert sequences [24, 31, 32]. Following these primary analyses, we conduct pre-specified exploratory sieve analyses in which we search for sieve effects across a much more exhaustive set of features, considering the full proteomic sequence and other genomic features, with the goal of generating additional hypotheses about how prevention efficacy depends on pathogen proteomics/genomics.

For the AMP bnAb efficacy trials, our guiding criterion for including an AA sequence feature is evidence that it helps predict the sensitivity of the virus to VRC01-mediated neutralization, as measured *in vitro* by the TZM-bl assay that will be used for the phenotypic (neutralization) sieve analysis. Two specific objectives of our work are: (1, “model selection”) to develop a best model or best few models for predicting TZM-bl neutralization sensitivity to VRC01 and advance this model or these models for use in the primary AA sequence sieve analysis, where we refer to predicted outcomes from these models based on given virus AA sequences as “proteomic antibody resistance” (PAR) scores; and (2, “feature selection”) to rank AA sequence features by their importance for predicting TZM-bl neutralization sensitivity to VRC01, and select the most important features to advance to the primary AA sequence sieve analysis. In particular, for (1), the VRC01 resistance level of different viral Env sequences can be compared using PAR scores. As such, the AA sequence sieve analysis will estimate how prevention efficacy (PE) against HIV-1 acquisition varies with the defined PAR score of the virus, similar to the neutralization sieve analysis that assesses how PE varies with the measured IC_50_, IC_80_, or slope of the virus. This analysis will also allow a comparison of how well AA sequence information vs. measured neutralization information discriminates PE. For (2), for each advanced top-ranked feature, the AA sequence sieve analysis will consist of point and confidence interval estimates of PE against each HIV virus proteomic type defined by a level of the feature, as well as a statistical test of whether PE varies across the different virus types, with multiplicity-adjustment across the top-ranked features.

We addressed both objectives using data from the Compile, Analyze and Tally NAb Panels (CATNAP) database [8], which collates IC_50_ and IC_80_ neutralization values for specific mAbs versus HIV-1 Env pseudoviruses used in the assay, as well as the corresponding Env viral sequences. We used two machine learning approaches, both of which used a set of pre-defined AA sequence features to predict each of five TZM-bl neutralization outcomes: two dichotomous outcomes indicating a virus’s resistance vs. sensitivity status based on IC_50_, and three quantitative outcomes (log IC_50_, log IC_80_, and the estimated neutralization slope of the dose-response curve). A strength of our approach compared to previous approaches for predicting neutralization resistance from AA sequence features is that we provide formal statistical inferences (i.e., confidence intervals) for cross-validated parameters that quantify prediction accuracy. We also apply a recently proposed variable importance measure that is interpretable without requiring a particular parametric model to be correctly specified and describes importance relative to the population rather than relative to a fitted machine learning algorithm. The entire analysis was done on two independent splits of the available VRC01 CATNAP data to enable a simple way to evaluate replicability of the findings and to cross-check prediction accuracy. Objective (1) was achieved with the identification of models that provide PAR scores highly predictive of a resistant vs. sensitive virus. Objective (2) was achieved in that we identified 42 AA sequence features with high variable importance measure (VIM) scores, indicating that they were highly predictive of VRC01 neutralization sensitivity, and that were also significantly associated with neutralization.

## Results

**Fig 1:**
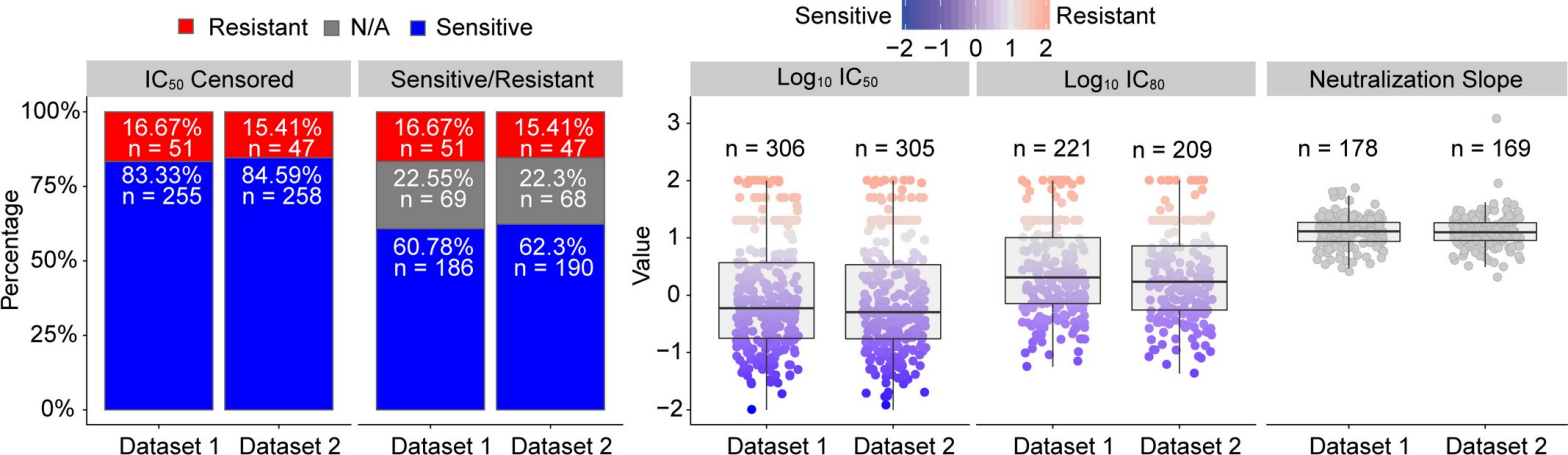
Distributions of neutralization sensitivity outcomes of Env pseudoviruses in dataset 1 and in dataset 2.

The distributions of neutralization sensitivity outcomes of Env pseudoviruses in dataset 1 (comprising half of the Env sequences retrieved from the CATNAP database, see Methods) and in dataset 2 (comprising the other half of the Env sequences retrieved from the CATNAP database, see Methods) are shown in Fig 1. Sixteen percent of all viruses included in this analysis have right-censored IC_50_ values, and hence are considered resistant for both dichotomous outcomes. For the analysis of the sensitive/resistant only outcome, 22% of all viruses were excluded from the analysis because they qualified as neither sensitive (IC_50_ < 1 μg/mL) nor resistant (right-censored IC_50_ value). In this reduced population, 79% of viruses are sensitive and 21% are resistant. The quantitative IC_50_ readouts have broader variability than the quantitative IC_80_ readouts. In all analyses, we included each virus’s geographic region of origin, to control for possible geographic confounding. All confidence intervals provided in parentheses represent 95% confidence intervals.

### Objective 1 (model selection): To develop a best model or best few models for predicting TZM-bl neutralization sensitivity to VRC01

To address objective 1, we applied nonparametric ensemble-based cross-validated learning (a form of stacking [33]) as the primary learning method, which is often referred to as super learning or the Super Learner [34] (see Methods, Statistical Learning Approaches). We compared the performance of Super Learner with each of its component learners as comparative benchmarks. The Super Learner enjoys an oracle property ensuring in large samples that its error (e.g., mean-squared error or AUC for predicting neutralization resistance from AA sequence features) is essentially the same or better than any of its individual component learners [34]. The Super Learner has also been shown to perform well in many simulation studies and in real data applications (e.g., [34–36]). We quantified model performance by rigorous inference on data-adaptive target parameters [37], including cross-validated area under the receiver operating characteristic curve (CV-AUC) for binary outcomes [38] and cross-validated nonparametric R-squared (CV-R^2^) for quantitative outcomes, the latter of which is a version of cross-validated MSE that is scaled by the variance of the outcome for the sake of interpretability [39]. Cross validation is used for an initial comparison to confirm that Super Learner performs equivalently or better than its component learners, while our primary criterion for evaluating a model’s performance is with the holdout data, using the area under the receiver operating characteristic curve (AUC) for binary outcomes and nonparametric R-squared for continuous outcomes (R^2^).

While the most auspicious models for the AMP sieve analysis will have maximally high CV-R^2^ or CV-AUC, it is difficult to define specific thresholds for these metrics for qualifying a model for use in the AMP sieve analysis. However, we propose that a bare minimal requirement is that the 95% confidence interval (CI) about the chosen cross-validated prediction accuracy metric indicates significantly greater prediction accuracy than a pure-noise model—this benchmark is a 95% CI for CV-R^2^ above 0 and a 95% CI for CV-AUC above 0.5.

To define our terminology, we use the term “prediction” to define the general case of predicting a neutralization endpoint, either dichotomous or quantitative, and we also use it in the specific case of regressing quantitative endpoints. We use the term “classification” to specify the prediction of dichotomous neutralization endpoints.

#### Classifying IC_50_-based dichotomous outcomes for VRC01 neutralization

**Fig 2:**
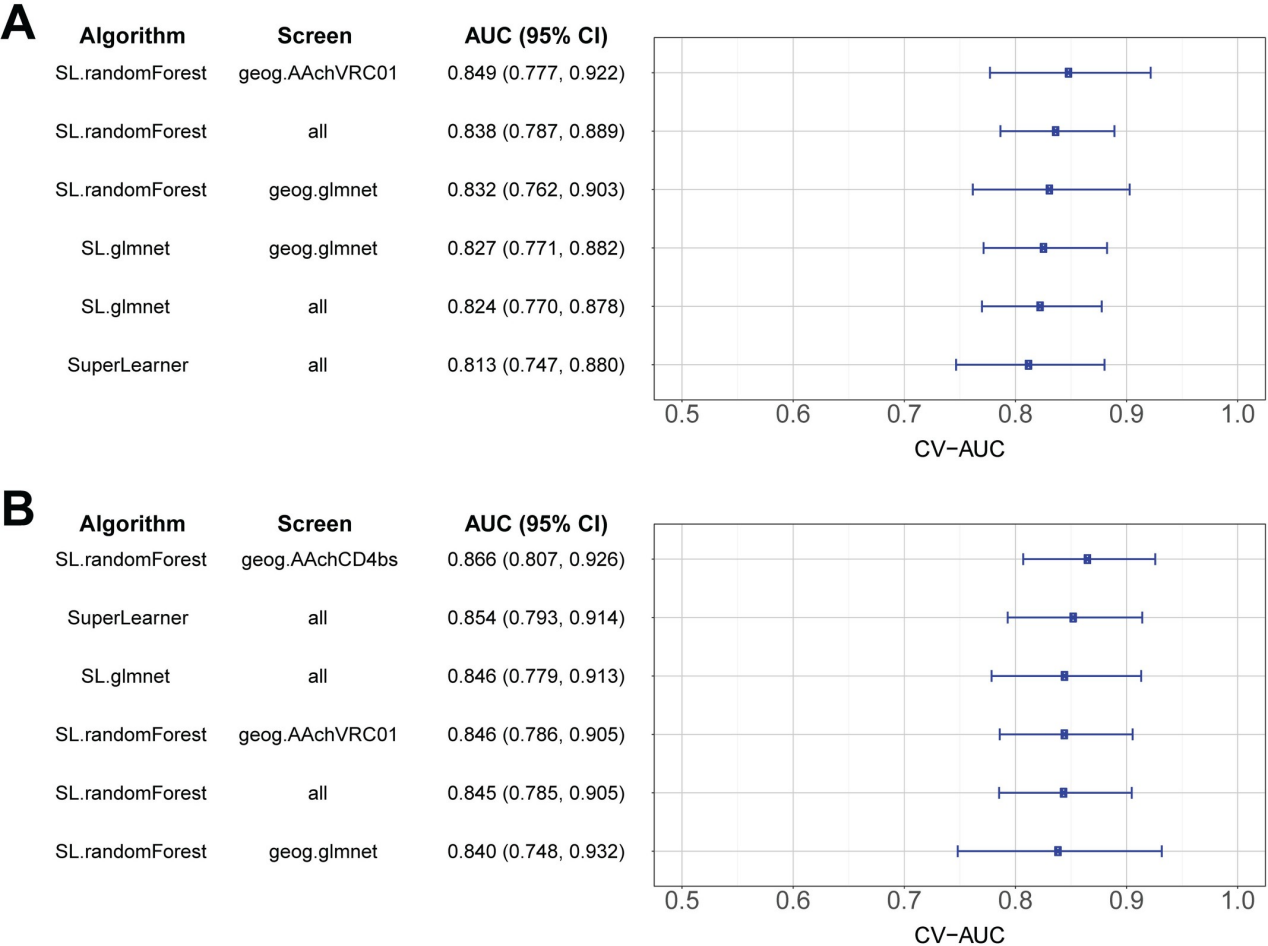
Performance of the Super Learner and the top 5 individual models in classifying the IC_50_ censored outcome. Cross-validated AUC point estimates and 95% confidence intervals are shown for A) models trained on dataset 1 and B) models trained on dataset 2.

For generating predicted probabilities of each of the two IC_50_-based dichotomous outcomes, the Super Learner and four individual models showed approximately equivalent and consistently strong performance in classifying TZM-bl neutralization sensitivity to VRC01. In dataset 1, “random forest (using geography and AAs in the VRC01 binding footprint)” (See Methods, Envelope amino acid feature input variable groups) had the best performance for the IC_50_ censored outcome, with a CV-AUC of 0.849 with 95% CI (lower bound 0.777, upper bound 0.922). In dataset 2, “random forest (using geography and AAs in the CD4 binding site)” (See Methods, Envelope amino acid feature input variable groups) had the best performance for this same outcome, with a CV-AUC of 0.866 (0.807, 0.926) (Fig 2). For this outcome, the Super Learner performed similarly to the top-performing model, and performed slightly better on dataset 2 than on dataset 1 (CV-AUC = 0.854 (0.836, 0.932) and 0.813 (0.747, 0.880), respectively).

**Fig 3:**
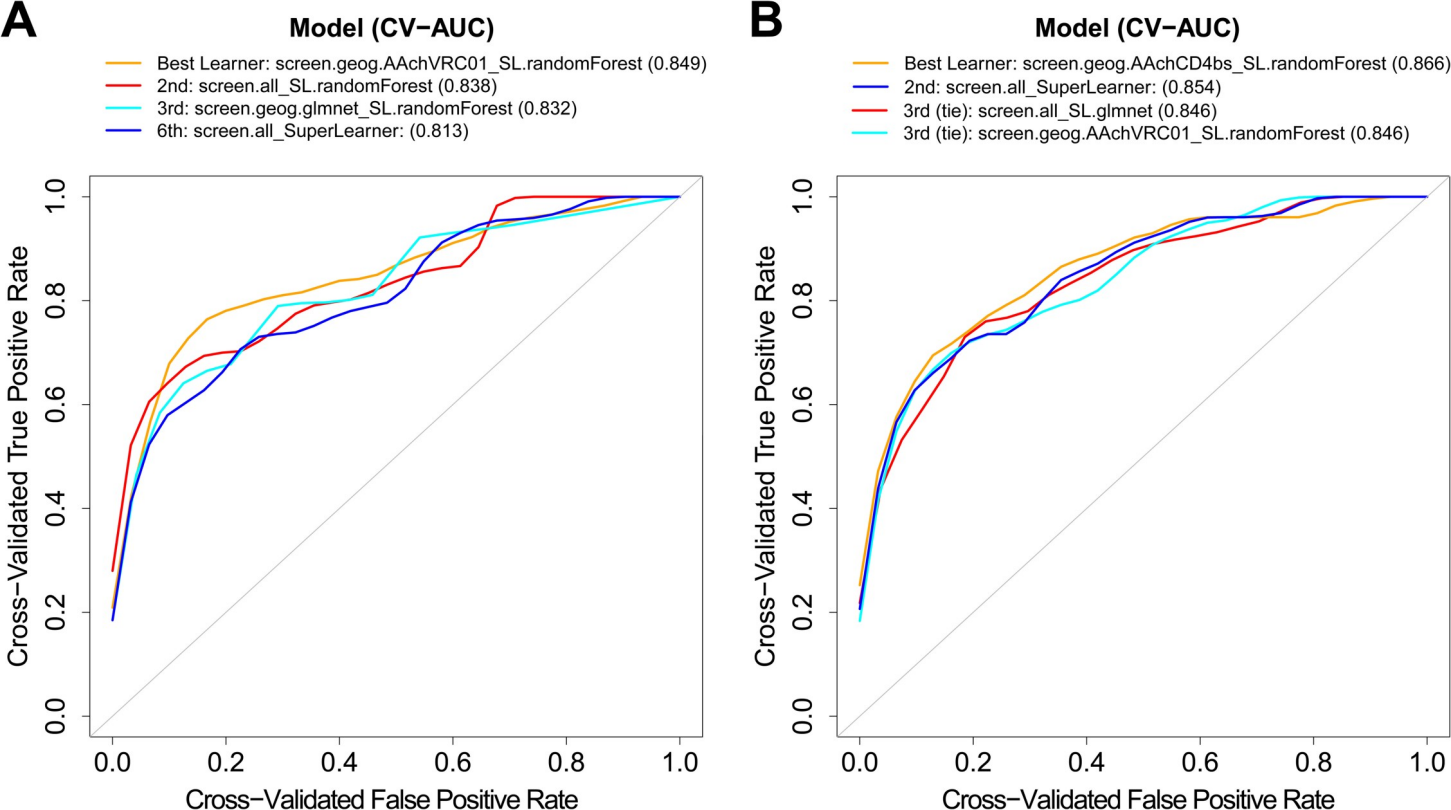
Cross-validated receiver operating characteristic curves for the best-performing models in classifying the IC_50_ censored outcome. Results are shown for the top three cross-validated models plus the cross-validated performance of the Super Learner, for A) dataset 1 and B) dataset 2. Values in parentheses are the cross-validated areas under the receiver operating characteristic curve (CV-AUC) for the different models.

**Fig 4:**
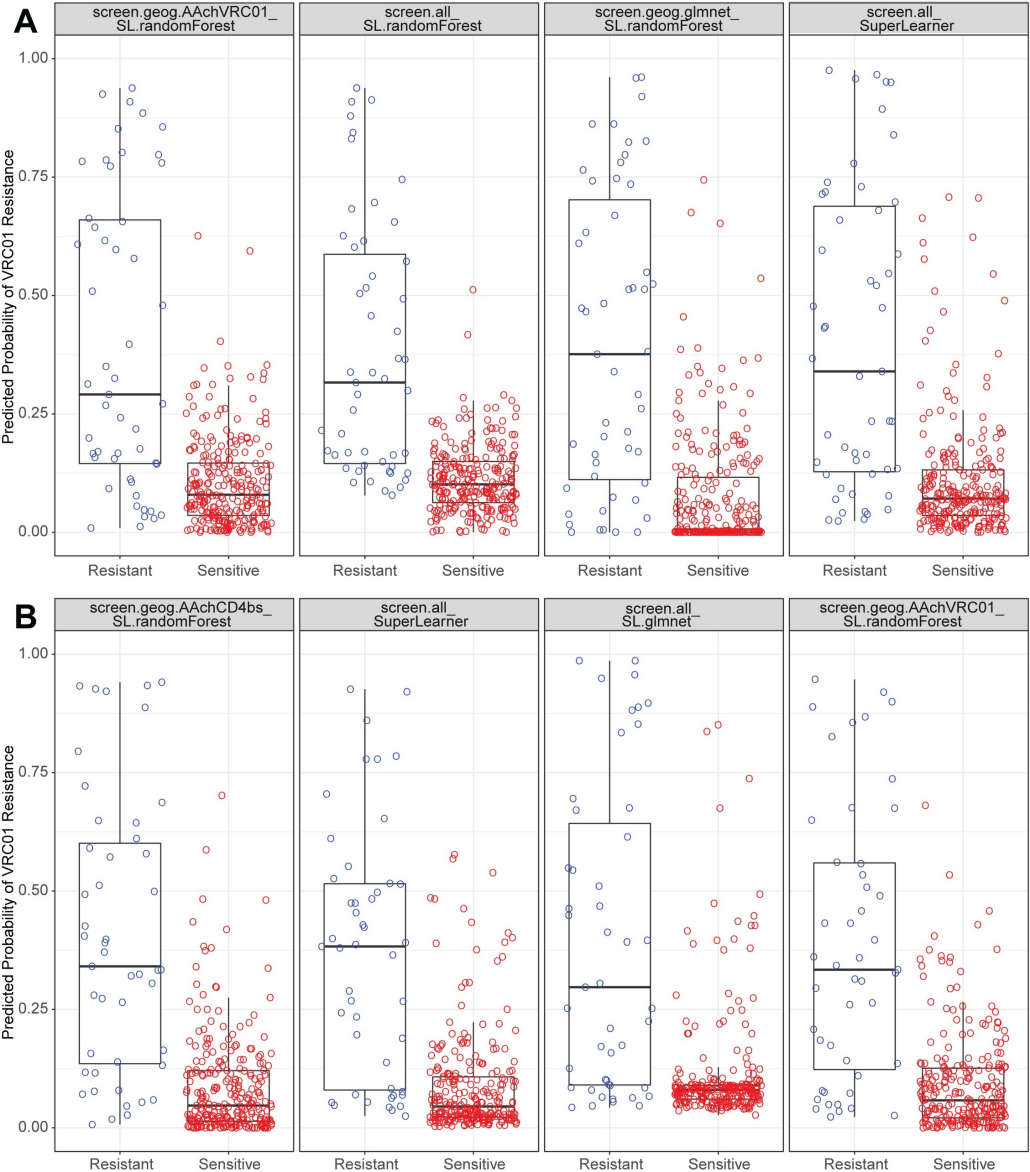
Classification boxplots for the best-performing models and the Super Learner in classifying the IC_50_ censored outcome. Cross-validated performance is shown for the Super Learner and the top three individual models for (A) dataset 1 and (B) dataset 2. Glmnet is the lasso learner.

Cross-validated ROC curves for the top four performing models are presented in Fig 3, and comparisons of these models’ performance on held-out data during cross-validation are illustrated in Fig 4. From Fig 3, we see that the models perform well enough that reasonably accurate classifications of both non-censored IC_50_ (putatively sensitive) viruses and of right-censored (resistant) viruses can be obtained, based on the choice of the ROC curve discrimination threshold, defined as a cut-point of the predicted probability of resistance. Under a standard approach that classifies a virus as right-censored/resistant if the fitted probability of right-censored exceeds 0.5, the Super Learner’s cross-validated specificity to detect a resistant virus is 0.39 on dataset 1 and 0.32 on dataset 2, compared to its cross-validated sensitivity to detect a putatively sensitive virus of 0.97 on dataset 1 and 0.99 on dataset 2. These results highlight the issue of unbalanced data, where because most viruses are VRC01-sensitive, classification accuracy is very high for sensitive viruses but low for resistant viruses. The cut-point of predicted probability for defining classified resistant vs. classified sensitive may be adjusted to be most relevant for the planned application of sieve analysis in the AMP trials. One approach that seeks to have statistical power reasonably close to optimal for a variety of possible alternative hypotheses in the sieve analysis considers the two types of misclassifications as equally costly, where, for example, setting the cut-point at predicted probability 0.12 for the Super Learner model in Fig 4 yields a misclassification rate of 25% for each category, VRC01-resistant and -sensitive, for each data set.

Similar results were seen for the sensitive/resistant only outcome (see Methods), which included a smaller subset of viruses: “random forest (using all features)” (See Methods, Envelope amino acid feature input variable groups) had the best performance in both datasets, with a CV-AUC of 0.886 (0.834, 0.938) in dataset 1 and a CV-AUC of 0.881 (0.822, 0.940) in dataset 2. Super Learner yielded the second-best performance with this endpoint, yielding a CV-AUC of 0.828 (0.764, 0.893) with dataset 1, and a CV-AUC of 0.872 (0.818, 0.925) on dataset 2. Cross-validated ROC curves for the top four performing models are presented in S1 Fig, and these models’ actual classifications are compared in S2 Fig.

Classifications of both outcomes were well-validated using the top algorithms and feature sets discovered with one dataset when evaluated using the other, completely separate, dataset. Specifically, the top models for the IC_50_ censored outcome (as determined by CV-AUC) had AUCs for the held-out dataset of 0.877 (0.819, 0.934) (trained on dataset 1, validated on dataset 2) and 0.850 (0.782, 0.918) (trained on dataset 2, validated on dataset 1), with the Super Learner performing similarly, with a hold-out AUC of 0.884 (0.836, 0.932) (trained on dataset 1, validated on dataset 2) and 0.851 (0.779, 0.922) (trained on dataset 2, validated on dataset 1) (S1 Table). Similarly, the top models for the sensitive/resistant only outcome (as determined by CV-AUC) had validated AUCs of 0.925 (0.883, 0.968) (trained on dataset 1, validated on dataset 2) and 0.904 (0.851, 0.957) (trained on dataset 2, validated on dataset 1) (S2 Table). The Super Learner was the second-best performing learner for this endpoint, with a validated AUC of 0.921 (0.882, 0.959) (trained on dataset 1, validated on dataset 2) and 0.881 (0.813, 0.948) (trained on dataset 2, validated on dataset 1).

The CV-AUC results for all models for the IC_50_ censored outcome and the sensitive/resistant only outcome, for both dataset 1 and dataset 2, are shown in S3 Fig and S4 Fig, respectively.

#### Prediction of the quantitative log IC_50_ and log IC_80_ outcomes for VRC01 neutralization

**Fig 5:**
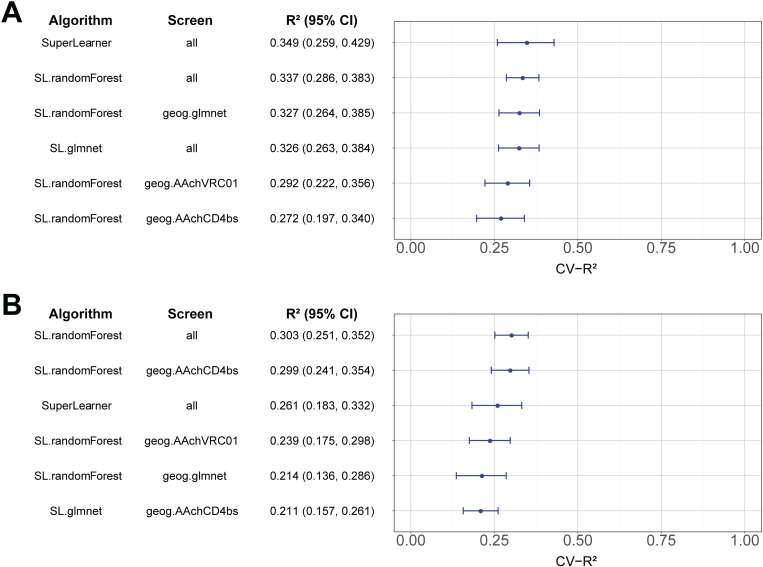
Performance of the Super Learner and the top 5 individual models in predicting the quantitative log IC_50_ outcome. Cross-validated R^2^ point estimates and 95% confidence intervals are shown for A) models trained on dataset 1 and B) models trained on dataset 2.

Of the two outcomes, the quantitative log IC_50_ outcome was predicted better than the quantitative log IC_80_ outcome. In dataset 1, the Super Learner had the best performance for the quantitative log IC_50_ outcome, with a cross-validated nonparametric R-squared (CV-R^2^) of 0.349 (0.259, 0.429). In dataset 2, “random forest (using all features)” had the best performance for this outcome, with a CV-R^2^ of 0.303 (0.251, 0.352) (Fig 5). The Super Learner was the third-best model for this dataset, with a CV-R^2^ of 0.261 (0.183, 0.332).

We saw slightly better performance when the top algorithms and feature sets chosen with one dataset were validated using the other dataset. Specifically, Super Learner was the second-best performing learner for the quantitative log IC_50_ outcome, with R^2^s for the held-out datasets of 0.331 (0.277, 0.380) (trained on set 1, validated on set 2) and 0.379 (0.327, 0.427) (trained on set 2, validated on set 1) (S3 Table). The results of the Super Learner predicting the quantitative log IC_50_ outcome for datasets 1 and 2, both cross-validated and validated with the hold-out set, are illustrated in S5 Fig.

Performance of the models for predicting the quantitative log IC_80_ outcome was notably worse. For dataset 1, the best-performing model was “lasso (using geography and AAs in the CD4 binding site)” (See Methods, Envelope amino acid feature input variable groups), with a CV-R^2^ of 0.207 (0.139, 0.270). For dataset 2, “random forest (using geography and AAs in the CD4 binding site)” was the best-performing model, with a CV-R^2^ of 0.252 (0.180, 0.317) (S4 Table). We saw slightly worse performance when algorithms were trained on one dataset and validated on the other dataset. Specifically, the top algorithms and feature sets for this outcome (as assessed by CV-R^2^) had R^2^s for the held-out datasets of 0.160 (0.088, 0.225) (trained on set 1, validated on set 2) and 0.208 (0.103, 0.301) (trained on set 2, validated on set 1) (S4 Table). The Super Learner was again the second-best performing method for predicting the quantitative log IC_80_ outcome, yielding a validated R^2^ of 0.208 (0.131, 0.278) (trained on set 1, validated on set 2) and 0.238 (0.148, 0.318) (trained on set 2, validated on set 1). The results of the Super Learner predicting the quantitative log IC_80_ outcome for datasets 1 and 2, both cross-validated and validated with the hold-out set, are illustrated in S6 Fig.

The CV-R^2^ results of all models for predicting the quantitative log IC_50_ outcome or the quantitative log IC_80_ outcome are shown in S7 Fig and S8 Fig, respectively.

#### Prediction of neutralization slope

None of the Super Learner-based approaches were able to obtain a prediction result better than a CV-R^2^ of 0.100 (0.032, 0.163) with dataset 1, or a CV-R^2^ of 0.084 (-0.002, 0.162) with dataset 2 (S5 Table). The CV-R^2^ results of all models for predicting neutralization slope are shown in S9 Fig, and the results of the Super Learner predicting the neutralization slope for datasets 1 and 2, both cross-validated and validated with the separate held-out dataset, are illustrated in S10 Fig.

### Objective 2 (feature selection): To rank amino acid sequence features by their importance for predicting TZM-bl neutralization sensitivity to VRC01

We structured the objective 2 variable importance analysis by pre-specifying thirteen distinct input variable groups of Env AA sequence features that could potentially be relevant for VRC01 neutralization based on statistical and biological (structural, immunological, and virological) grounds. The input variable groups are, with the first six based on individual Env AA positions and residues at those positions: 1) VRC01 binding footprint sites, 2) CD4 binding sites, 3) sites with sufficient exposed surface area, 4) sites identified as important for glycosylation, 5) sites with residues that covary with the VRC01 binding footprint sites, 6) sites associated with VRC01-specific potential N-linked glycosylation (PNGS) effects, 7) sites in gp41 associated with VRC01 neutralization sensitivity or resistance, 8) indication of potential N-linked glycosylation sites (PNGS), 9) majority virus subtypes, 10) region-specific counts of PNG sites, 11) viral geometry, 12) cysteine counts, and 13) steric bulk at critical locations. The Methods section provides details about the features (“Envelope amino acid feature input variable groups”).

For each of the five outcomes, we calculated VIMs for all features included in any of the 13 input variable groups with two VIM analysis approaches. The first applied a Monte Carlo Cross-Validation (MCCV)-based algorithm-specific approach that did not take into account the input variable grouping, whereas the second applied an ensemble-based approach using the Super Learner (see Methods, Statistical Learning Approaches) to assess variable importance of each of the 13 variable groups and individual features. We report as top-ranked features those with a Holm-Bonferroni adjusted 2-sided p-value < 0.05 from a univariate regression adjusted for geographic region, and that rank among the top 50 features by either of the two VIM approaches. The primary results from these VIM analyses pertain to the IC_50_ censored outcome and the log IC_50_ outcome, which are best-predicted dichotomous and quantitative outcomes with the largest sample size.

For the IC_50_ censored dichotomous outcome analysis, Table 1 reports all features that ranked among the top 50 features by either VIM method, and that had a Holm-Bonferroni 2-sided p-value less than 0.05 for an association with the outcome in a logistic regression model using both datasets (with adjustment for geographic region as in all analyses). This p-value criterion was more stringent than the pre-specified criteria, and was added to ensure that any individual features selected by our VIM method were individually predictive after strict multiplicity adjustment using a well-understood standard method, logistic regression. Table 2 shows the results for the quantitative log IC_50_ outcome, under the same rule for reporting except replacing logistic regression with linear regression. There is much overlap between the features found in Tables 1 and 2, and if we take the union of all features found for both endpoints, the result is a set of 42 unique features.

**Table 1:** Variable importance measure (VIM) information for the features that have a Holm-Bonferroni p-value less than 0.05, ranked by their contribution to the classification of the log IC_50_ censored outcome.

Feature	MCCV Composite VIM	Ensemble VIM	Ensemble VIM SE^1^	Ensemble VIM Rank	Direction of Effect^2^	p-value^3^	q-value^4^	FWER p-value^5^
456 is R	90.772	0.045	0.030	1	Sensitive	2.18E-41	1.81E-38	1.81E-38
459 is G	66.592	0.013	0.027	9	Sensitive	5.59E-33	2.32E-30	4.63E-30
280 is N	44.915	0.018	0.030	5	Sensitive	1.42E-28	3.93E-26	1.18E-25
458 is G	40.411	0.003	0.027	97	Sensitive	2.62E-28	5.43E-26	2.16E-25
655 is N	34.969	-0.004	0.027	394	Resistant	6.42E-10	1.55E-08	5.12E-07
279 is E	26.415	-0.006	0.027	553	Resistant	1.42E-05	1.61E-04	0.011
Length of gp120	23.992	-0.003	0.027	362	Resistant	2.71E-05	2.77E-04	0.02
471 is I	21.989	-0.002	0.030	278	Resistant	3.59E-14	1.19E-12	2.90E-11
181 is M	17.443	-0.009	0.027	683	Resistant	1.13E-05	1.34E-04	0.009
Length of Env	14.893	-0.002	0.027	271	Resistant	4.65E-06	5.84E-05	0.004
428 is Q	14.592	0.003	0.027	79	Sensitive	2.28E-19	1.90E-17	1.88E-16
466 is E	13.877	-0.004	0.027	426	Sensitive	4.42E-19	3.33E-17	3.62E-16
124 is P	13.515	0.005	0.028	54	Sensitive	1.04E-16	4.13E-15	8.46E-14
469 is R	13.424	-0.008	0.027	622	Sensitive	2.85E-08	5.25E-07	2.24E-05
589 is D	12.691	0.001	0.027	137	Sensitive	5.19E-15	1.87E-13	4.19E-12
Total cysteines in Env	12.57	0.003	0.027	86	Resistant	1.06E-06	1.57E-05	8.23E-04
569 is T	11.7	-0.004	0.027	395	Sensitive	5.44E-17	2.66E-15	4.43E-14
616 is PNGS	10.648	-0.002	0.027	306	Sensitive	1.38E-11	4.25E-10	1.11E-08
365 is S	10.641	-0.006	0.027	508	Sensitive	3.27E-10	8.47E-09	2.61E-07
457 is D	10.137	0	0.027	202	Sensitive	3.46E-06	4.42E-05	0.003
456 is W	10.122	-0.013	0.027	788	Resistant	4.19E-11	1.24E-09	3.36E-08
Total PNG sites in V5 region	10.083	-0.003	0.027	346	Sensitive	3.20E-05	3.05E-04	0.024
456 is H	8.955	-0.011	0.027	742	Resistant	1.42E-05	1.61E-04	0.011
374 is H	8.929	-0.008	0.026	620	Sensitive	5.87E-16	2.22E-14	4.75E-13
471 is G	8.881	0	0.027	205	Sensitive	9.08E-10	2.02E-08	7.22E-07
459 is D	7.234	0	0.027	221	Resistant	4.85E-11	1.39E-09	3.89E-08
Total cysteines in gp120	6.826	0	0.027	193	Resistant	7.53E-07	1.18E-05	5.86E-04
397 is C	6.679	0.001	0.027	133	Resistant	1.22E-24	2.03E-22	1.01E-21
425 is N	6.503	-0.012	0.027	773	Sensitive	3.45E-10	8.68E-09	2.75E-07
156 is N	6.245	-0.005	0.027	442	Sensitive	9.23E-10	2.02E-08	7.33E-07
156 is PNGS	6.222	NA	NA	805	Sensitive	9.23E-10	2.02E-08	7.33E-07
280 is S	5.592	-0.011	0.027	740	Resistant	5.76E-17	2.66E-15	4.68E-14
425 is R	5.346	-0.007	0.027	558	Resistant	1.64E-10	4.54E-09	1.31E-07
824 is PNGS	1.303	0.012	0.027	13	Resistant	3.21E-06	4.16E-05	0.002
229 is PNGS	0.99	0.013	0.028	10	Resistant	6.62E-07	1.06E-05	5.16E-04

Features shown were ranked among the top 50 features by either VIM method and had a Holm-Bonferroni 2-sided p-value less than 0.05 for an association with the outcome in a logistic regression model using both datasets (with adjustment for geographic region as in all analyses).

^1^The ensemble-based VIM standard error is based on the estimated influence function for the ensemble-based VIM [49].

^2^ When the direction of effect is “sensitive” (“resistant”), the presence of or a higher quantity of the feature associates with a censored (non-censored) IC_50_, which is interpreted as VRC01 sensitivity (resistance).

^3^ The p-value is from a Wald test in a logistic regression model testing the association of the feature with outcome, controlling for the sequences’ geographic region of origin information to control for possible confounding.

^4^ The q-value is the Benjamini-Hochberg false discovery rate.

^5^ The FWER p-value is the Holm-Bonferroni family-wise error-rate adjusted p-value.

FWER, family-wise error rate; MCCV, Monte Carlo cross-validation; SE, standard error; VIM, variable importance measure.

**Table 2:** Variable importance measure (VIM) information for the features that have a Holm-Bonferroni p-value less than 0.05, ranked by their contribution to the prediction of the quantitative log IC_50_ outcome.

Feature	MCCV Composite VIM	Ensemble VIM	Ensemble VIM SE^1^	Ensemble VIM Rank	Direction of Effect^2^	p-value^3^	q-value^4^	FWER p-value^5^
456 is R	100	0.131	0.024	15	Sensitive	1.68E-23	1.40E-20	1.40E-20
459 is G	68.458	0.11	0.022	377	Sensitive	9.00E-20	3.74E-17	7.46E-17
181 is M	40.702	0.108	0.021	504	Resistant	4.12E-06	8.77E-05	0.003
279 is D	33.329	0.109	0.021	460	Sensitive	5.39E-05	8.29E-04	0.042
Subtype is A1	31.624	0.117	0.022	56	Sensitive	1.09E-05	1.97E-04	0.009
Length of Env	30.962	0.105	0.021	680	Resistant	6.53E-06	1.29E-04	0.005
655 is N	26.362	0.103	0.021	768	Resistant	2.30E-05	3.82E-04	0.018
471 is I	23.337	0.112	0.022	204	Resistant	1.77E-09	7.74E-08	1.44E-06
Length of gp120	19.636	0.106	0.021	636	Resistant	6.86E-06	1.33E-04	0.005
471 is G	19.324	0.106	0.021	638	Sensitive	8.80E-09	3.04E-07	7.10E-06
280 is N	16.896	0.11	0.021	368	Sensitive	2.43E-15	5.05E-13	2.01E-12
179 is L	10.191	0.113	0.022	170	Sensitive	5.74E-06	1.16E-04	0.005
456 is S	6.81	0.116	0.021	69	Resistant	1.47E-06	3.48E-05	0.001
459 is D	5.934	0.106	0.021	626	Resistant	1.89E-07	5.42E-06	1.52E-04
425 is N	4.186	0.119	0.021	44	Sensitive	3.33E-09	1.38E-07	2.70E-06
455 is Q	0.011	0.12	0.022	37	Resistant	8.70E-09	3.04E-07	7.03E-06
428 is M	0.007	0.204	0.032	3	Resistant	2.31E-07	6.40E-06	1.85E-04
280 is T	0.005	0.126	0.023	19	Resistant	7.63E-06	1.44E-04	0.006

Features shown were ranked among the top 50 features by either VIM method and had a Holm-Bonferroni 2-sided p-value less than 0.05 for an association with the outcome in a linear regression model using both datasets (with adjustment for geographic region as in all analyses).

^1^The ensemble-based VIM standard error is based on the estimated influence function for the ensemble-based VIM [49].

^2^ When the direction of effect is “sensitive” (“resistant”), the presence of or a higher quantity of the feature associates with a lower (higher) quantitative log IC_50_, which is interpreted as VRC01 sensitivity (resistance).

^3^ The p-value is from a Wald test in a logistic regression model testing the association of the feature with outcome, controlling for the sequences’ geographic region of origin information to control for possible confounding.

^4^ The q-value is the Benjamini-Hochberg false discovery rate.

^5^ The FWER p-value is the Holm-Bonferroni family-wise error-rate adjusted p-value.

FWER, family-wise error rate; MCCV, Monte Carlo cross-validation; SE, standard error; VIM, variable importance measure.

**Fig 6:**
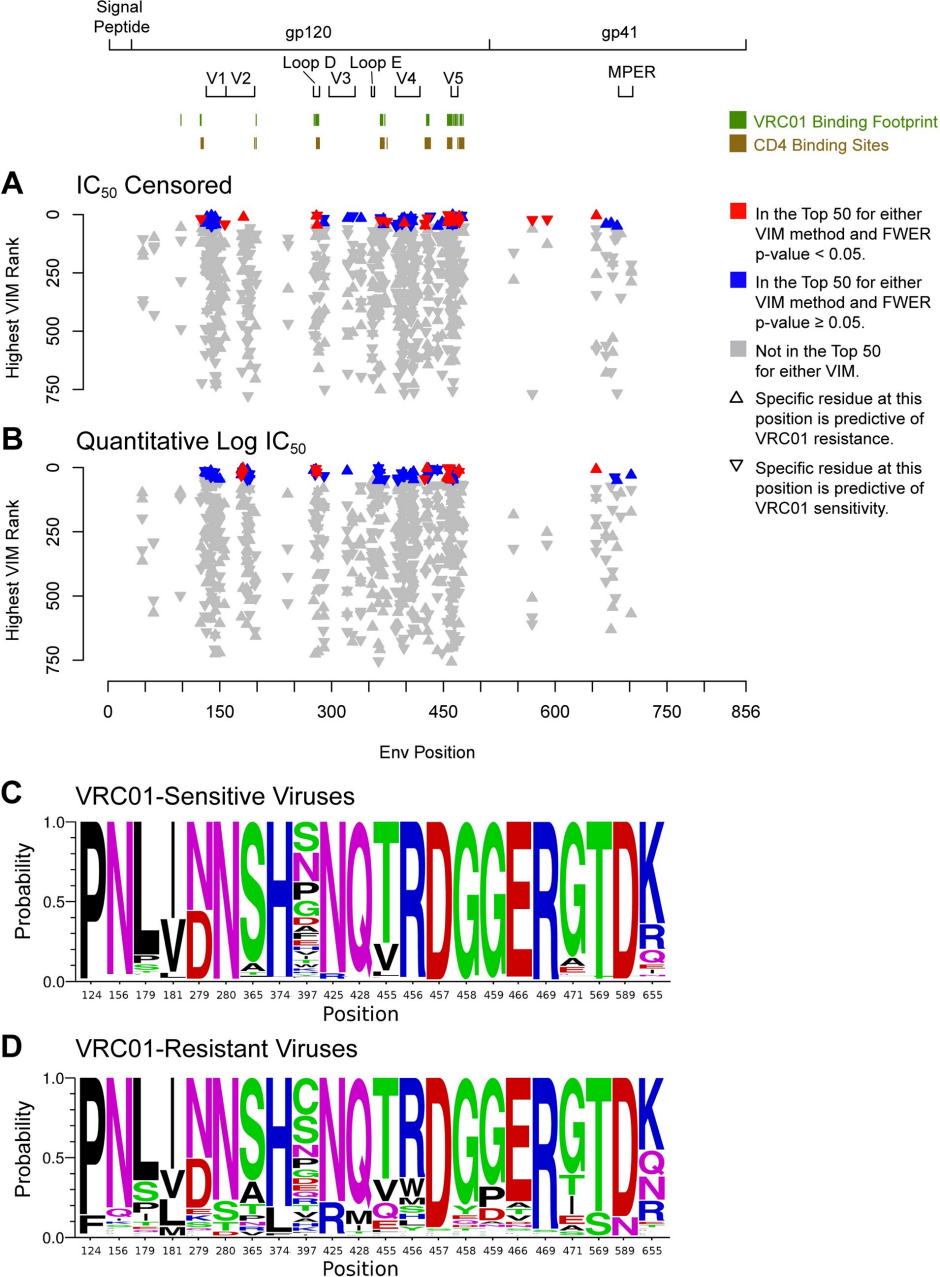
Positions, magnitudes, and distributions of amino acid residues at predictive sites selected by VIM methods. A) IC_50_ censored outcome; B) Quantitative log IC_50_ outcome. C and D) Logo plots of the probabilities of each of the amino acids observed at key positions in (C) VRC01-sensitive Env pseudoviruses and D) VRC01-resistant Env pseudoviruses. FWER, family-wise error rate; VIM, variable importance measure. The positions illustrated here correspond to the results in Tables 1 and 2 for the presence of residues at specific sites.

The majority of the top-ranked features for the two outcomes pertain to presence/absence of specific Env residues, with the most important residues located at CD4 contact sites shown previously to be associated with VRC01 neutralization sensitivity or resistance. The most important of the features predictive of non-censored IC_50_ (which we refer to here as neutralization sensitivity) were an arginine at position 456 (R456) and a glycine at position 459 (G459), which were identified as top-ranked features for both outcomes and by both VIM methods; when referring to an AA present at a given position, we give the one-letter code of the AA followed by its position in HBX2 coordinates. Other highly ranked features predictive of sensitivity were N280 (top-ranked by both VIM methods for IC_50_ censored), G458 (top-ranked by the algorithm-specific method for the IC_50_ censored outcome), and D279 (top-ranked by the algorithm-specific method for the log IC_50_ outcome). The most highly ranked residue predictive of neutralization resistance was I471 (highly ranked by both VIMs for both outcomes). In total, 16 residues predictive of neutralization sensitivity and 10 residues predictive of neutralization resistance made the top-ranked list in Table 1. Of these, 6 (37.5%) residues predictive of neutralization sensitivity and 3 (30%) residues predictive of neutralization resistance also made the top-ranked list in Table 2. Visualizations of the locations, magnitudes, and distributions of the most predictive residues in Tables 1 and 2 are provided in Fig 6. Clusters of predictive sites are found just prior to and within the V5 variable loop, and within Loop D. The logo plots in Fig 6C and 6D show the distributions of amino acids within neutralization-sensitive and -resistant viruses, respectively. These figures demonstrate that, with the majority of the predictive sites, minority mutations are entirely or strongly associated with resistance (e.g., with anything but arginine (“R”) at site 456 conferring VRC01 resistance), and that no strong discriminating signal exists at the individual site level, implying that a multivariate predictor would be necessary for effective performance.

Besides site-specific residue information, other AA sequence features were also highly ranked. A longer length (in AAs) of Env and of gp120 was found to be important for predicting resistance for both outcomes, and more cysteines in Env were found to be important for predicting resistance in the IC_50_ censored outcome. For the dichotomous outcome, the presence of a PNGS at either site 156 or site 616 was important for predicting sensitivity, and the presence of a PNGS at either site 229 or site 824 was important for predicting resistance. The number of PNG sites in the V5 region was important for predicting neutralization sensitivity, for the dichotomous outcome only, and subtype A1 was also important for predicting neutralization sensitivity, for the log IC_50_ outcome only. VIM results for the other three outcomes are given in S7 Table.

In addition to evaluating the predictive importance of individual AA sequence features, the ensemble-based Super Learner approach also estimated VIMs for the groups of pre-specified AA sequence features (S11 Fig) by feature group. This analysis showed that the group of AAs in the VRC01 binding footprint and the CD4 binding sites were the most important predictive groups, a result found for both the dichotomous and the log IC_50_ outcome.

#### Output of the analysis

Based on the above analysis, all of the features listed in Tables 1 or 2 may be included in the primary AA sequence sieve analysis that assesses how prevention efficacy depends on the Env variants of each feature.

### Exploratory sensitivity analyses

#### Analysis and feature selection using lasso

Although this study evaluates the use of Super Learner against its component learners, one easily interpretable model for defining a PAR score is the “lasso (using geography and all features pre-selected by lasso)” (See Methods, Envelope amino acid feature input variable groups) method applied to the IC_50_ censored outcome, which we can use as an example to investigate how many of the total features would be selected for analysis, and how they contribute to classification. This method performed well on both datasets, and received large weight in the Super Learner ensemble in several instances. To explore this model further, we fit this selected model to the whole dataset (sets 1 and 2 combined), using the penalty that minimizes the 10-fold cross-validated deviance, and the resulting model had 80 non-zero coefficients, showing that many AA features contribute to classification of neutralization resistance (S6 Table).

#### Classifying IC_50_ censored with lasso using a reduced feature space

In a related sensitivity analysis, we sought to determine whether we could achieve equivalent classification performance with only a small number of features and using a simple estimator. Extending the above sensitivity analysis, we used the lasso as our estimator, and used the top five features selected by this lasso on each dataset. For the model built using dataset 1, the selected features were R456, G459, "subtype is C", "total cysteines in Env", and G132. For the model built using dataset 2, the selected features were R456, G459, "subtype is C", N380, and I471. We then built a predictive lasso model for each dataset of the IC_50_ censored outcome using only these five features and validated this model against the second dataset. The simple lasso model built on dataset 1 was validated on dataset 2 with an AUC of 0.865; the simple lasso model built on dataset 2 was validated against dataset 1 with an AUC of 0.845. The results of this simple approach with a small number of features are nearly equivalent to the performance of the “lasso (using geography and all features pre-selected by lasso)” method described above that yielded 80 non-zero coefficients, which had AUCs of 0.843 (0.779, 0.908) when trained using dataset 1 and validated against dataset 2, and 0.866 (0.806, 0.925) when trained using dataset 2 and validated against dataset 1. This sensitivity analysis suggests that using a simple estimator and a small number of important features performs nearly as well as the estimator using the full set of predefined features.

#### Classifying IC_50_ censored with lasso using the top selected features from the VIM analysis

We sought to determine whether using the top selected features of the VIM analysis in Table 1 in a lasso model would yield similar classification performance to our previous sensitivity analysis (using the top five lasso-selected features only). We also sought to determine the minimum number of features required to achieve equivalent performance. Starting with the top five VIM-determined features and working incrementally through the top ten VIM-determined features, we built a lasso model for each dataset of the IC_50_ censored outcome and validated the model against the respective hold-out dataset. The classification of this endpoint achieved equivalent performance as the full model using all features with the top seven VIM-determined features, achieving AUCs of 0.861 (when trained on dataset 1 and validated on dataset 2) and 0.841 (when trained on dataset 2 and validated on dataset 1). In contrast, the top six (or fewer) VIM-determined features achieved a slightly reduced performance, with hold-out validated AUCs of 0.824 and 0.794 for training sets one and two, respectively. The equivalent performance from a simple lasso model using the top seven features selected by the VIM analysis suggests that these seven VIM-identified features may account for a large proportion of the performance of the full model.

#### Assessing for phylogenetic confounding

Our analyses have corrected for potential HIV-1 phylogenetic confounding by always controlling for geographic region, which we acknowledge is a somewhat limited approach. In light of this, we conducted another sensitivity analysis with more-refined confounding control based on phylogenetic trees. A neighbor-joining tree was built from all the Env protein sequences using DIVEIN [40], and using the pairwise phylogenetic divergence, we identified the pair of sequences with the closest distance; the sequence in this pair with the shortest mean pairwise divergence to all other sequences was removed from the population, and the process repeated until all sequences had a pairwise divergence greater than 0.05. This reduction process identified 76 sequences (12.4%) for removal, resulting in a total of 535 sequences. Starting with the same two analysis data sets that were used by our primary analysis, removing these sequences resulted in two new analysis data sets of size n = 275 (set 1) and n = 260 (set 2). Classifying the dichotomous IC_50_ censored endpoint, the best model with set 1 yielded a CV-AUC of 0.863 (validating on set 2 with an AUC of 0.913), and the best model with set 2 yielded a CV-AUC of 0.870 (validating on set 1 with an AUC of 0.786). The performance of these models is similar to those that were trained on whole datasets, implying that our modeling process is not majorly affected by phylogenetic confounding.

## Discussion

Excellent research has been done to understand HIV-1 Env genotypic/AA features that affect VRC01 resistance [2, 9, 41–44]. Our approach complements this work by applying state-of-the-art machine learning and methodology for unbiased, nonparametric statistical inference (our area of expertise) − a contribution not yet provided in previous computational approaches to define AA sequence signatures for various bnAbs [7, 45–49] − and then interpreting the results in comparison with those from literature. Other advantages of our modeling approach are that it (i) combines the strengths of several machine learning algorithms to improve prediction accuracy in an automated, unbiased way; and (ii) bases all decisions on hold-out validation, where only data not used in model building is used for measuring predictive performance and for selecting features.

Within each of two separately analyzed halves of the CATNAP data, we used the metrics of cross-validated AUC and cross-validated nonparametric R^2^ for summarizing prediction accuracy to compare the performance of different learners and feature sets, with 95% confidence intervals that are valid in the context of “inference after model selection” [38]. We then applied models fit with one dataset to the other completely separate dataset, validating their performance and effectively providing a simple conclusion of replicability of the results. In addition, our novel Super Learner-based variable importance measure (VIM) has a useful incremental predictive value interpretation for a given feature, as the additional proportion of variance in the outcome explained by including the feature in addition to including all other features [50]. An advantage of this nonparametric VIM is that its interpretation does not require any particular parametric model for the data to hold, in contrast to methods such as logistic regression.

In our first objective (model selection), we identified several models via machine learning that provided strong and similarly accurate performance to classify viruses (based on specific AA sequence features in Env) as resistant vs. sensitive, measured by whether neutralization IC_50_ is right-censored or by restricting the “sensitivity” category to IC_50_ < 1 μg/mL. Due to its advantageous theoretical properties, strong performance in simulation studies and data analyses, and consistently high performance in the present work, the Super Learner is the learning approach that will be advanced to the primary sieve analysis. We have already created a preliminary Super Learner-based model to predict neutralization sensitivity of virus sequences obtained from HIV-1 infections occurring during the AMP trials. When predicting the quantitative endpoints, our models had weaker performance than those built to classify the dichotomous outcomes. It is possible that the dichotomous IC_50_ outcome is easier to classify because the value “censored” has clear meaning as neutralization activity not being detected in the experiment [8], whereas log IC_50_ and log IC_80_ may have more noise due to natural variability in the TZM-bl assay and the assignment of specific numeric values to censored values. In addition, of the three quantitative outcomes studied, prediction performance was best for log IC_50_, intermediate for log IC_80_, and worst for neutralization slope. This may be explained in part because 29.6% percent of the 611 viruses in CATNAP were missing data on IC_80_ (and by extension, neutralization slope), and perhaps the missing data is contributing to a diminished predictive signal. In addition, the slope readout is a ratio-readout with the additive difference term log IC_80_ –log IC_50_ in the denominator, such that if noise makes IC_80_ close to IC_50_, then the denominator is small, and the impact of noise is amplified. This can occur because each of IC_80_ and IC_50_ are estimated imperfectly based on a percent neutralization by concentration curve (with IC_50_ estimated with somewhat more precision given the fiftieth percentile is in the center of the percent neutralization distribution). Thus, it remains an open question whether slope is a meaningful neutralization outcome.

Our novel findings in objective 2 pertain to the variable importance measures of individual AA sequence features, which identified 14 specific residues at certain AA sites (M181, E279, S280, T280, C397, R425, M428, Q455, H456, S456, W456, D459, I471, and N655) and 6 other AA sequence features (longer gp120, longer Env, more cysteines in gp120, more cysteines in Env, and PNG sites at 229 and 824) that had high variable importance for predicting neutralization resistance. We also identified 18 specific residues (P124, N156, L179, D279, N280, S365, H374, N425, Q428, R456, D457, G458, G459, E466, R469, G471, T569, and D589) and 4 other AA sequence features (PNGS at sites 156 and 616, more PNG sites in V5, and subtype A1) that had high variable importance for predicting neutralization sensitivity.

Most of the important features identified in this study were based on the documented VRC01 footprint and the CD4 binding sites, which largely overlap with each other. Seven of the predictive residues that we identified, however, fall outside of these regions. Three of these seven sites were included for analysis because of their covariation with sites within the VRC01 footprint: L179 (lending to VRC01 sensitivity), and M181 and C397 (lending to VRC01 resistance). The latter finding is interesting, in that it is rare to find cysteines in the surface-exposed region of gp160 outside the context of the 10 canonical disulfide bond-forming pairs (described in [51]). Neither site 179 nor site 397 has been described in the literature to be associated with VRC01 activity, although glycans at 397 have been found to be important for the binding activity of other bnAbs [52].

Three of the four remaining sites identified as predictive by this study were included as part of the sites in gp41 that have been found to be associated with VRC01 binding: T569 and D589 were associated with increased sensitivity, and N655 was associated with resistance. These results agree with previous reports of similar mutations altering neutralization sensitivity globally [53–55], and highlight how variation in the HR-1 and HR-2 domain of gp41 can modulate sensitivity to neutralization by VRC01.

The final site identified by this study is N156 (lending to VRC01 sensitivity), which was included as part of Group 6, based on the probability that the presence or absence of a PNGS at this site would result in improved or reduced VRC01 binding [56]. Indeed, site 156 was observed to be a PNGS in 94% (576 of 611) of the CATNAP sequences included in this study, where disruption of the PNGS motif was more likely to confer VRC01 resistance: 51% of sites without a PNGS at site 156 were found to be VRC01 resistant, while only 14% of sites with a PNGS at site 156 were VRC01 resistant. A glycan at this position has been hypothesized as being important for recognition by other bnAbs [57], but to the best of our knowledge this is the first report to associate N156 with sensitivity to neutralization by VRC01.

Many of the residues we identified as highly predictive of at least one of the outcomes are supported by experimental evidence as being important for VRC01 binding. Four of the top-ranked AAs found in this study (D279, N280, R456, and G459) have been shown to be sites of common interactions with potent VRC01-like Abs [58], and D279 and E459 have been identified as making critical interactions with VRC01 [9, 52]. Moreover, mutation of residue D279 to E279 (D279E) was shown to be part of the VRC01 escape pathway within the donor from whom VRC01 was isolated [41]. For objective 2, we also found that AA sequence features in the VRC01 binding footprint sites and in the CD4bs have greatest variable importance, a result that is not surprising given previous work. This finding supports conducting AMP sieve analyses that focus on groups of AA sites that define these two regions.

Diverse approaches have been taken to identify Env sequence patterns associated with bnAb neutralization sensitivity (bnAb signatures) [7, 45–49]. We next discuss our work in the context of sequence-based approaches that have been taken to predict sensitivity to VRC01-mediated neutralization. Using non-linear support vector machines to predict neutralization sensitivity of pseudoviruses with different Env AA sequences, Hake and Pfeifer identified N186, N276, N279, N280, G459, and K232 as strong predictors of susceptibility to VRC01-mediated neutralization [59]. Of these 6 AAs, we found that G459 ranked extremely highly for contribution to prediction to 4 out of the 5 outcomes (Table 2, S6 Table). N280 also ranked highly for contribution to prediction of the quantitative log IC_50_ outcome (Table 2). Moreover, considering that for each of the outcomes, only a small number of sites (between 2 to 4 for each outcome) met our criteria for being highly predictive of a given outcome (high VIMs across both methods, a low FWER p-value), and that our sensitivity analysis was able to achieve equivalent classification performance of the IC_50_ censored outcome with only five features (see Results), our results support the overall conclusion of Hake and Pfeifer that, in general, only a few key residues are needed to well-predict neutralization resistance.

While we have reported our specific findings based on all available VRC01 CATNAP data as of the initiation of this work in March 2017, the CATNAP database is being continuously updated to include new results in the scientific literature; at the time of this writing, the CATNAP database includes 54 neutralization results that were not available when the datasets for this analysis were finalized. When finalizing the plan for the AMP sieve analysis (expected in 2020), we plan to re-run this predictive analysis with an up-to-date version of the CATNAP database, to (a) ensure that our selected PAR scores are based on the maximum number of pseudoviruses/sequences; (b) verify that the most predictive AA features remain the same, and (c) update our analysis plans accordingly if new AA features are found that outperform the top-performing AA features identified here. We will also consider including AA sequence features identified by others in complementary analysis approaches.

We now discuss the limitations of this study. To maximize our sample size, we used all available sequences with TZM-bl neutralization data in the CATNAP database, regardless of their subtype. The AMP trials, however, are conducted in regions where circulating HIV-1 viruses are largely subtypes B (Americas and Switzerland) and C (southern Africa). As such, the results of this analysis may be influenced by characteristics of viral subtypes that will not play a role in the AMP trials; however, we note that our analysis did assess whether subtype helped predict neutralization resistance, and only subtype non-A1 vs. subtype A1 was found to be an important feature (while subtype C was associated with greater neutralization resistance, its variable importance measures did not rank it among the most predictive features).

While the TZM-bl assay is validated and the multiple labs contributing data to CATNAP undergo certification through proficiency testing, nevertheless it is common for different labs to produce IC_50_ or IC_80_ readouts with two-to-three-fold variation on the same samples [60]. This variability creates noise in the outcome variable that dampens prediction accuracy. In addition, the outcome predicted best by our models–whether the IC_50_ outcome was reported as right-censored (i.e., resistant) in the original publication cited by CATNAP [8]–has noise stemming from unknown differences among labs in factors that were considered in defining the outcome.

Another limitation of our approach is that we considered prediction of neutralization sensitivity of a single Env pseudovirus based on its gp160 AA sequence, but some AMP efficacy trial participants are expected to be infected with multiple founder viruses. How to properly account for the number of founders and the accompanying multiple gp160 sequences in predicting neutralization sensitivity of an exposing viral quasispecies is an open question for future research. An additional caveat is that we only analyzed VRC01 neutralization readouts obtained by one particular assay, which uses TZM-bl target cells and is performed *in vitro*, only approximating a real-life exposure event of the genital mucosa to HIV-1 in the presence of VRC01; however, given that this assay is the standardized and validated platform for HIV-1 vaccine evaluation, developing predictors of this assay’s readouts is an important goal, with a test of *in vivo* validation forthcoming from the AMP sieve analysis. Prior evaluation of the ability of multiple bnAbs to prevent HIV-1 infection using a mucosal tissue explant model has shown that neutralizing activity as assessed by the TZM-bl assay is moderately correlated with inhibitory activity in penile and cervical tissue, but not correlated with inhibitory activity in colorectal tissue [61]. In addition, VRC01 may protect against HIV-1 acquisition in additional ways not captured by a neutralization assay, such as via non-neutralizing Fc effector functions. In support of this idea, VRC01 (or serum from participants infused with VRC01) has been shown to mediate low levels of antibody-dependent cell-mediated cytotoxicity [62] and higher levels of antibody-dependent cellular phagocytosis (ADCP) of gp140-coated microspheres and of virion capture [63], which may also be important for preventing HIV-1 acquisition. These findings make it of interest in future work to build models predicting ADCP and other non-neutralizing Fc effector functions based on AA sequence features.

With this study, we have created and applied modeling tools to help design the primary AA sequence sieve analysis in AMP, such that the analysis will be based on the hypothesis that Env AA-based predictors of *in vitro* resistance measured by the TZM-bl assay will also discriminate prevention efficacy. For each of the top-ranked features we identified, the AMP sieve analysis could test whether the level of prevention efficacy differs across HIV-1 variants of the feature. Beyond preparation for sieve analysis in bnAb prevention efficacy trials, another application of our predictive modeling framework includes scoring AA signature types for bnAb resistance, prior to using a particular bnAb as a therapeutic in an HIV-1 infected individual.

## Methods

### Dataset

A total of 624 Env viral AA sequences, their associated pseudovirus IC_50_ and IC_80_ values for neutralization by VRC01 as assessed by the TZM-bl assay, and other associated annotations were retrieved from the CATNAP database [8]. [The 50% and 80% inhibitory concentrations (IC_50_ and IC_80_, respectively) are defined as the concentration of VRC01 required to cause either a 50% or 80% reduction in Env pseudovirus replication (as measured in relative luminescence units) relative to the level of replication in the absence of VRC01. This reduction in replication in the presence of VRC01 is inferred to be a consequence of VRC01-mediated neutralization. Hence, a low IC_50_ or IC_80_ value for VRC01 indicates that the given Env pseudovirus is relatively sensitive to VRC01-mediated neutralization, whereas a higher or right-censored IC_50_ or IC_80_ value indicates that the given Env pseudovirus is relatively resistant to VRC01-mediated neutralization.] Some of the provided annotation was unstructured or unsuitable for analysis, so these data were refactored appropriately. (Additional details about the data processing step can be found in the S1 Text.) Thirteen sequences/pseudoviruses were excluded from the analysis, because their IC_50_ measurement was recorded as right-censored at 1 μg/ml. According to the study that produced these results [64], this unusually low limit of censorship was due to a lack of reagent. As such, we excluded these pseudoviruses from the study, as their neutralization results were regarded as unreliable.

This resulted in a total of 611 sequences/pseudoviruses to include in the analysis, of which 48.0% (293) were subtype C (the predominant subtype in the HVTN 703/HPTN 081 trial) and 13.3% (81) were subtype B (the predominant subtype in the HVTN 704/HPTN 085 trial) (S8 Table). Of these 611 pseudoviruses, all 611 had quantitative log IC_50_ neutralization readouts, which means that the IC_50_ censored outcome and the quantitative log IC_50_ outcome (defined below in “TZM-bl resistance outcomes used in this analysis”) were available for all 611 of them. We were able to derive a sensitive/resistant only outcome for 474 (77.6%) of these pseudoviruses, and 430 (70.4%) of the pseudoviruses had an IC_80_ neutralization readout, which means that we were only able to derive quantitative log IC_80_ and neutralization slope outcomes for these 430 pseudoviruses. All of the data used in this analysis, including the identifiers of the sequences and their outcomes used, are posted at https://github.com/benkeser/vrc01/tree/1.0.

The restructured dataset was randomly partitioned into two datasets (“dataset 1” and “dataset 2”) for the statistical learning analyses. The two datasets were mutually exclusive, each with half of the data [n = 306 (dataset 1) and n = 305 (dataset 2)]. The random partitioning process was stratified by the viruses’ country of origin. We chose to stratify the data by country instead of HIV-1 subtype because subtype was to be included as an input feature in the analysis, as it was considered to be a potential sequence-based predictor of resistance, whereas country was not used as an analytical feature but was controlled for as a potential confounder. Of the 611 HIV-1 sequences analyzed, 51.9% (317) originated from a country in which one or more study sites for the HVTN 703/HPTN 081 trial are located and 9.5% (58) originated from a country in which one or more study sites for the HVTN 704/HPTN 085 trial are located (S8 Table). The geometric means of the imputed log_10_ IC_50_ values of the pseudoviruses whose Env sequences were included in this analysis are shown by region/subtype in S12 Fig.

### Envelope amino acid feature input variable groups

AA sites of potential relevance to VRC01-mediated neutralization of HIV-1 were included as input features for the statistical learning analyses. For features defined by residue content at a given AA site, only AA sites passing a minimum variability filter were included. Specifically, the residue had to differ from the consensus residue in at least 3 sequences in the entire analysis dataset (i.e., before splitting into the two analysis sets). Furthermore, indicators for the presence of a residue at a specific site were only included if that residue was present in at least three viral sequences at that site across the entire dataset.

Those sites that passed the minimum variability requirement were included in the analysis if they fell into any of the following pre-defined groups (many sites are found in more than one pre-defined set):

**Group 1) VRC01 binding footprint sites**, corresponding to the 35 AA positions in gp120 identified in [2] as contact sites between VRC01 and HIV-1 Env;
**Group 2) CD4 binding sites**, corresponding to all AA positions that constitute the CD4 binding site as defined in [2];
**Group 3) Sites with sufficient exposed surface area** (ESA) as calculated by the DSSP program [65, 66] using crystal structures of VRC01 in complex with clade A/E gp120, clade A gp120, clade C gp120, clade G gp140, and clade B gp140 (PDB IDs 3NGB [2], 4LSS [67], 4LST [67], 5FYJ [68], and 5FYK, respectively) (further details are given in S1 Text);
**Group 4) Sites identified as important for glycosylation**, including AA positions related to the glycan fence identified in [68–70] or identified in [68] as sites where VRC01 interacts with the Env trimer;
**Group 5) Sites with residues that covary with the VRC01 binding footprint sites**, corresponding to all gp120 AA positions (excluding those in the signal peptide) not included in **Groups 1**–**4** that are outside the VRC01 footprint (defined in [2]) and that statistically covary with at least one footprint position, based on the normalized mutual information statistic and an accompanying test for whether there is covariability [71] (further details are given in S1 Text);
**Group 6) Sites associated with VRC01-specific potential N-linked glycosylation (PNGS) effects**, identified by a Bayesian machine learning approach that assessed bnAb binding against a panel of 94 recombinant gp120 monomers [56], where the presence or absence of a PNGS results in improved or reduced VRC01 binding; and
**Group 7) Sites in gp41 associated with VRC01 sensitivity or resistance**, corresponding to the 13 sites identified in [53–55, 72–78].

In addition to AA sites, the following features within feature groups were also included as input features:

**Group 8) Indication of potential N-linked glycosylation sites (PNGS)**. A binary indicator was provided for sites in all of Env that featured the canonical N-linked glycosylation motif ([N][!P][S|T]) [79] in at least 3 of the analysis sequences (and absent from at least 3 of the analysis sequences). PNGS indicators were not included for sites that are insertions relative to HXB2, since these sites are difficult to reproduce precisely in a different alignment.
**Group 9) Majority virus subtypes:** Virus subtype was included for all subtypes present in at least 10 database sequences for the entire dataset. These subtypes include CRF01_AE, CRF02_AG, CRF07_BC, A1, A1C, A1D, B, C, D, O. All sequences with a subtype represented by fewer than 10 sequences (n = 44, 7.2% of total) were grouped into the subtype category of “Other”. This information was represented in the data as binary indicator variables for each subtype (including “Other”).
**Group 10) Region-specific counts of PNG sites,** corresponding to the numbers of PNG sites as defined by the canonical N-linked glycosylation motif ([N][!P][S|T]) [79] among 10 different site sets (site sets are described in S1 Text). This information was included under the rationale that subtype C neutralization resistance has been shown to be strongly associated with high glycan density [80];
**Group 11) Viral geometry**, corresponding to the total lengths of five different regions within the Env sequence known to be important for VRC01 binding: the entire Env polyprotein, the entire gp120 protein, the V5 region, Loop D, and Loop E (additional detail in S1 Text);
**Group 12) Cysteine counts**, corresponding to the numbers of cysteines present within five different regions of the Env sequence: the entire Env polyprotein, the entire gp120 protein, the V5 region, Loop D, and Loop E (additional detail in S1 Text); and
**Group 13) Steric bulk at critical locations**, corresponding to the total number of “small” (as defined in [81]) residues in the V5 region, in Loop D, and in the CD4 binding loop. This group is based on the rationale that much natural resistance to VRC01 appears to be due to steric clashes, especially in these loops [41, 42, 82].

**Fig 7:**
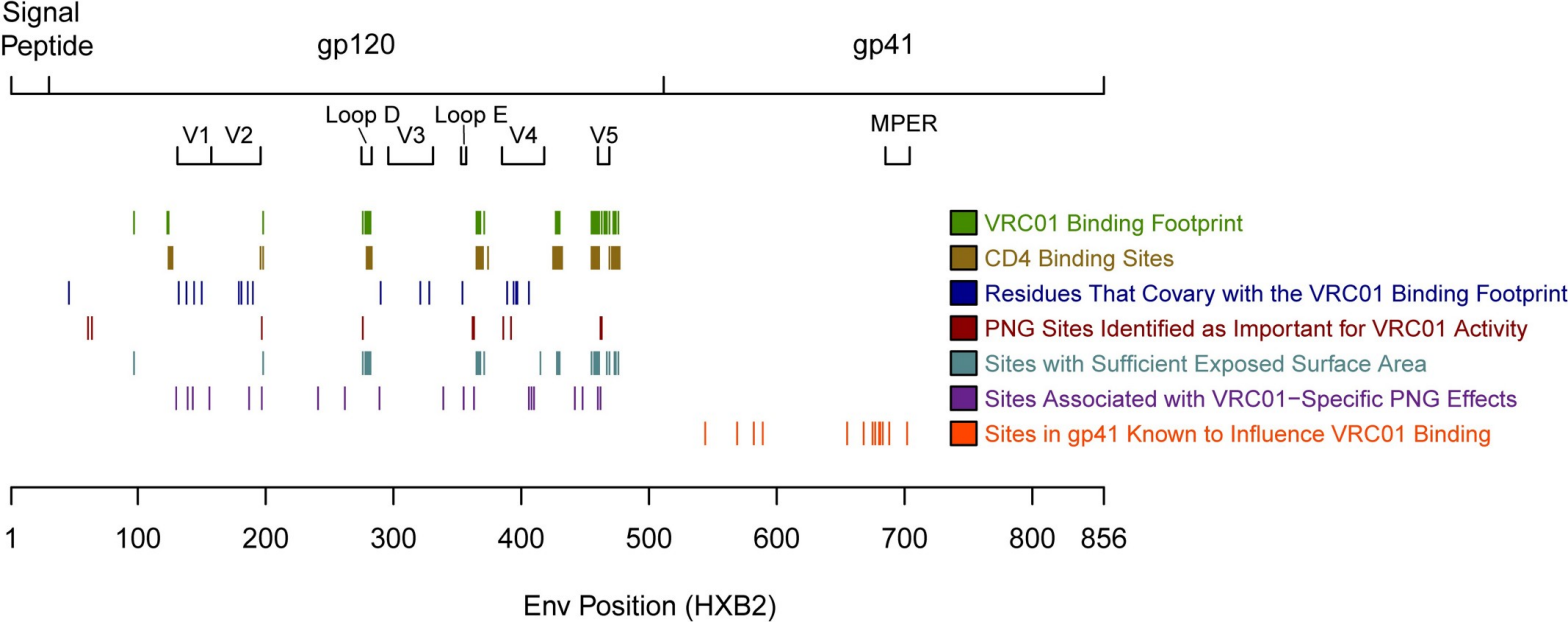
Specific sites in Feature Groups 1 to 7 before application of the minimum variability filter.

All of these groups, and the sites contained within them, are outlined in Table 3A. Fig 7 provides a schematic visualization of the AA sites in Feature **Groups 1–7** before application of the minimum variability filter (specific sites in each group are listed in S1 Text).

**Table 3:** Distinct input variable sets used for the machine learning analyses, and learning algorithm types.

A. Distinct input variable sets used for the Super Learning analyses	
**Variable set name**	**Variables**^**1**^
1: geog	Geographic region (Asia/Europe and Americas/N. Africa/S. Africa)
2: geog.AAchVRC01	geog + Group 1^2^ (AAs in the VRC01 binding footprint) (97, 124, 198, 276, 278, 279, 280, 281, 282, 365, 371, 428, 429, 430, 455, 456, 458, 459, 460, 461, 463, 465, 466, 467, 474, 476)
3: geog.AAchCD4bs	geog + Group 2 (AAs in the CD4 binding site) (124, 125, 198, 279, 280, 281, 282, 283, 365, 369, 374, 425, 426, 428, 429, 430, 432, 455, 456, 458, 459, 460, 461, 471, 474, 475, 476, 477)
4: geog.AAchESA	geog + Group 3 (AAs with sufficient Exposed Surface Area) (97, 198, 276, 278, 279, 280, 281, 282, 365, 371, 415, 428, 429, 430, 455, 458, 459, 460, 461, 467, 474, 476)
5: geog.AAchGLYCO	geog + Group 4 (AAs important for glycosylation) (61, 197, 276, 362, 363, 386, 392, 462, 463)
6: geog.AAchCOVAR	geog + Group 5 (AAs that covary with the VRC01 binding footprint) (46, 132, 138, 144, 150, 179, 181, 186, 190, 290, 321, 328, 354, 389, 394, 396, 397, 406)
7: geog.AAchPNGS	geog + Group 6 (AAs associated with VRC01-specific PNGS effects) (130, 139, 143, 156, 187, 197, 241, 289, 339, 355, 363, 406, 408, 410, 442, 448, 460, 462)
8: geog.AAchgp41	geog + All gp41 sites that affect global neutralization sensitivity (544, 569, 582, 589, 655, 668, 675, 677, 680, 681, 683, 688, 702)
9: geog.AAchGlyGP160	geog + All gp160 N-glycosylation sites that are not included in VRC01 contact sites or paratope or sites with covariability
10: geog.st	geog + Group 8 (viral subtypes) (01 AE/02 AG/07 BC/A1/A1C/A1D/B/C/D/O/Other)
11: geog.sequonCt	geog + Group 9 (region-specific PNGS counts)
12: geog.geom	geog + Group 10 (viral geometry metrics)
13: geog.cys	geog + Group 11 (counts of cysteine residues in certain regions)
14: geog.sbulk	geog + Group 12 (steric bulk at critical locations)
15: geog.corP	geog + features selected with t-test univariate p-values
16: geog.glmnet	geog + features selected with non-zero coefficients based off lasso
17: geog.all.MCCV	All variables in sets 1–13, described above (AAs as positions 46, 61, 97, 124, 125, 130, 132, 138, 139, 143, 144, 150, 156, 179, 181, 186, 187, 190, 197, 198, 241, 276, 278, 279, 280, 281, 282, 283, 289, 290, 321, 328, 339, 354, 355, 362, 363, 365, 369, 371, 374, 386, 389, 392, 394, 396, 397, 406, 408, 410, 415, 425, 426, 428, 429, 430, 432, 442, 448, 455, 456, 458, 459, 460, 461, 462, 463, 465, 466, 467, 471, 474, 475, 476, and 477, plus all features in Groups 8 through 12)
B. Learning algorithm types and the distinct input variable groups used with each learner	
**Algorithm type**^**3**^	**Input variable groups from 3A**
SL.randomForest	1,2,3,4,5,6,7,8,9,11,12,13,14,15,16
SL.glmnet	1,2,3,4,5,6,7,8,9,11,12,13,14,15,16
SL.xgboost	1,2,3,4,5,6,7,8,9,11,12,13,14,15,16
SL.naiveBayes	1,2,3,4,5,6,7,8,9,11,12,13,14,15,16
SL.glm	1,10,11,12,13,15,16
SL.step	1,10,11,12,13,15,16
SL.step.interaction	1,10,11,12,13,15,16
SL.mean	None

geog = geography; AA = amino acid. AA positions are given in HXB2 coordinates.

^1^All amino acids included in the variable sets met the minimum variability filter that the site had to differ from the consensus site in at least 3 sequences in the entire CATNAP data set (i.e. before splitting into the two analysis sets).

^2^See Methods for details on listed input variable Groups 1−13

^3^The algorithms are listed by the functions used in the SuperLearner R package. An exception is “SL.naiveBayes”, which was a custom wrapper designed to use the naiveBayes function from the e1071 package. The SL.glmnet package was used with the lasso penalty. All tuning parameters are set to the default values of the SuperLearner package, except SL.xgboost, which we modified to fit decision stumps rather than trees.

### TZM-bl resistance outcomes used in this analysis

For this analysis, new univariate IC_50_ and IC_80_ outcome variables for each pseudovirus were created that incorporate imputations from the right-censored IC_50_ and IC_80_ values retrieved from CATNAP and that account for occasions that values were reported from multiple studies. This process is described in detail in S1 Text.

Using these IC_50_ and IC_80_ univariate outcomes, the statistical learning processes aimed to predict from the AA sequence features five TZM-bl neutralization resistance outcomes: (1) dichotomous resistance outcome of whether the IC_50_ is right-censored (the “IC_50_ censored” outcome) as recommended in [8]; (2) dichotomous resistance outcome (the “sensitive/resistant only” outcome), with resistance defined by the IC_50_ being right-censored as in (1) and sensitive defined as IC_50_ < 1 μg/ml; (3) log_10_ IC_50_ resistance outcome as a quantitative measure (the “quantitative log IC_50_” outcome); (4) log_10_ IC_80_ resistance outcome, also as a quantitative measure (the “quantitative log IC_80_” outcome); and (5) estimated dose-response curve slope of neutralization, which is a function of IC_50_ and IC_80_, calculated as in equation (6) of [83], equal to log(4) divided by (log IC_80_ –log IC_50_ (the “neutralization slope” outcome). A caveat of the “IC_50_ censored” outcome analysis is that 50 pseudoviruses that were included were right-censored at value IC_50_ > 10 μg/ml (no pseudoviruses right-censored at a lower value were included), yet some pseudoviruses had observed IC_50_s only incrementally larger than 10 μg/ml (e.g., 14 pseudoviruses had IC_50_ greater than 10 μg/ml and less than 50 μg/ml); this issue could add noise to the analysis.

### Statistical learning approaches

#### Creating proteomic antibody resistance (PAR) scores for use in the primary statistical amino acid sequence sieve analyses of the AMP trials (Objective 1)

We applied several statistical learning techniques to determine the best prediction function for each outcome. These learning techniques included modern machine learning approaches: lasso [84] (with identity link for continuous outcomes and logistic link for dichotomous outcomes, implemented in the glmnet R package [85]), random forests [86] (implemented in the randomForest R package [87]), Naïve Bayes [88] (for dichotomous outcomes only, implemented in the e1071 R package [89]), and boosted decision stumps via extreme gradient boosting [90] (implemented in the xgboost R package [91]). We also included classical statistical techniques: generalized linear models and stepwise-selected generalized linear models (again with identity link for continuous outcomes and logistic link for dichotomous outcomes, implemented in the glm R package [92]). Each learning approach was combined with different variable screening techniques, resulting in a total of 82 candidate learning algorithms for each dichotomous outcome, and 67 candidate models for each continuous outcome (listed in Table 3B). We also considered a convex ensemble of all learners using regression stacking [33, 93], also known as Super Learning [34]. In this approach, the candidate learning algorithms are combined by convex weights chosen to minimize a cross-validated prediction criterion (in this work, we used mean squared error for continuous outcomes and average negative log-likelihood loss for dichotomous outcomes). This allows the contribution of each candidate learning algorithm to be between zero and one, with the weights summing to one. Since the Super Learner estimator is a convex combination of the individual candidate learners, the Super Learner estimator is as nonparametric as the most nonparametric estimator included in the Super Learner algorithm [34]. In particular, including random forests and boosted decision stumps in our library of candidate learners makes our Super Learner estimator highly flexible.

The following analyses were done separately for each of the two partitioned datasets. Each candidate learner and the Super Learner were evaluated on each dataset via internal 10-fold cross-validation. That is, each of the two datasets were split into ten folds and cross-validated prediction metrics were computed on each dataset. We note that in computing these measures for the Super Learner, which itself utilizes cross-validation, it was necessary to perform nested cross-validation. For dichotomous outcomes, we computed the cross-validated area under the receiver operating characteristics curve (CV-AUC), while for quantitative outcomes, we computed the cross-validated nonparametric R-squared (CV-R-squared or CV-R^2^), defined as one minus the ratio of cross-validated mean squared error and the variance of the outcome. The CV-R^2^ measures the proportional reduction in mean squared-error when predicting the outcome using a given learner versus predicting the outcome using its mean. Thus, values close to one indicate that a learner nearly perfectly predicts the value of the outcome, values close to zero indicate that a simple average of the outcome predicts the outcome about as well as the learner, and values below zero indicate that a simple average predicts better than the learner. Wald-type 95% confidence intervals about CV-AUC and CV-R^2^ were computed using influence function-based standard error estimates [38]. This influence function-based approach allows us to compute valid confidence intervals for the true CV-AUC and the true CV-R^2^ on both the training and validation sets [38].

Proteomic antibody resistance (PAR) scores are defined for given gp160 AA sequences as the predictions generated by the models that performed best in terms of CV-AUC or CV-R^2^ consistently across the two datasets and validated against the separate dataset (we ended up selecting the Super Learner model), and used as a predictor of VRC01 sensitivity to study as a discriminator of prevention efficacy in the AMP sieve analysis. When the HIV-1 sequences from infected trial participants are available for the AMP sieve analysis, the final Super Learner predictive model for creating the PAR scores will be updated by re-fitting the model to the entire available CATNAP dataset.

#### Assigning variable importance measures (VIMs) to AA sequence features in the input set and thresholds for inclusion in the analysis (Objective 2)

To determine a group of gp160 AA features to be included in the primary sieve analysis for AMP, each individual feature and each pre-defined group of features (see “Input variable groups” above) were assigned VIMs for each of the five TZM-bl neutralization outcomes and each dataset (datasets 1 and 2). Two approaches were taken to define variable importance: an algorithm-specific approach and an ensemble-based approach. The arithmetic mean was then taken of VIMs across all learners. This resulted in a single composite VIM for each feature, in the range of 0–100, for each of the five outcomes.

In the algorithm-specific approach, Monte Carlo cross validation (MCCV) was used with each of the two data sets, using 1000 iterations and an 80/20 training/test split. Variable importance was defined using the commonly used importance metric for each learning algorithm. Specifically, the VIMs for each learner are: the number of iterations where the feature was selected over the MCCV runs (for the lasso and XGboost); the out-of-bag variable importance measure for mean decrease in accuracy (for random forests); and the inverse log-10 of the p-value of the balance of the probabilities for each variable (Naïve Bayes). This yielded one VIM value for each unique combination of dataset, algorithm, outcome, and feature.

To fulfill Objective 2 based on the algorithm-specific VIMs, the VIMs for each algorithm/outcome/dataset combination were linearly rescaled across features to a range of 0−100. The two VIM values computed on the two datasets were consolidated using the geometric mean, so that each learning method and outcome had a single VIM for each feature; using the geometric mean penalized a feature for having discordant VIMs across the two datasets. The arithmetic mean was then taken of the VIMs across all learners. This resulted in a single composite algorithm-specific VIM for each feature, in the range of 0–100, for each of the five outcomes.

In the ensemble-based approach, variable importance was defined as the additional proportion of variability in the outcome explained by including an individual feature or group of features in the estimation procedure relative to the other features; this compares the conditional mean with all features included to the conditional mean omitting the individual or group of features of interest, but keeping all other features [50]. By definition, the true, population ensemble-based VIM is a number between zero and one. This VIM may also be viewed as a population difference in R^2^ values. This implies that negative ensemble-based VIM estimates are possible; negative estimates suggest that the features of interest decrease predictive performance. However, the magnitude of the estimate must be taken into account prior to drawing strong conclusions about decreases in prediction performance. We estimated a single VIM for each unique combination of feature, outcome, and dataset; the specific details of the estimation procedure are below.

The following procedure was applied to each of the two datasets separately. A cross-validated sequential prediction procedure was used in the ensemble-based approach to estimate the additional variability in the outcome explained by the features of interest. We used Super Learning [34] to perform predictions, with the same candidate learning algorithms [generalized linear models (including with interactions), stepwise regression, elastic net regression (lasso), random forest, boosted decision stumps, and generalized additive models] as in the Super Learner analyses for Objective 1 with a subset of the variable screens (all variables, and features selected with t-test univariate p-values). To illustrate the sequential prediction procedure, consider estimating the importance of the group of CD4bs (Group 2 above) in predicting the quantitative log IC_50_ outcome in dataset 1. First, we split dataset 1 into 10 folds. Using each fold in turn as a held-out test set, we: (1) fit a 10-fold cross-validated Super Learner to the remaining nine-tenths of the data, predicting quantitative log IC_50_ using all features; (2) fit a second 10-fold cross-validated Super Learner, using the same nine-tenths of the data, predicting the fitted values from the first Super Learner (as the outcome) using all features besides the CD4bs; (3) obtain predicted values on the held-out test set using the resulting algorithms from (1) and (2); and (4) use these predicted values, along with the test set outcome data, to compute an estimate of variable importance using the R package vimp [94], which implements the algorithm outlined in [50]. We then average these 10 VIMs, resulting in a 10-fold cross-validated estimate of the importance of the CD4bs; we also compute a 95% confidence interval for the true variable importance of the CD4bs, again using the vimp package. This process was repeated for each outcome of interest, each feature or group of features for which importance was desired, and both datasets. Finally, we estimate the average VIM for each feature or feature group by taking the mean across the two datasets; these within-dataset estimates are independent, and thus we can obtain a confidence interval for the average straightforwardly, again using the vimp package.

Since features are commonly selected for prediction because of their marginal gain in predictive ability in the context of all the other features, it is typical in a predictive model containing multiple features that several of the features will be working in a peripheral manner, and that they are only weakly correlated with the outcome in a univariate context. Such peripheral features would not be very useful in one type of sieve analysis: AA site scanning that tests sites one at a time for whether prevention efficacy depends on residue content at the site. Accordingly, we added a statistical test of each feature in a univariate context, using either logistic regression (for dichotomous outcomes) or linear regression (for quantitative outcomes), with geographic region of origin included in the model to control for any potential geographic confounding. Features with Wald test Holm-Bonferroni adjusted 2-sided p-value < 0.05 are flagged as having evidence for being associated with the outcome.

Final selection of top-ranked features for consideration in the primary sieve analysis of the AMP trials (i.e., to have satisfied Objective (2)) is based on both the algorithm-specific and ensemble approach VIMs, as well as on the Holm-Bonferroni adjusted 2-sided p-values noted above. In particular, we report as top-ranked features those with a Holm-Bonferroni adjusted 2-sided p-value < 0.05 and that rank among the top 50 features by either of the two VIM approaches. For the purpose of this paper, we focus on reporting and interpreting results for the outcomes with the largest sample size: IC_50_ censored (dichotomous) and quantitative log IC_50_ (continuous).

## Supporting information

S1 Fig Receiver operating characteristic curves for the best-performing cross-validated models in classifying the dichotomous sensitive/resistant outcome. Results are shown for the top three cross-validated models plus the cross-validated performance of the Super Learner, for A) dataset 1 and B) dataset 2. Values in parentheses are the cross-validated areas under the receiver operating characteristic curve (AUC) for the different models.
(PDF)

S2 Fig Classification boxplots for the best-performing models and the Super Learner in classifying the dichotomous sensitive/resistant only outcome. Cross-validated performance is shown for the Super Learner and for the top three individual models for (A) dataset 1 and (B) dataset 2.
(PDF)

S3 Fig CV-AUC point estimates and 95% confidence intervals for the Super Learner and all models trained to classify the IC_50_ censored outcome, for both data sets. A) Models trained on dataset 1. B) Models trained on dataset 2. Models using geography only are shown in red as a reference.
(PDF)

S4 Fig CV-AUC point estimates and 95% confidence intervals for the Super Learner and all other models trained to classify the dichotomous sensitive/resistant only outcome, for both data sets. A) Models trained on dataset 1. B) Models trained on dataset 2. Models using geography only are shown in red as a reference.
(PDF)

S5 Fig Cross-validated (A, C) and validated on the hold-out set (B, D) correlations for dataset 1 (A, B) and dataset 2 (C, D), for the model trained by the Super Learner to predict the quantitative log IC_50_ outcome. The corresponding point estimate of CV-R^2^ and its 95% CI (in parentheses) is shown in the lower right corner of each panel.
(PDF)

S6 Fig Cross-validated (A, C) and validated on the hold-out set (B, D) correlations for dataset 1 (A, B) and dataset 2 (C, D), for the model trained by the Super Learner to predict the quantitative log IC_80_ outcome. The corresponding point estimate of CV-R^2^ and its 95% CI (in parentheses) is shown in the lower right corner of each panel.
(PDF)

S7 Fig Cross-validated R^2^ point estimates and 95% confidence intervals for the Super Learner and all individual learners trained to predict the quantitative log IC_50_ outcome, on both data sets. A) Models trained on dataset 1. B) Models trained on dataset 2. Models using geography only are shown in red as a reference.
(PDF)

S8 Fig Cross-validated R^2^ point estimates and 95% confidence intervals for the Super Learner and all individual learners trained to predict the quantitative log IC^80^ outcome, on both data sets. A) Models trained on dataset 1. B) Models trained on dataset 2. Models using geography only are shown in red as a reference.
(PDF)

S9 Fig Cross-validated R^2^ point estimates and 95% confidence intervals for the Super Learner and all individual learners trained to predict the quantitative neutralization slope outcome, on both data sets. A) Models trained on dataset 1. B) Models trained on dataset 2. Models using geography only are shown in red as a reference.
(PDF)

S10 Fig Cross-validated (A, C) and validated on the hold-out set (B, D) correlations for dataset 1 (A, B) and dataset 2 (C, D), for the model trained by the Super Learner to predict the neutralization slope outcome (denoted Y on the y-axis). The corresponding point estimate of CV-R^2^ and its 95% CI (in parentheses) is shown in the lower right corner of each panel.
(PDF)

S11 Fig Ensemble-approach variable importance measures and 95% confidence intervals for the 13 feature groups for the 5 outcomes. Feature groups are ordered by their average predictive performance across both data sets. The 95% confidence intervals of the average performance is provided on the left of each panel.
(PDF)

S12 Fig The geometric means of the imputed log_10_ IC_50_ values for the pseudoviruses whose Env sequences were included in this analysis, presented by region and subtype. (PDF)

S1 Table The top ten performing models/algorithms and the Super Learner, trained to classify the IC_50_ censored outcome, for datasets 1 and 2. Point estimates of the area under the receiver operating characteristic curve (AUC) are included for cross-validated performance within each of the two datasets, and for validation on the other separate data set. 95% confidence intervals are provided in parentheses. The Super Learner algorithm coefficients are the weights assigned by the ensemble to individual learners.
(DOCX)

S2 Table The top ten performing models/algorithms and the Super Learner, trained to classify the dichotomous sensitive/resistant only outcome, for Dataset 1 and Dataset 2. Point estimates of the area under the receiver operating characteristic curve (AUC) are included for cross-validated performance within each of the two datasets, and for validation on the other separate data set. 95% confidence intervals are provided in parentheses. The Super Learner algorithm coefficients are the weights assigned by the ensemble to individual learners.
(DOCX)

S3 Table The top ten performing models/algorithms and the Super Learner, trained to predict the quantitative log IC_50_ outcome, for dataset 1 and dataset 2. Point estimates of the area under the receiver operating characteristic curve (AUC) are included for cross-validated performance within each of the two datasets, and for validation on the other separate data set. 95% confidence intervals are provided in parentheses. The Super Learner algorithm coefficients are the weights assigned by the ensemble to individual learners.
(DOCX)

S4 Table The top ten performing models/algorithms and the Super Learner, trained to predict the quantitative log IC^80^ outcome, for dataset 1 and dataset 2. Point estimates of the area under the receiver operating characteristic curve (AUC) are included for cross-validated performance within each of the two datasets, and for validation on the other separate data set. 95% confidence intervals are provided in parentheses. The Super Learner algorithm coefficients are the weights assigned by the ensemble to individual learners.
(DOCX)

S5 Table The top ten performing models/algorithms and the Super Learner, trained to predict the neutralization slope outcome, for dataset 1 and dataset 2. Point estimates of the area under the receiver operating characteristic curve (AUC) are included for cross-validated performance within each of the two datasets, and for validation on the other separate data set. 95% confidence intervals are provided in parentheses. The Super Learner algorithm coefficients are the weights assigned by the ensemble to individual learners.
(DOCX)

S6 Table The 80 variables contributing to classifying the IC_50_ censored outcome by the *lasso* (using geography and all features pre-selected by lasso) method and their estimated coefficients. Features with a positive coefficient are associated with neutralization resistance, while features with a negative coefficient are associated with neutralization sensitivity. This table of coefficients can be used to build a linear model for classifying neutralization resistance from the content of a given HIV-1 envelope sequence.
(DOCX)

S7 Table Variable importance measure (VIM) information for the features that have a Holm-Bonferroni p-value less than 0.05, ranked by their contribution to the prediction of the (A) sensitive/resistant only outcome, (B) quantitative log IC_80_ outcome, or (C) neutralization slope outcome.
(DOCX)

S8 Table Numbers of HIV-1 Envelope sequences in the CATNAP database by subtype/recombinant subtype, country of origin, and geographic region of origin, broken down by dataset 1, dataset 2, and all sequences (datasets 1+2 combined). (DOCX)

S1 Text Text describing the feature selection process and providing additional details related to the analysis. (DOCX)
