# Supplementary material for: Prediction of VRC01 neutralization sensitivity by HIV-1 gp160 sequence features
Source: PLoS Comput Biol. 2019 Apr 1;15(4):e1006952. doi: 10.1371/journal.pcbi.1006952 (PMC6459550; doi:10.1371/journal.pcbi.1006952)
Supplement: S1 Fig — Results are shown for the top three cross-validated models plus the cross-validated performance of the Super Learner, for A) dataset 1 and B) dataset 2. Values in parentheses are the cross-validated areas under the receiver operating characteristic curve (AUC) for the different models. (PDF) [file pcbi.1006952.s001.pdf]

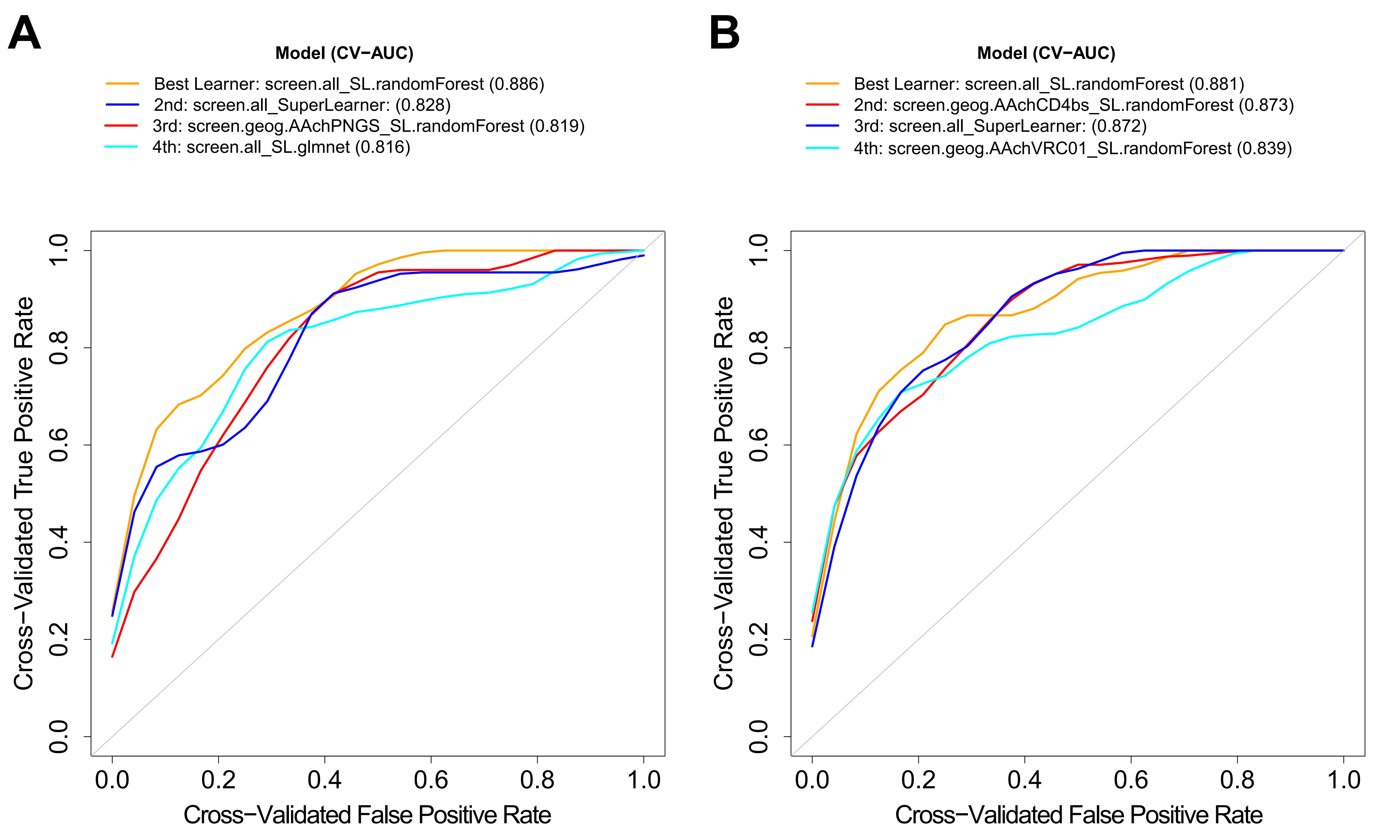
A
Model (CV−AUC)
Best Learner: screen.all_SL.randomForest (0.886)
2nd: screen.all_SuperLearner: (0.828)
3rd: screen.geog.AAchPNGS_SL.randomForest (0.819)
4th: screen.all_SL.glmnet (0.816)
Cross−Validated True Positive Rate
Cross−Validated False Positive Rate
0.0
0.2
0.4
0.6
0.8
1.0
B
Model (CV−AUC)
Best Learner: screen.all_SL.randomForest (0.881)
2nd: screen.geog.AAchCD4bs_SL.randomForest (0.873)
3rd: screen.all_SuperLearner: (0.872)
4th: screen.geog.AAchVRC01_SL.randomForest (0.839)
Cross−Validated True Positive Rate
Cross−Validated False Positive Rate
0.0
0.2
0.4
0.6
0.8
1.0
