# Supplementary figures and images for: Prediction of VRC01 neutralization sensitivity by HIV-1 gp160 sequence features
Source: PLoS Comput Biol. 2019 Apr 1;15(4):e1006952. doi: 10.1371/journal.pcbi.1006952 (PMC6459550; doi:10.1371/journal.pcbi.1006952)

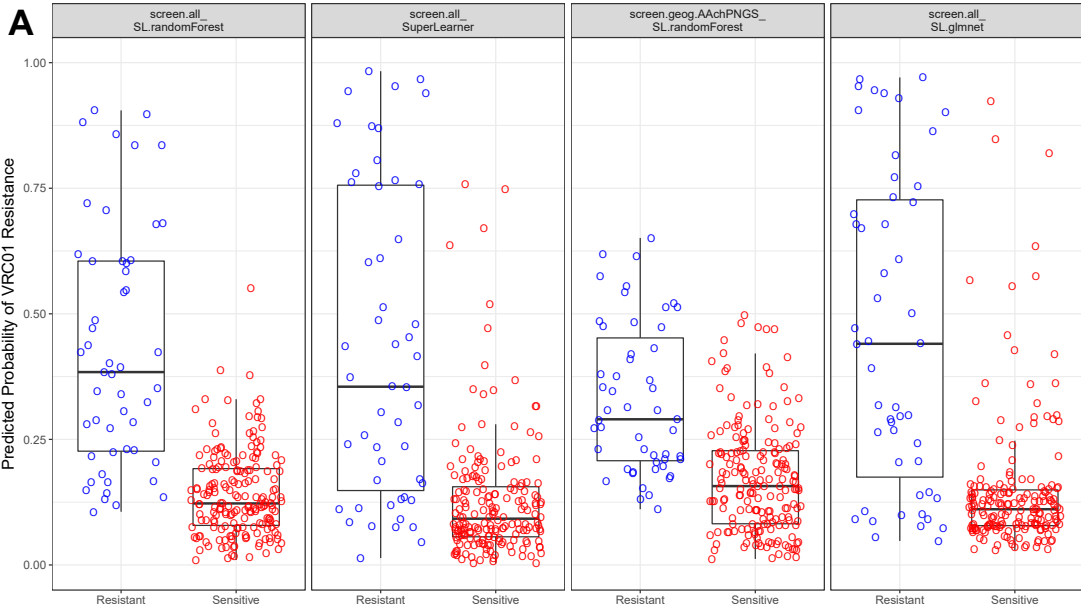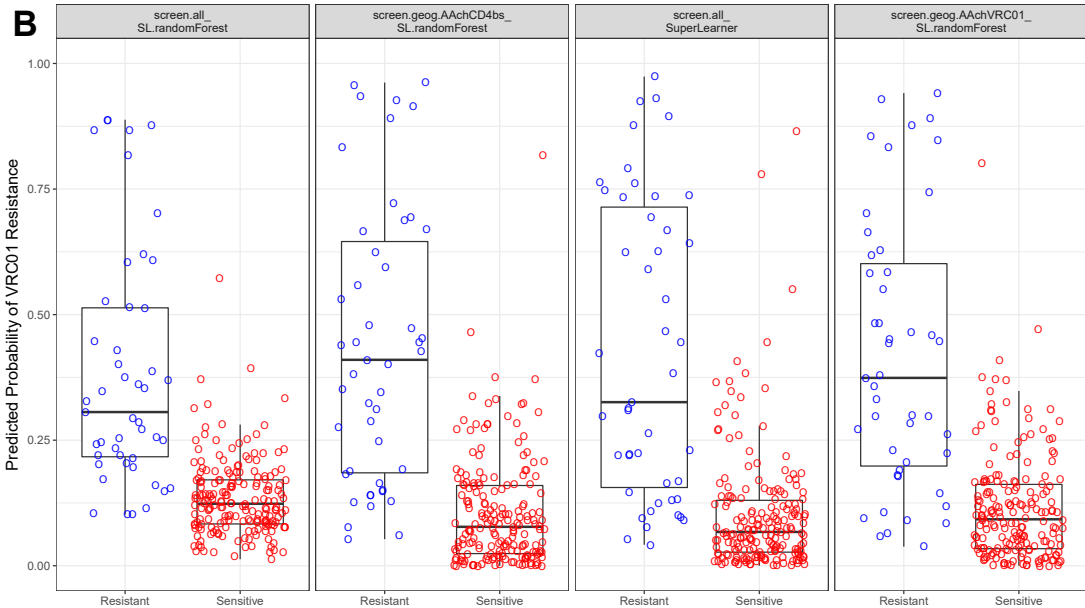

Supplement: S2 Fig — Cross-validated performance is shown for the Super Learner and for the top three individual models for (A) dataset 1 and (B) dataset 2. (PDF) [file pcbi.1006952.s002.pdf]

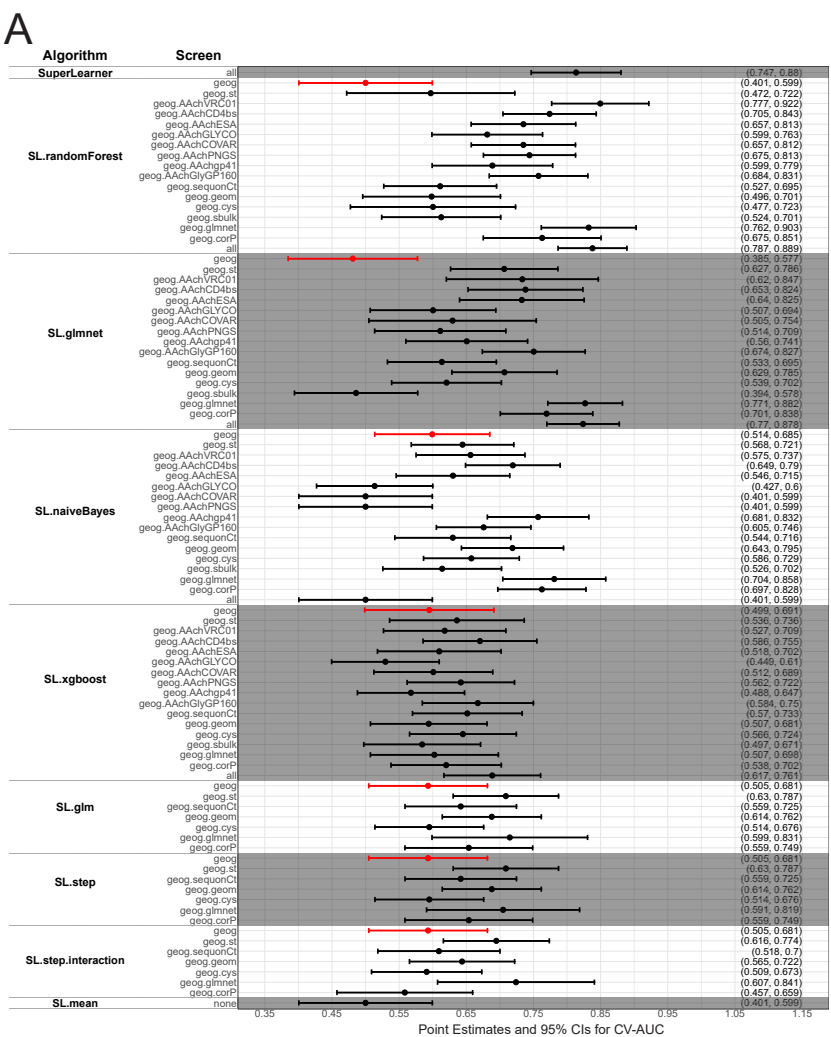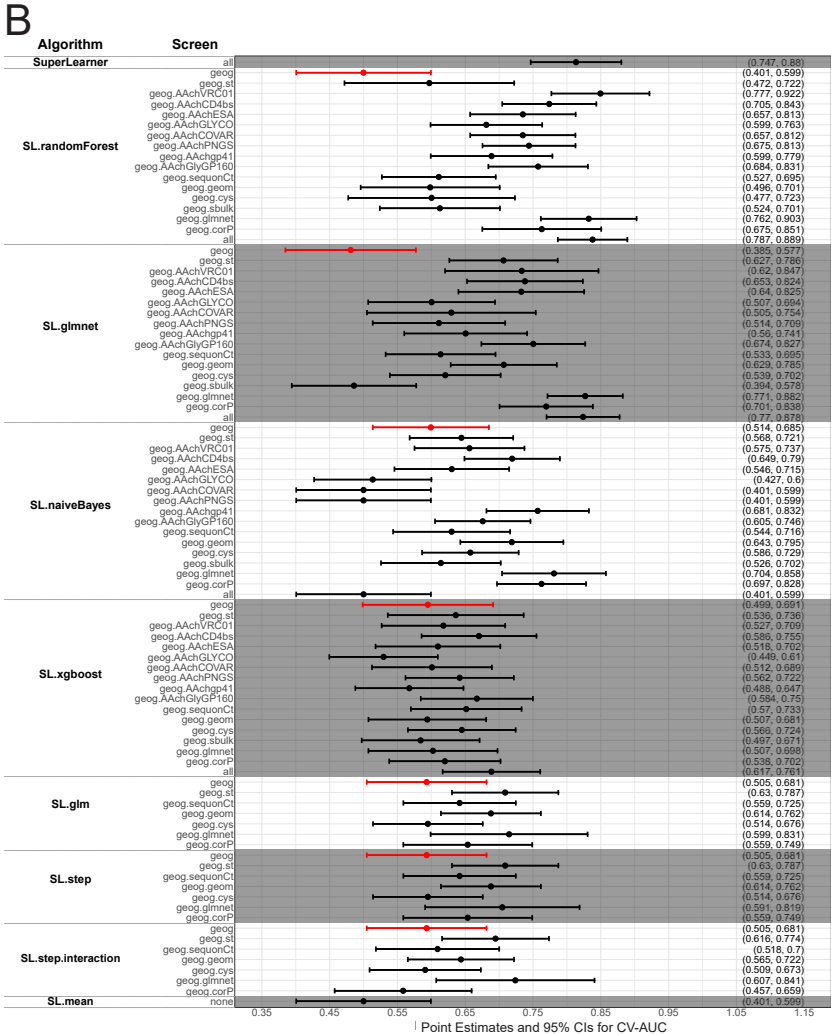

Supplement: S3 Fig — A) Models trained on dataset 1. B) Models trained on dataset 2. Models using geography only are shown in red as a reference. (PDF) [file pcbi.1006952.s003.pdf]

B

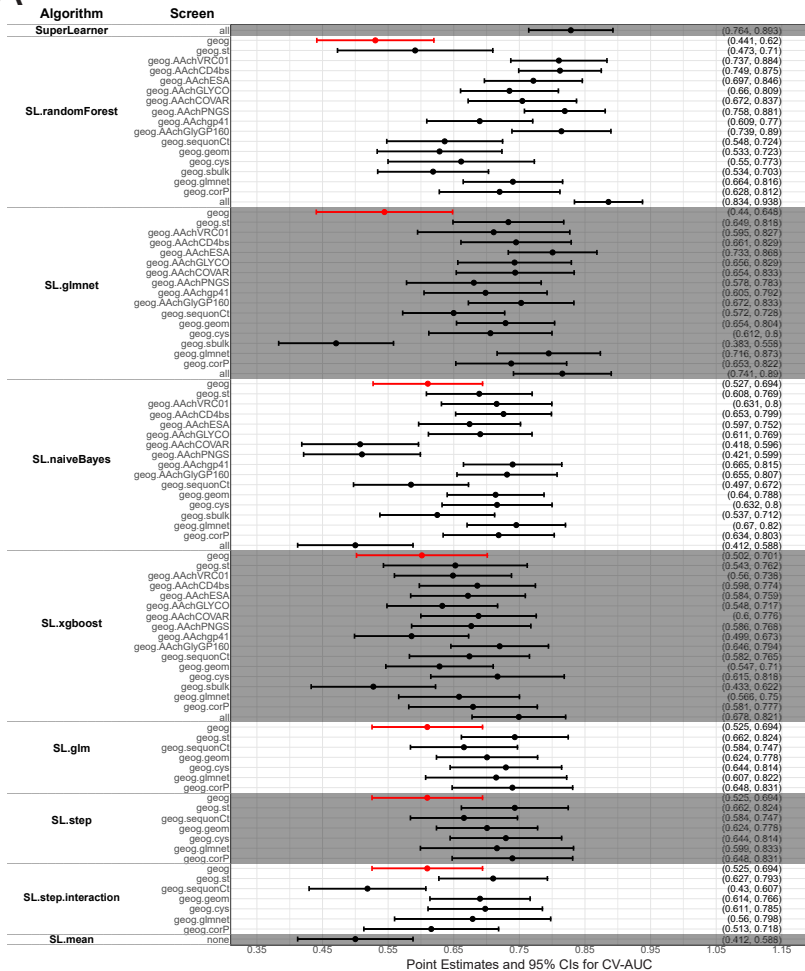

B

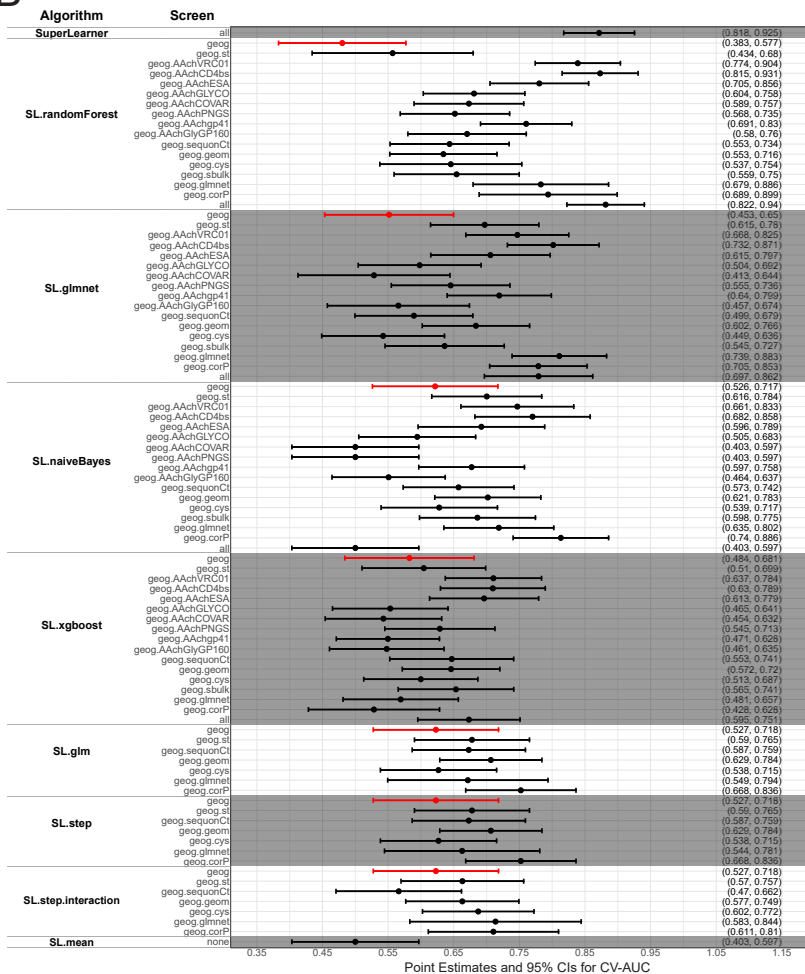

Supplement: S4 Fig — A) Models trained on dataset 1. B) Models trained on dataset 2. Models using geography only are shown in red as a reference. (PDF) [file pcbi.1006952.s004.pdf]

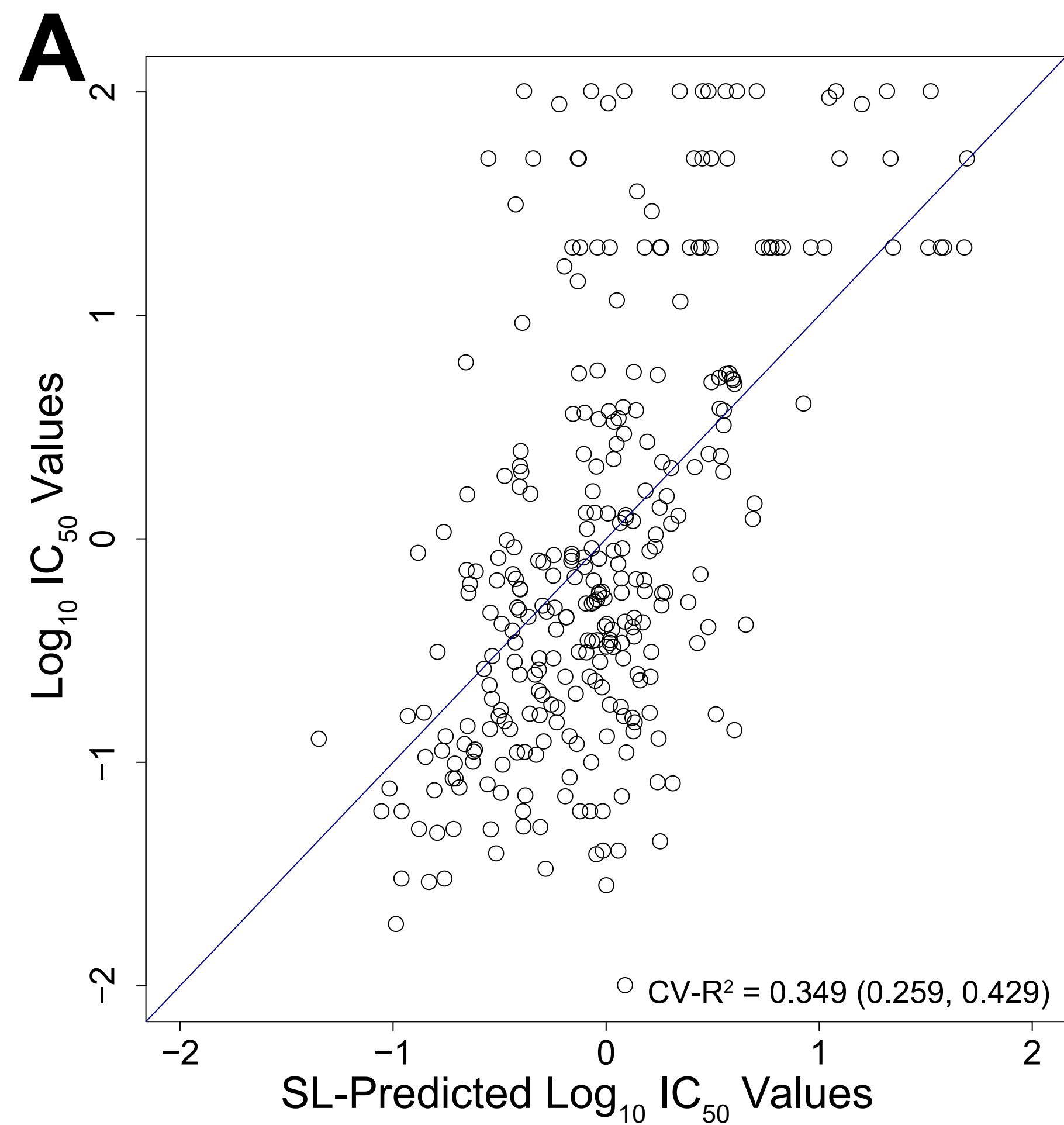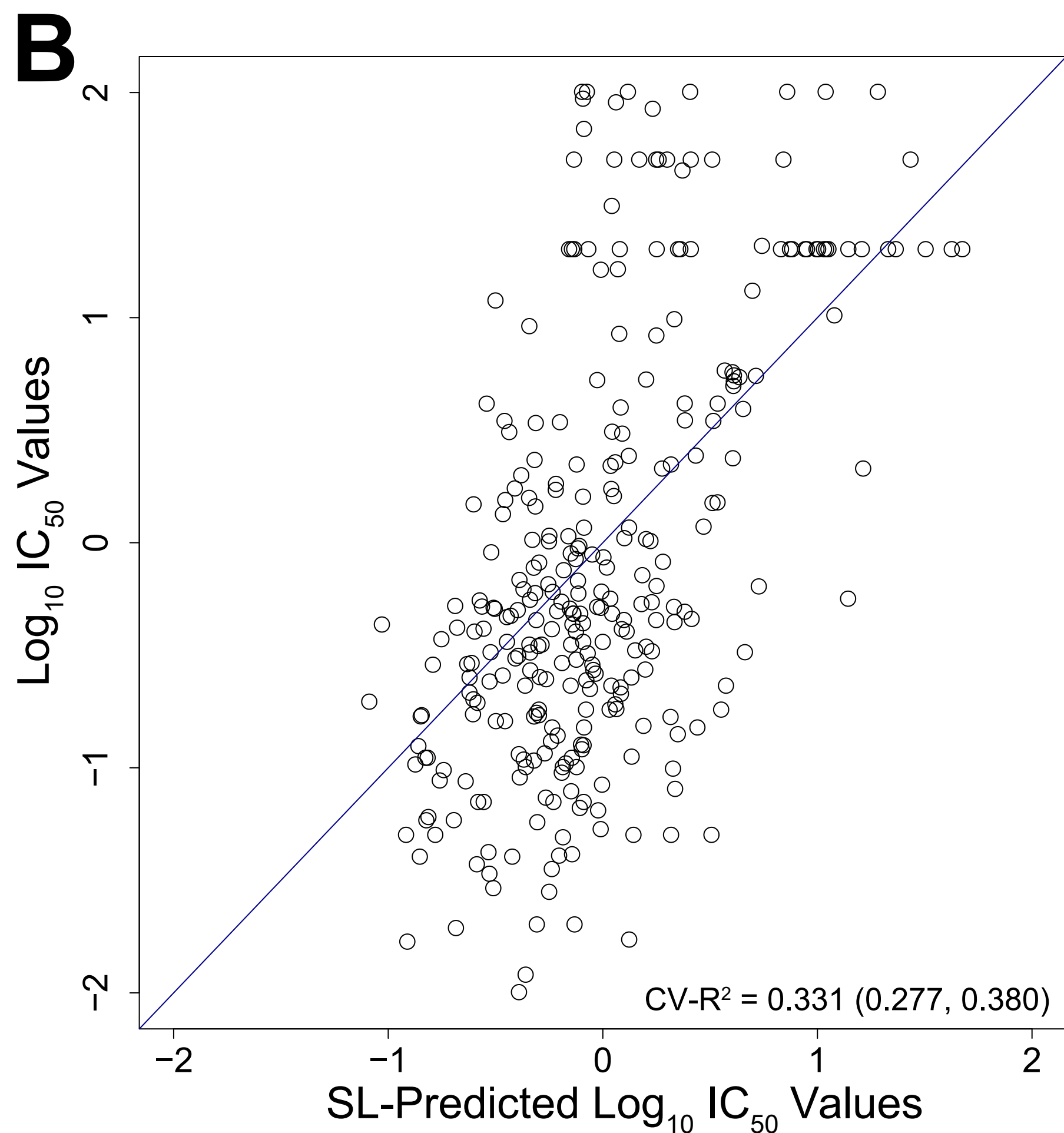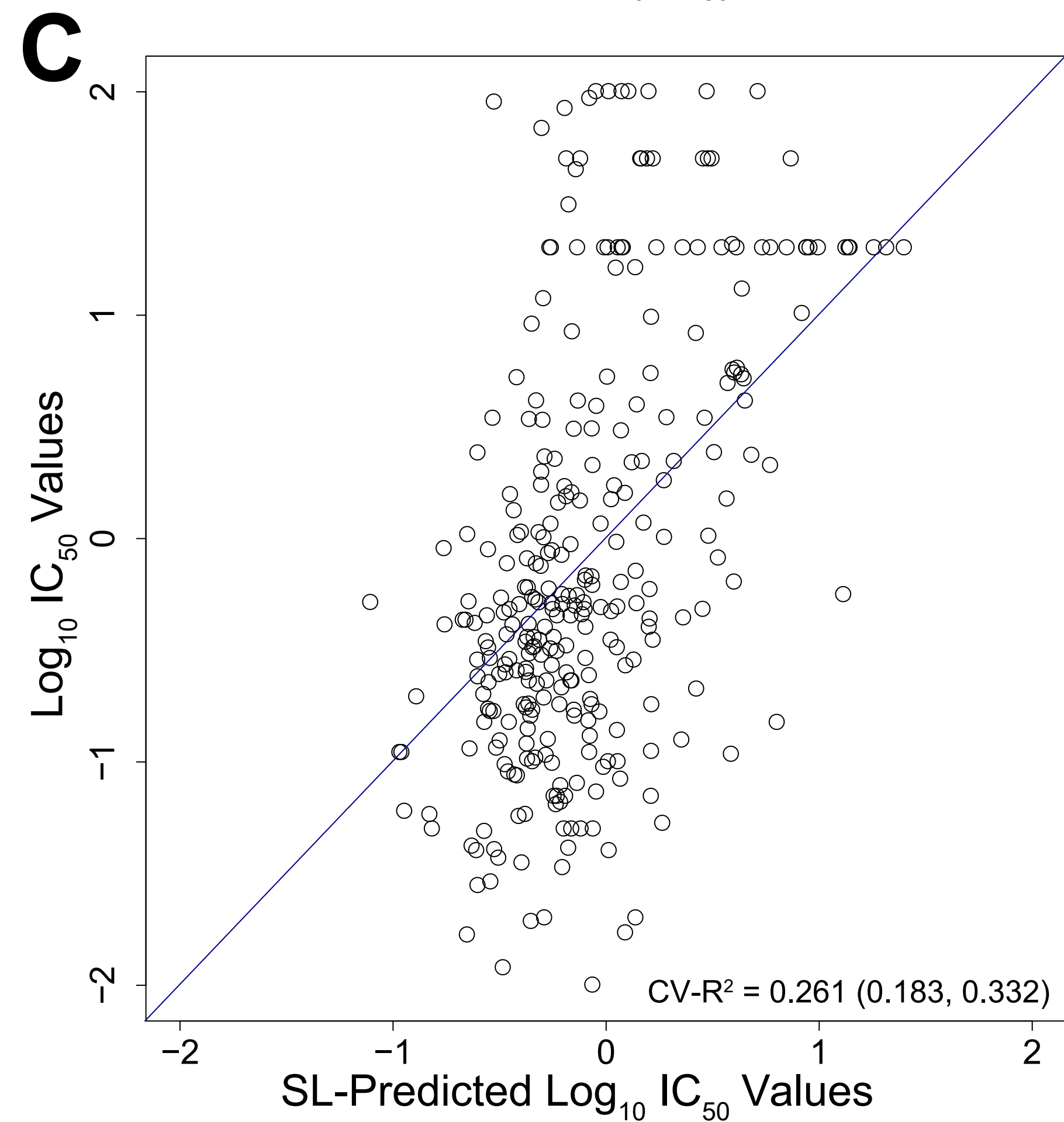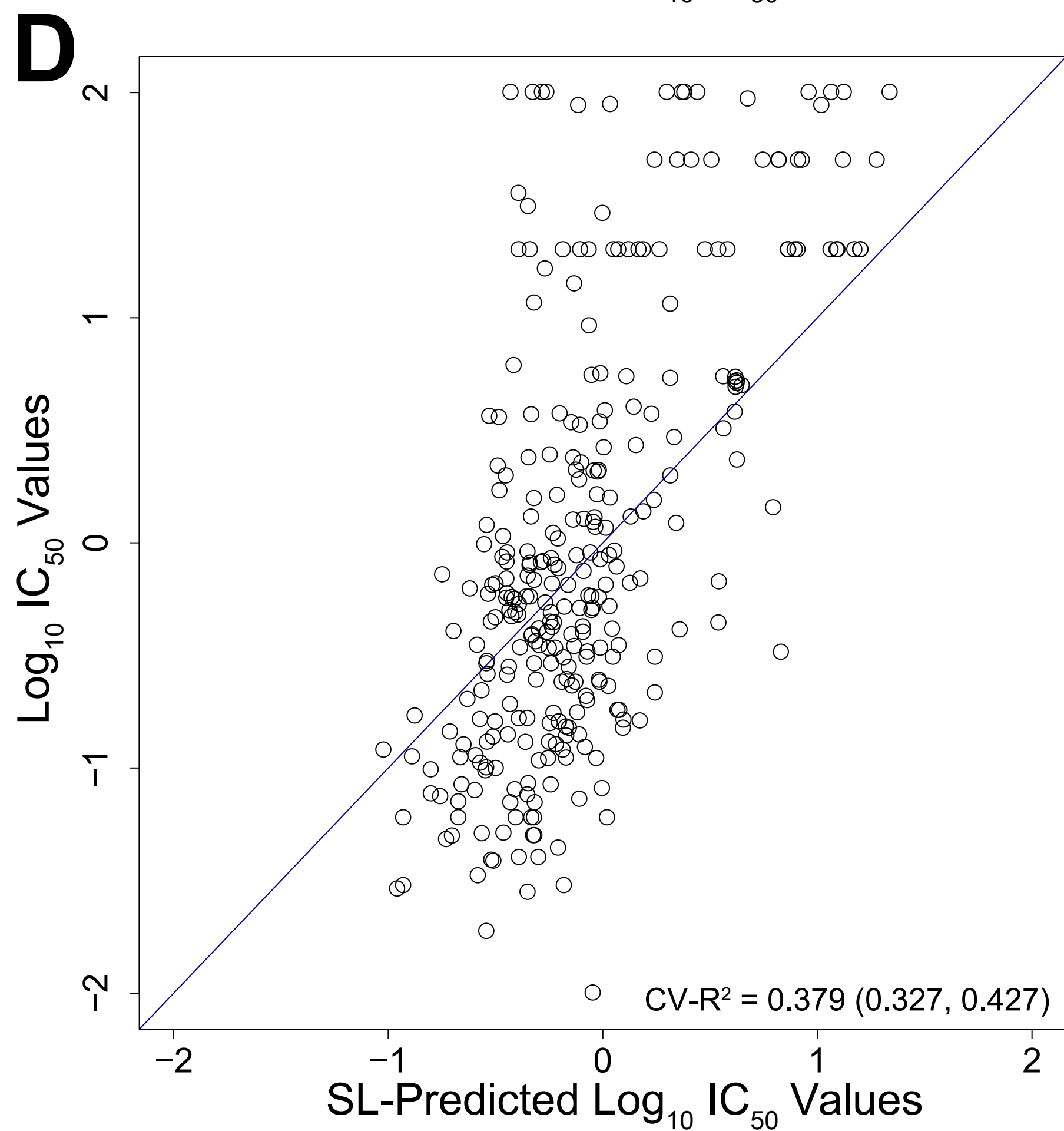

Supplement: S5 Fig — Cross-validated (A, C) and validated on the hold-out set (B, D) correlations for dataset 1 (A, B) and dataset 2 (C, D), for the model trained by the Super Learner to predict the quantitative log IC50 outcome. The corresponding point estimate of CV-R2 and its 95% CI (in parentheses) is shown in the lower right corner of each panel. (PDF) [file pcbi.1006952.s005.pdf]

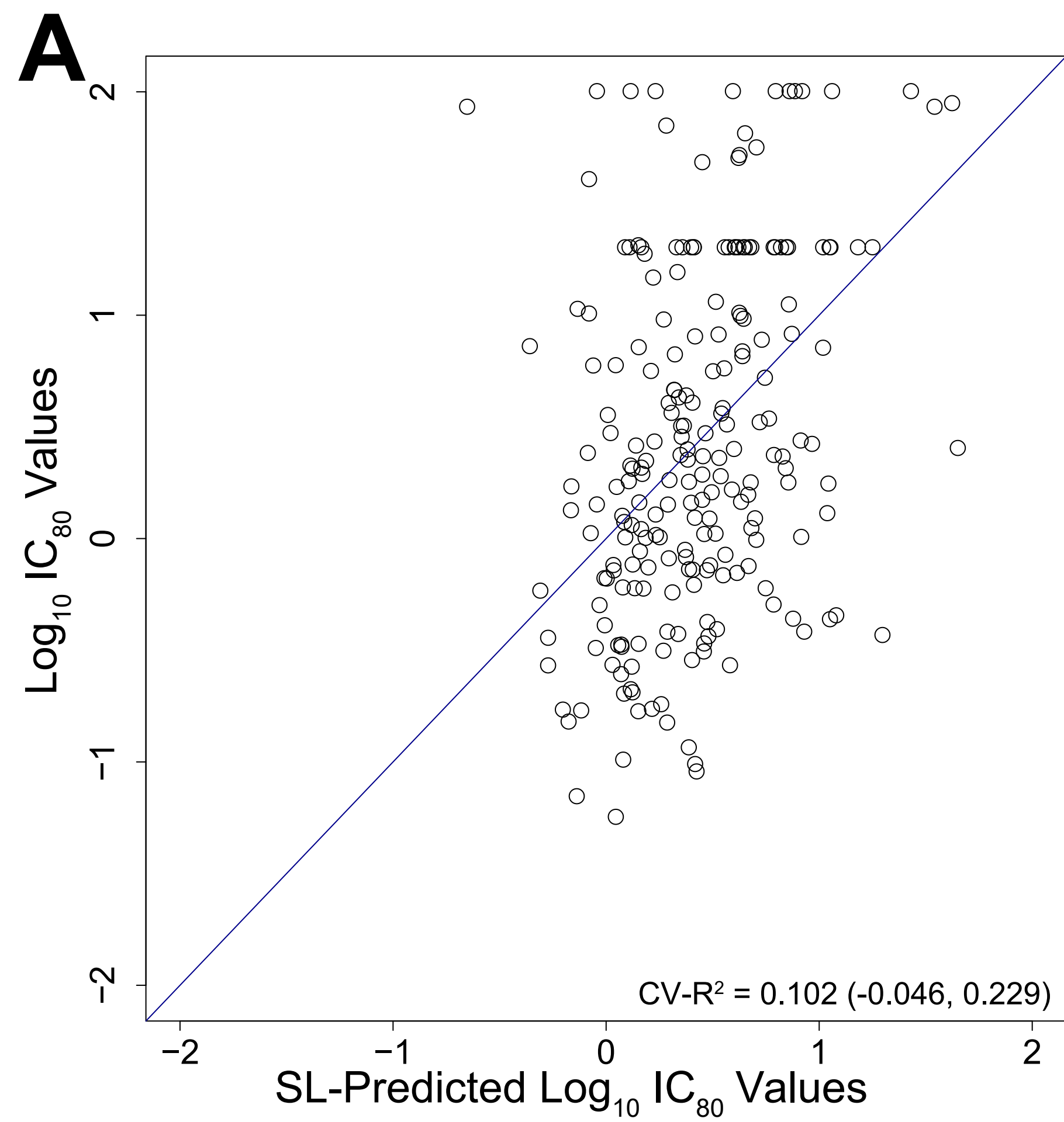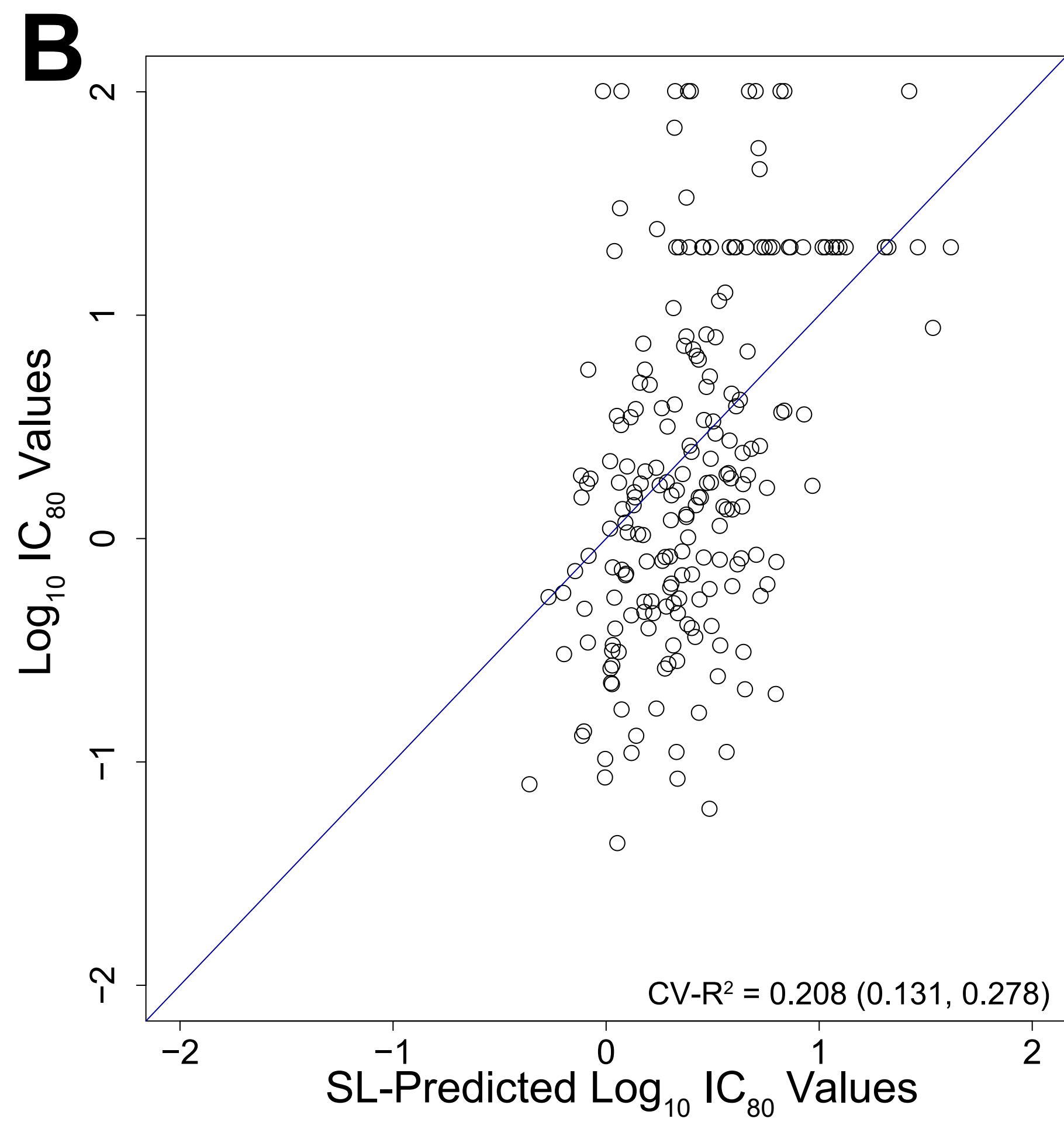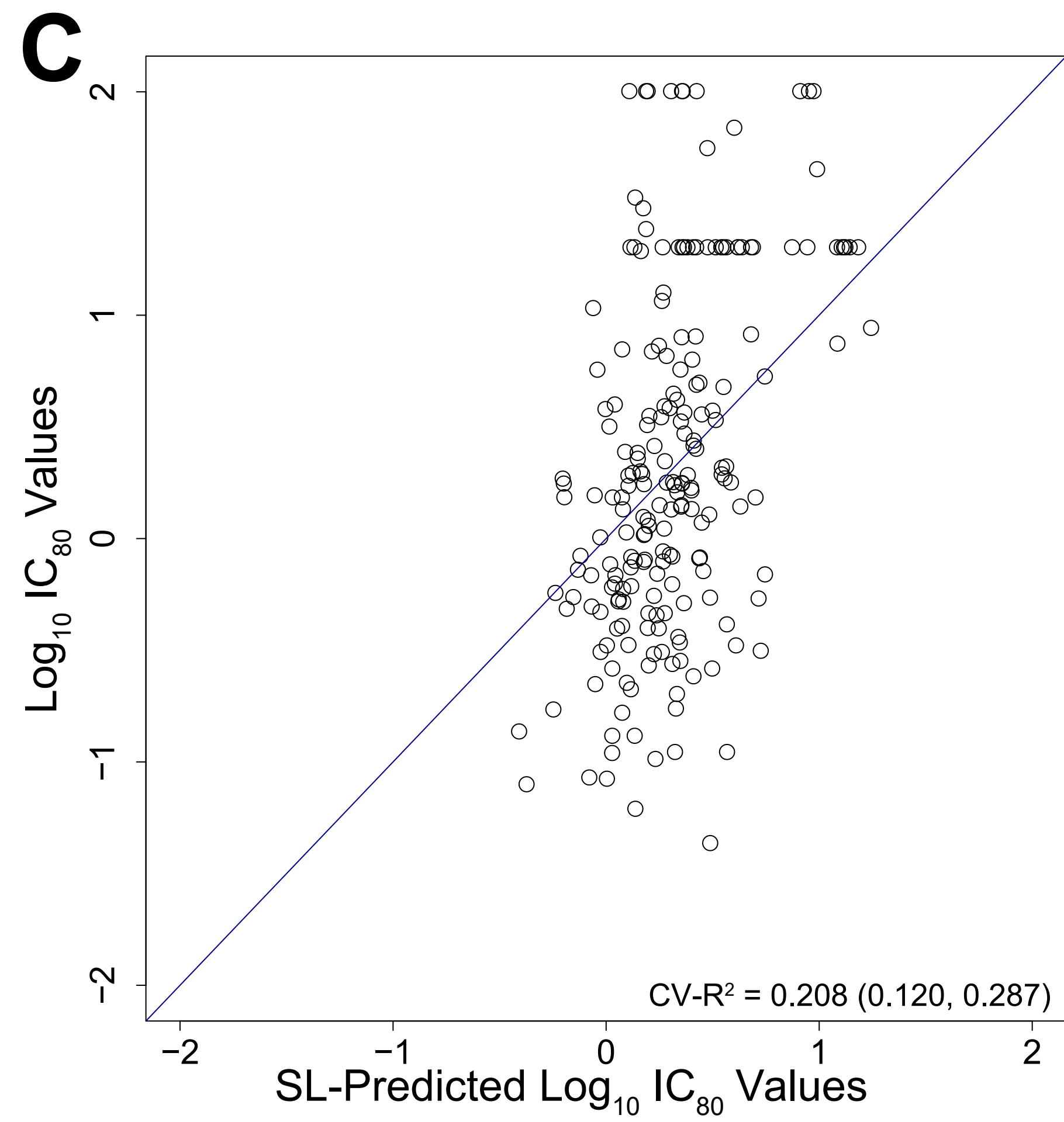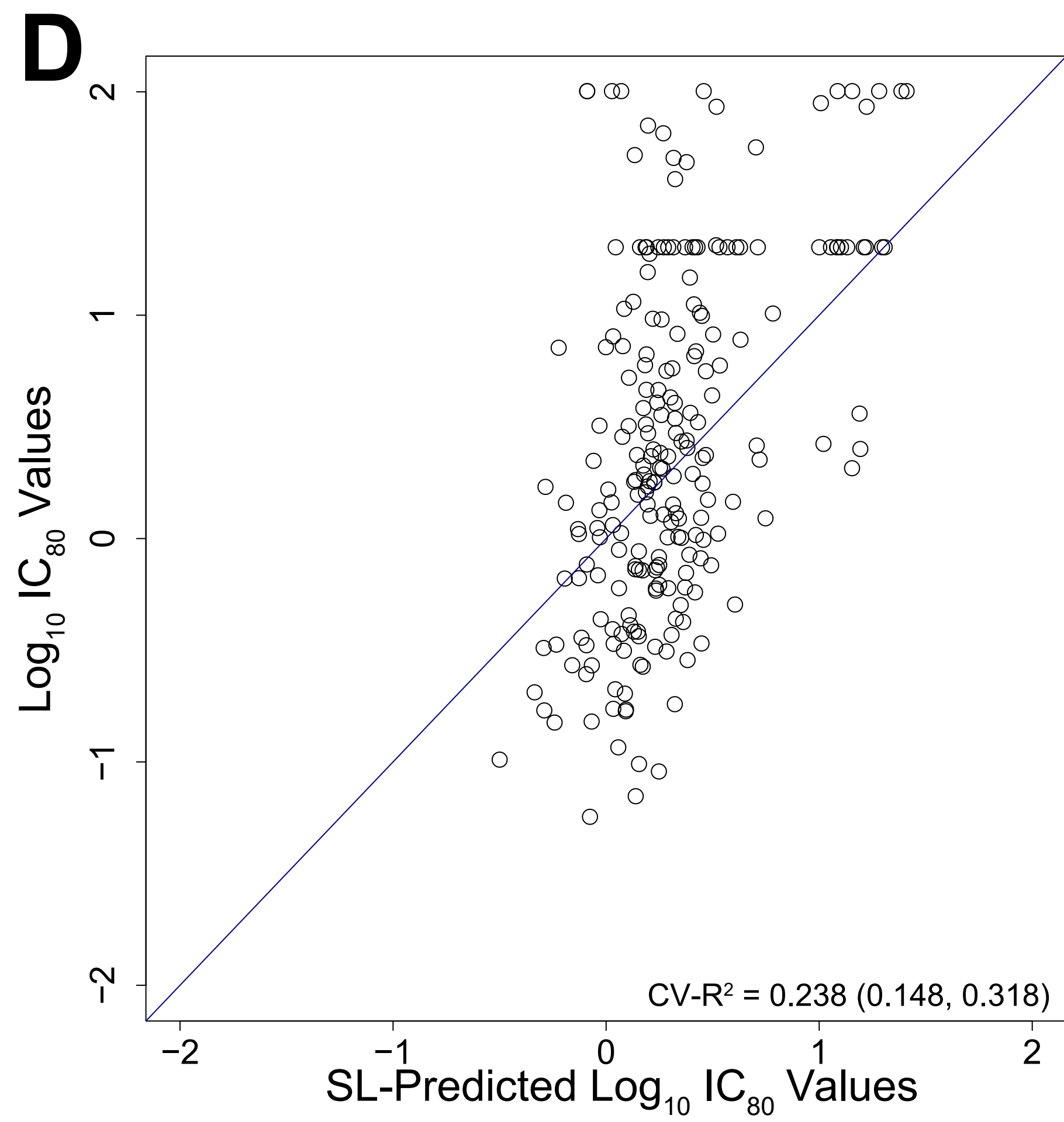

Supplement: S6 Fig — Cross-validated (A, C) and validated on the hold-out set (B, D) correlations for dataset 1 (A, B) and dataset 2 (C, D), for the model trained by the Super Learner to predict the quantitative log IC80 outcome. The corresponding point estimate of CV-R2 and its 95% CI (in parentheses) is shown in the lower right corner of each panel. (PDF) [file pcbi.1006952.s006.pdf]

A

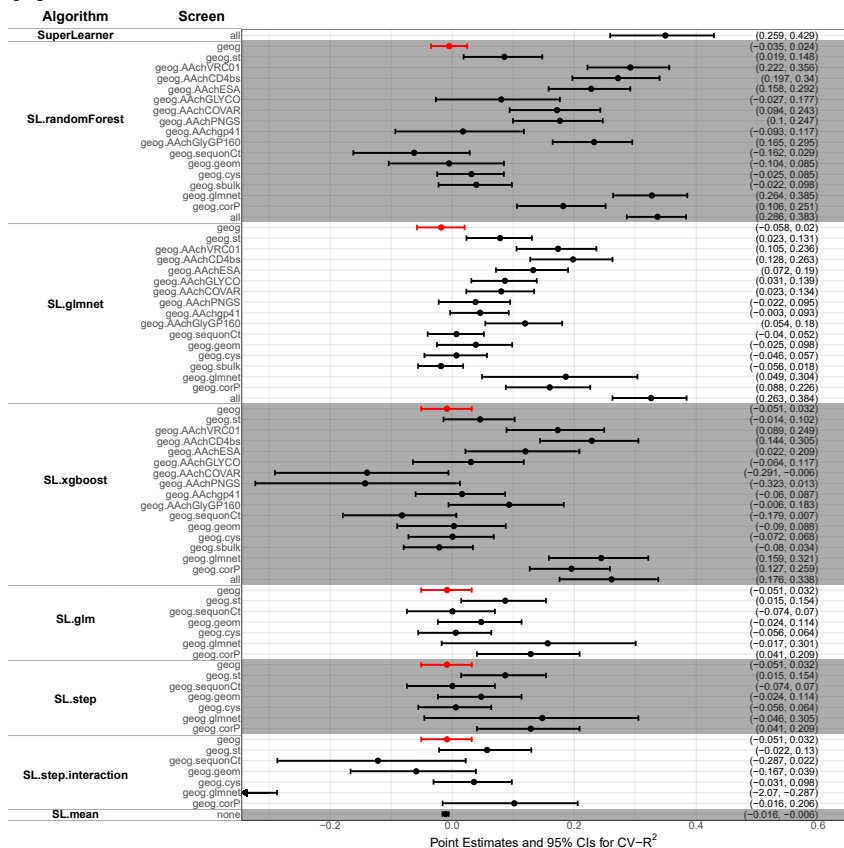

B

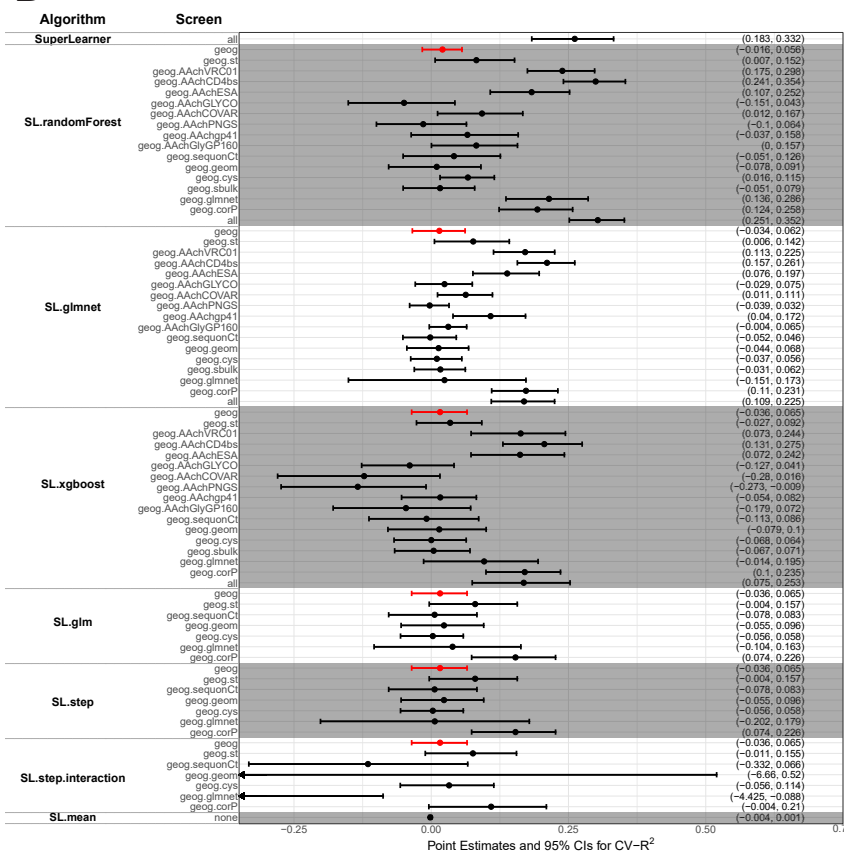

Supplement: S7 Fig — A) Models trained on dataset 1. B) Models trained on dataset 2. Models using geography only are shown in red as a reference. (PDF) [file pcbi.1006952.s007.pdf]

A

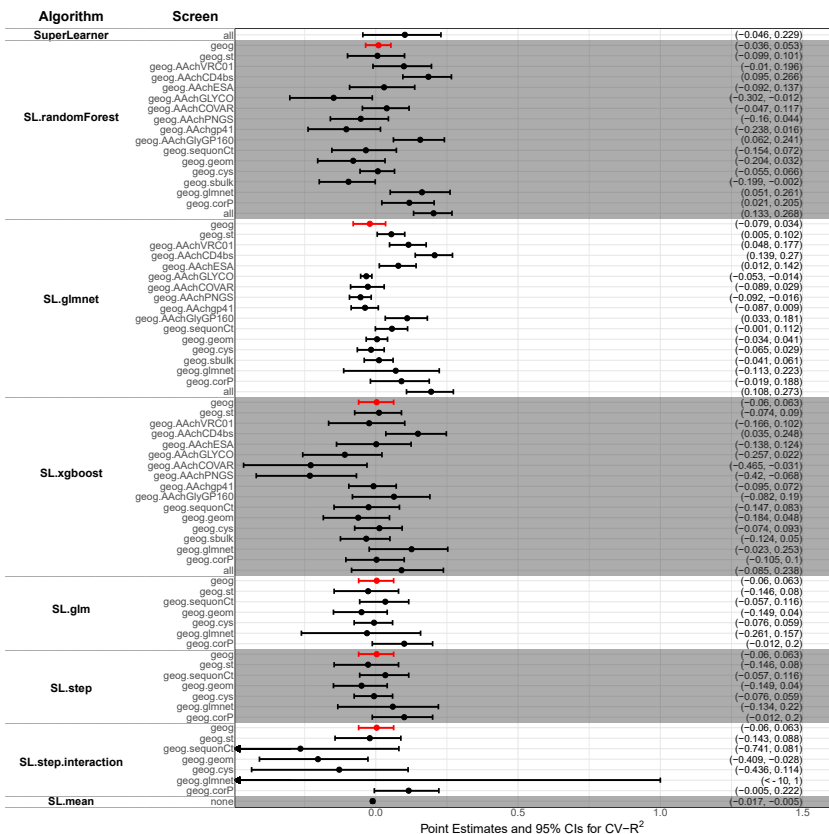Point Estimates and 95% CIs for CV-R<sup>2</sup>

B

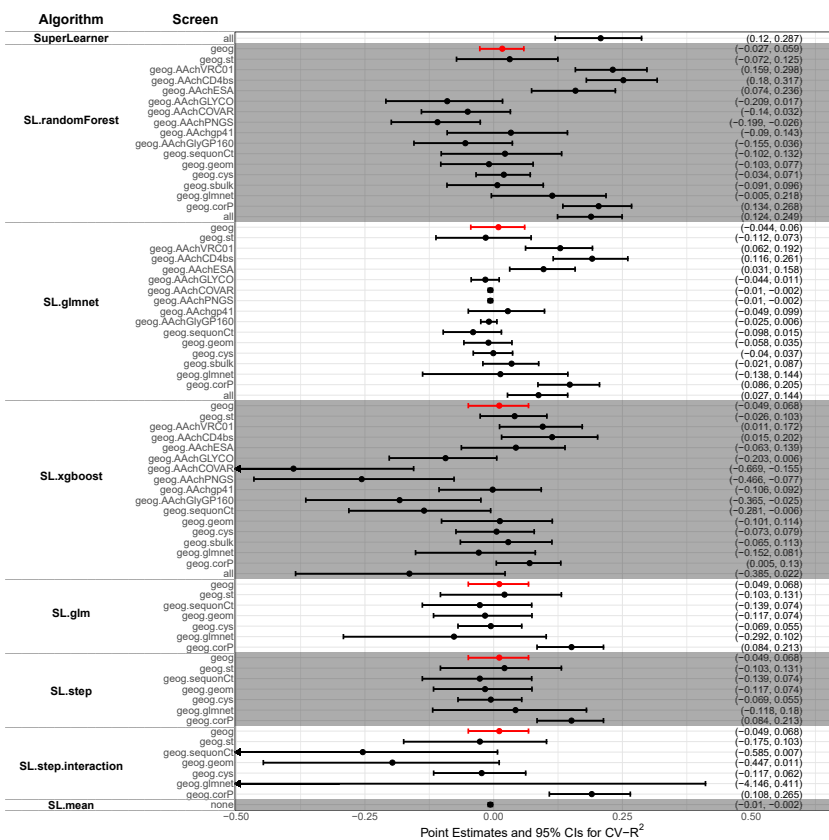Point Estimates and 95% CIs for CV-R<sup>2</sup>

Supplement: S8 Fig — A) Models trained on dataset 1. B) Models trained on dataset 2. Models using geography only are shown in red as a reference. (PDF) [file pcbi.1006952.s008.pdf]

A

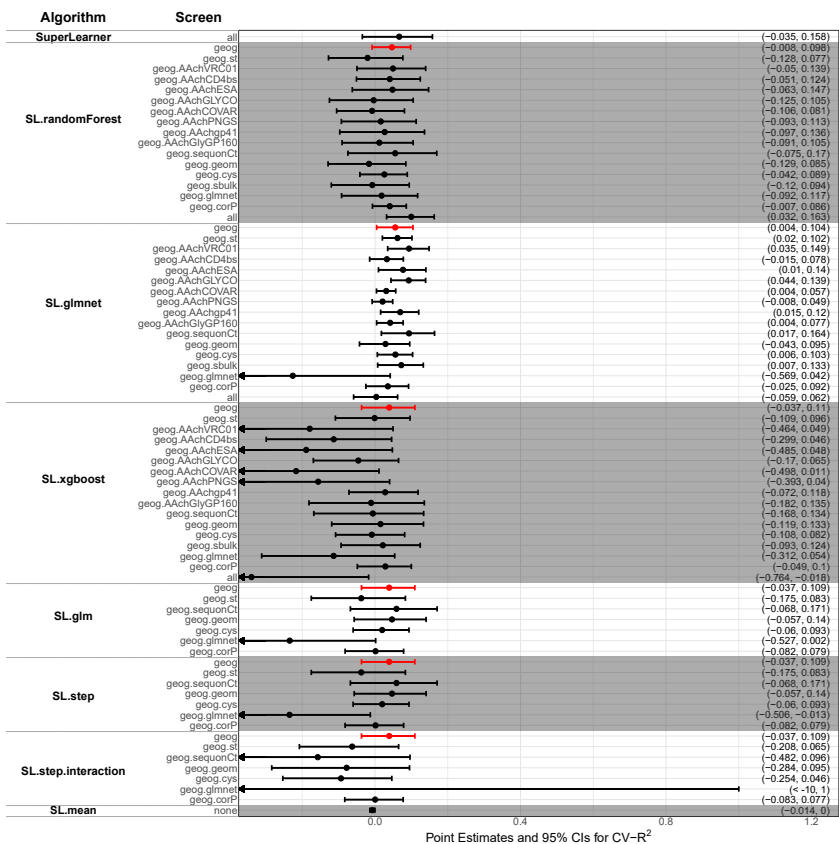

B

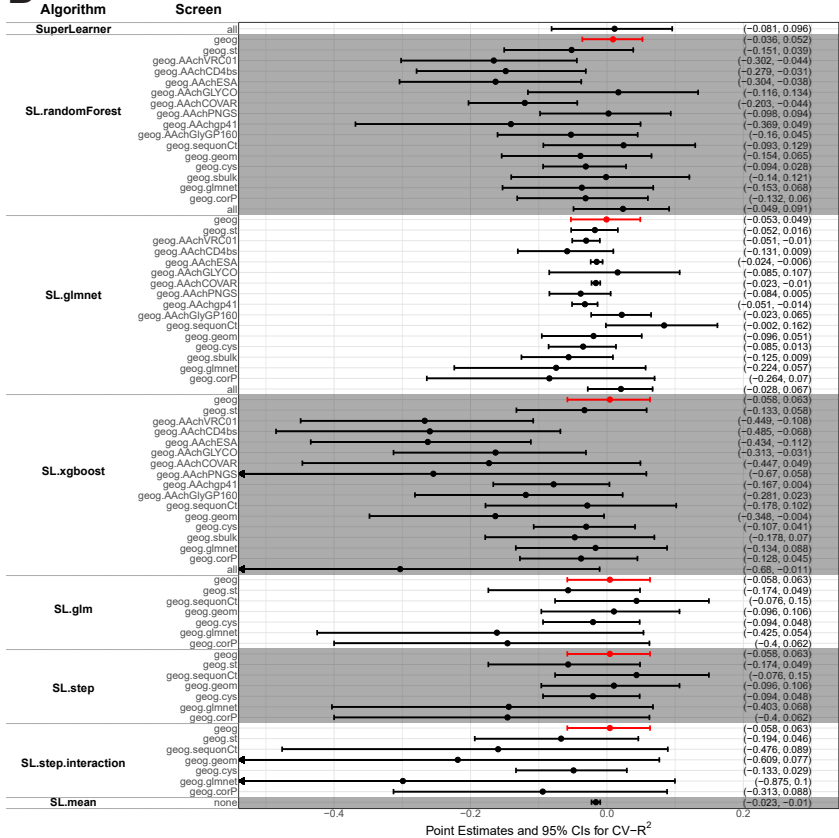

Supplement: S9 Fig — A) Models trained on dataset 1. B) Models trained on dataset 2. Models using geography only are shown in red as a reference. (PDF) [file pcbi.1006952.s009.pdf]

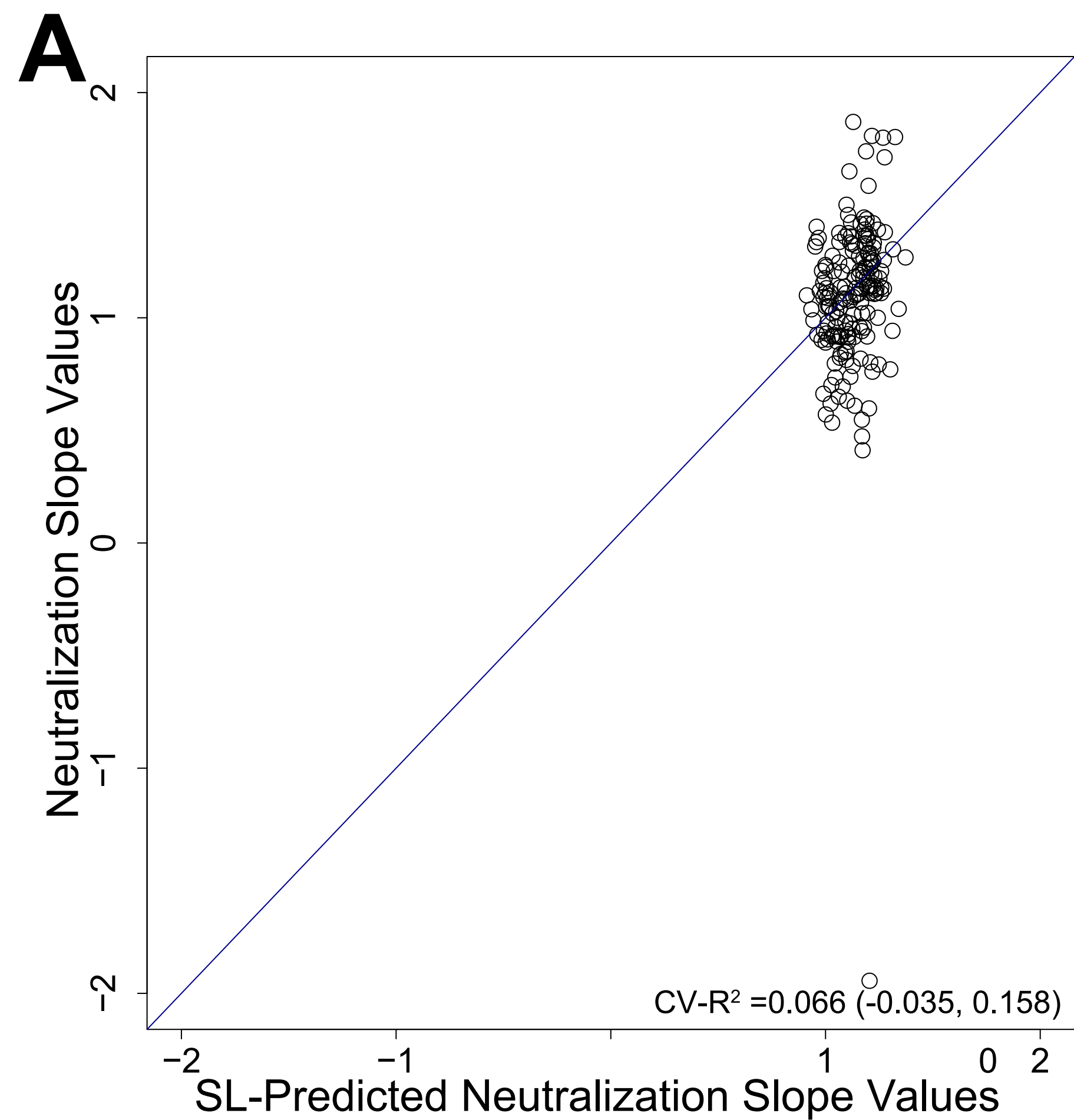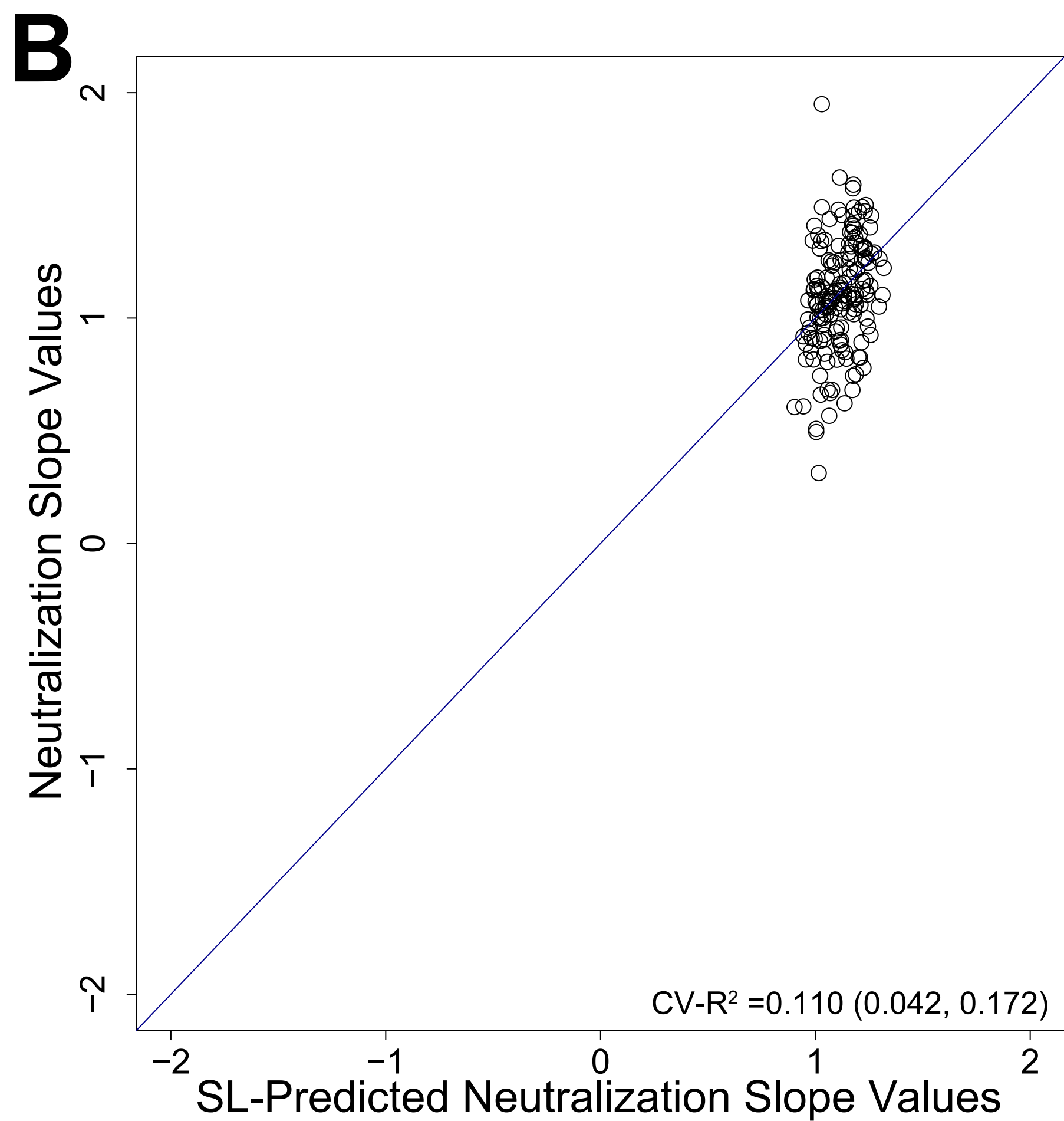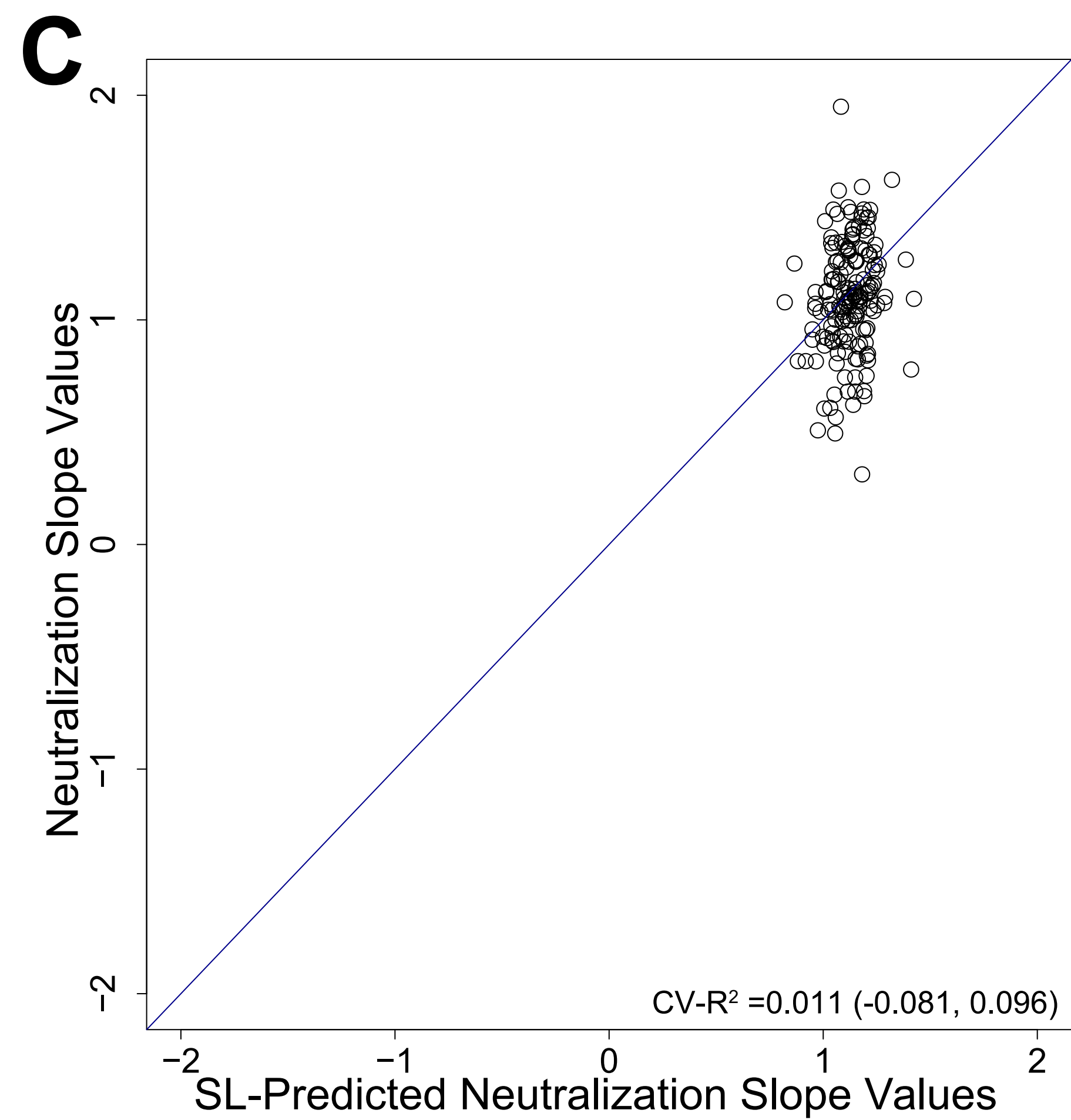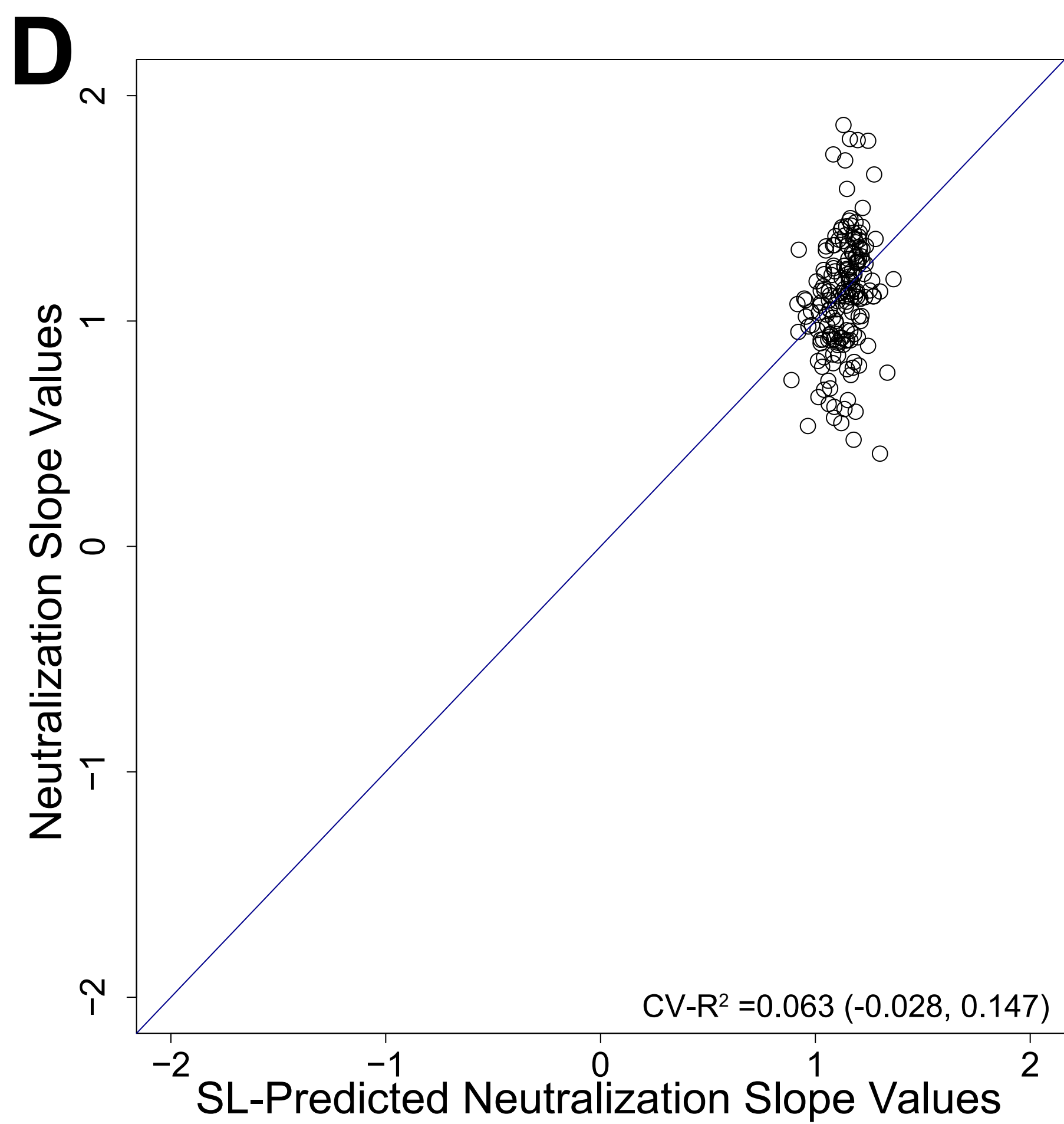

Supplement: S10 Fig — Cross-validated (A, C) and validated on the hold-out set (B, D) correlations for dataset 1 (A, B) and dataset 2 (C, D), for the model trained by the Super Learner to predict the neutralization slope outcome (denoted Y on the y-axis). The corresponding point estimate of CV-R2 and its 95% CI (in parentheses) is shown in the lower right corner of each panel. (PDF) [file pcbi.1006952.s010.pdf]

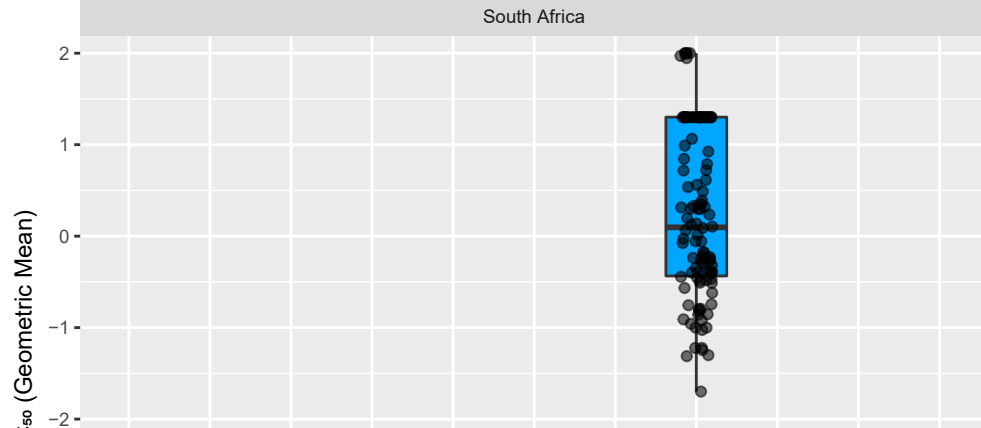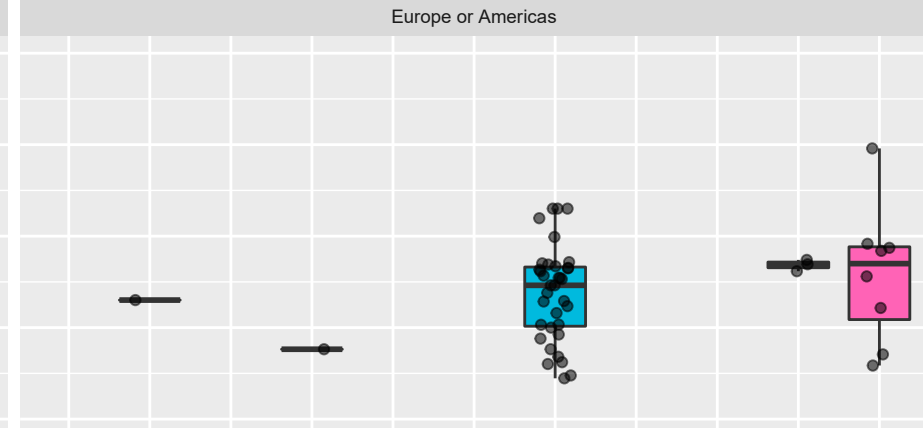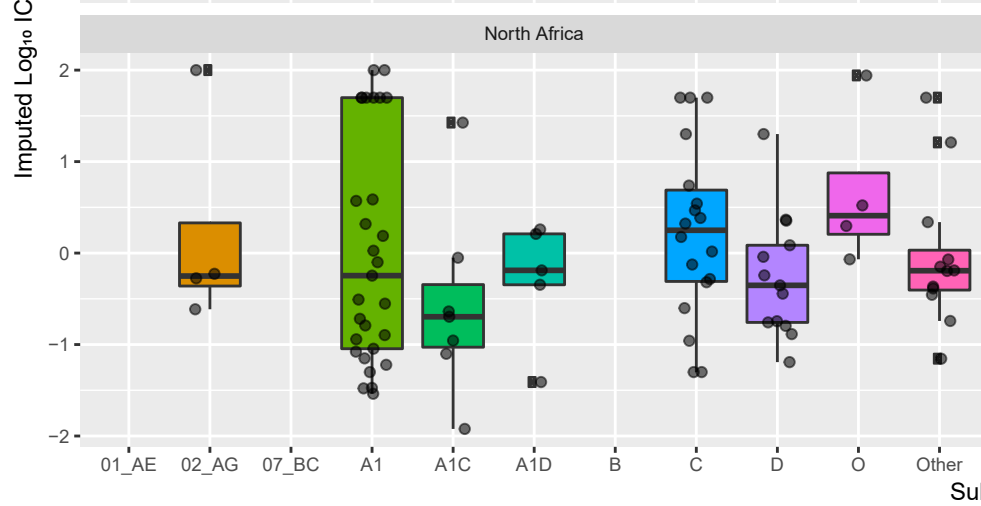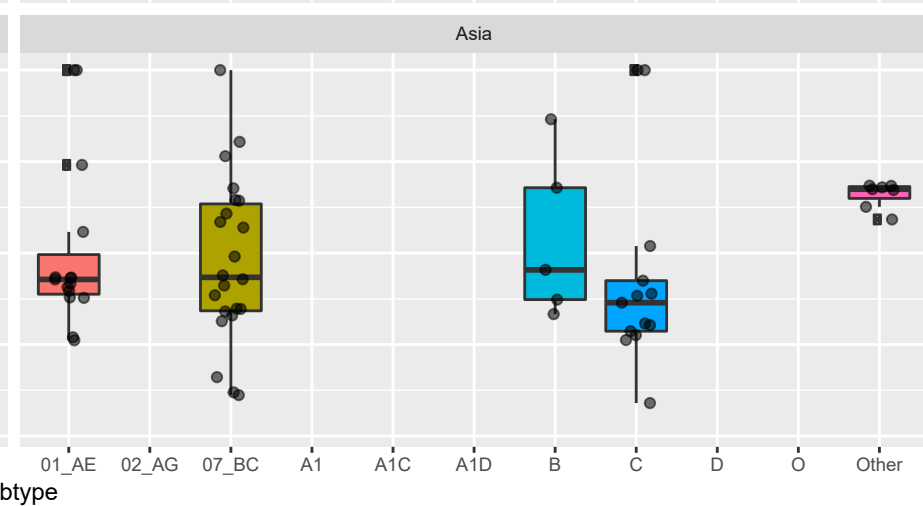

Supplement: S12 Fig — (PDF) [file pcbi.1006952.s012.pdf]
