# Supplementary material for: Prediction of VRC01 neutralization sensitivity by HIV-1 gp160 sequence features
Source: PLoS Comput Biol. 2019 Apr 1;15(4):e1006952. doi: 10.1371/journal.pcbi.1006952 (PMC6459550; doi:10.1371/journal.pcbi.1006952)
Supplement: S11 Fig — Feature groups are ordered by their average predictive performance across both data sets. The 95% confidence intervals of the average performance is provided on the left of each panel. (PDF) [file pcbi.1006952.s011.pdf]

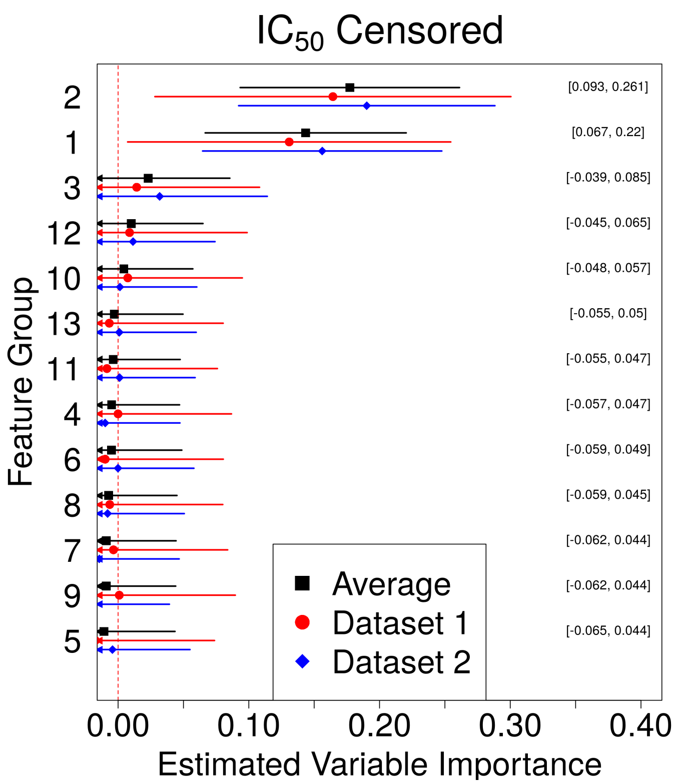

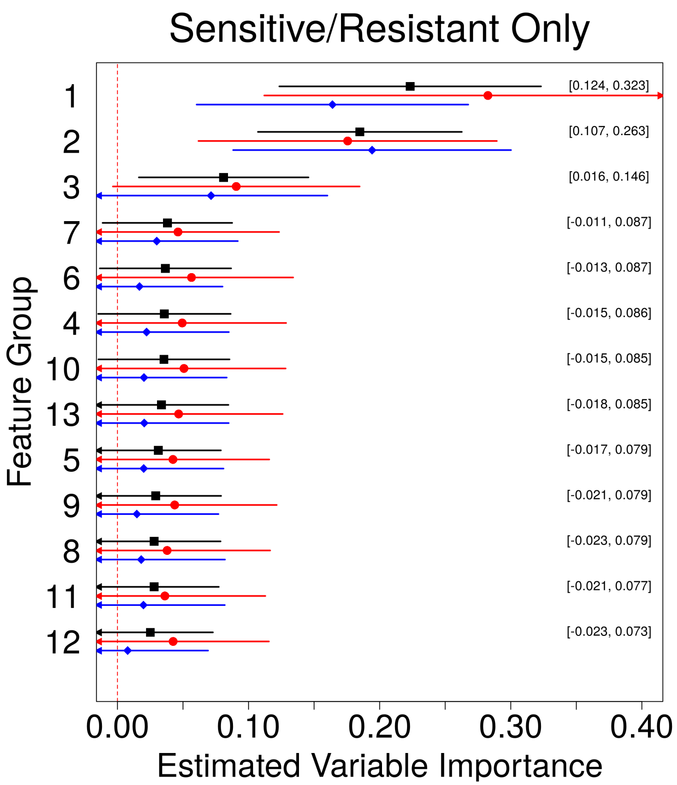

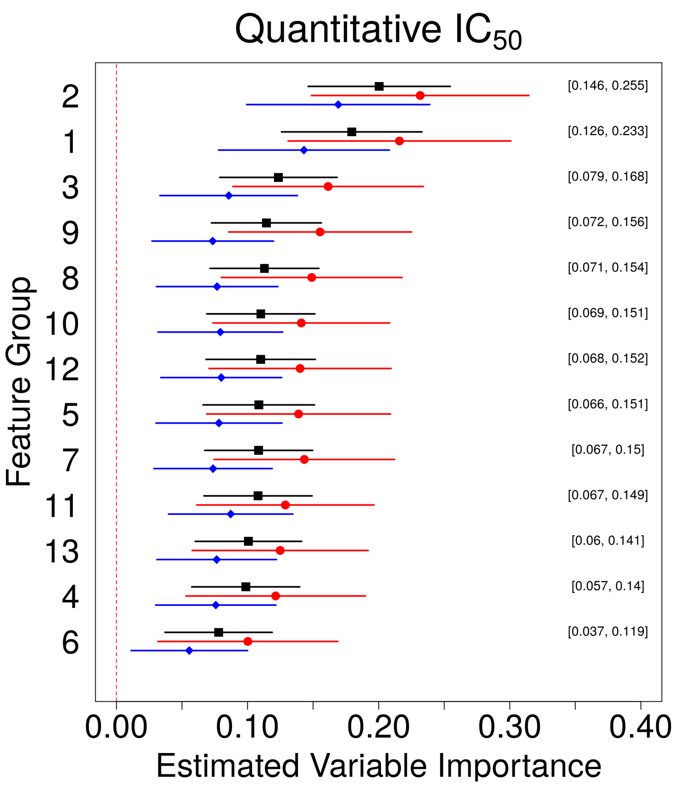

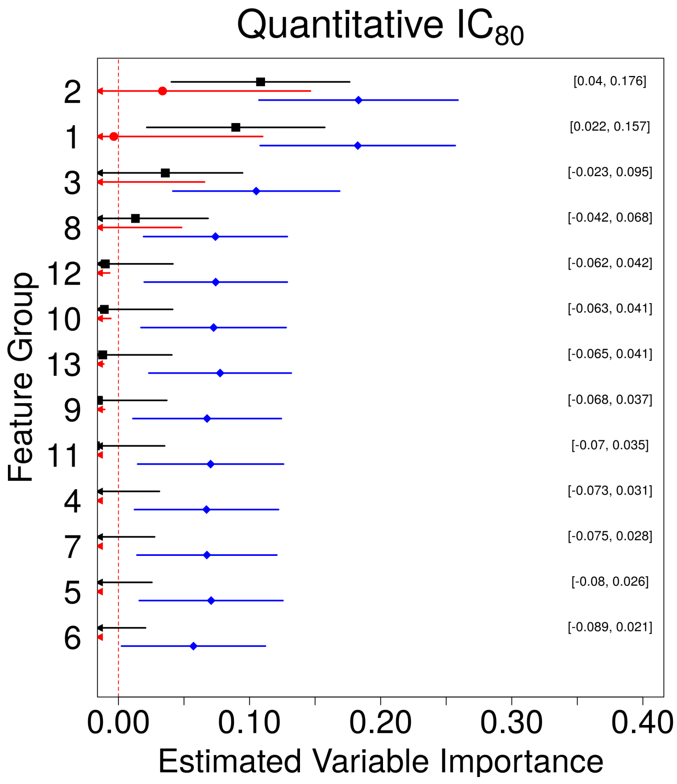

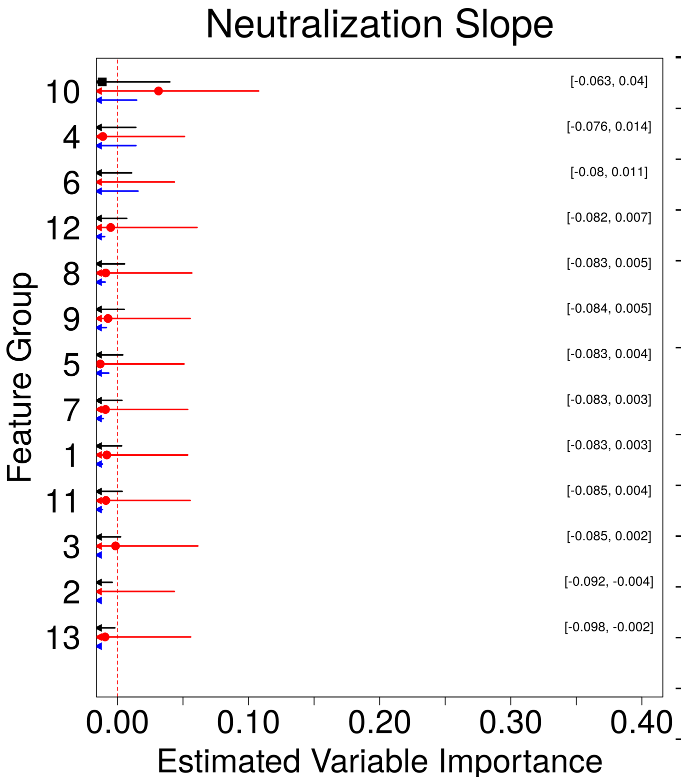

Feature group definitions

1	VRC01 binding footprint
2	CD4 binding sites
3	Sites with sufficient exposed surface area
4	Sites identified as important for glycosylation
5	Sites with residues that covary with the VRC01 binding footprint
6	Sites associated with VRC01-specific potential N-linked glycosylation (PNGS) effects
7	gp41 sites important for VRC01 binding
8	Sites for indicating N-linked glycosylation
9	Majority virus subtypes
10	Region-specific counts of PNGS
11	Viral geometry
12	Cysteine counts
13	Steric bulk at critical locations
