# Supplementary material for: Prediction of VRC01 neutralization sensitivity by HIV-1 gp160 sequence features
Source: PLoS Comput Biol. 2019 Apr 1;15(4):e1006952. doi: 10.1371/journal.pcbi.1006952 (PMC6459550; doi:10.1371/journal.pcbi.1006952)
Supplement: S1 Table — Point estimates of the area under the receiver operating characteristic curve (AUC) are included for cross-validated performance within each of the two datasets, and for validation on the other separate data set. 95% confidence intervals are provided in parentheses. The Super Learner algorithm coefficients are the weights assigned by the ensemble to individual learners. (DOCX) [file pcbi.1006952.s013.docx]

S1 Table. The top ten performing models/algorithms and Super Learner, trained to predict the IC_50_ censored outcome, for dataset 1 and dataset 2. Point estimates of the area under the receiver operating characteristic curve (AUC) are included for cross-validated performance within each of the two datasets, and for validation on the other separate data set. 95% confidence intervals are provided in parentheses. The Super Learner algorithm coefficients are the weights assigned by the ensemble to individual learners.

|  | Screen | Algorithm | AUC (cross validation) (CI) | | AUC (validated on dataset 2) (CI) |
| --- | --- | --- | --- | --- | --- |
| Dataset 1 | geog.AAchVRC01 | SL.randomForest | 0.849 (0.777, 0.922) | | 0.877 (0.819, 0.934) |
|  | all | SL.randomForest | 0.838 (0.787, 0.889) | | 0.914 (0.870, 0.958) |
|  | geog.glmnet | SL.randomForest | 0.832 (0.762, 0.903) | | 0.863 (0.786, 0.939) |
|  | geog.glmnet | SL.glmnet | 0.827 (0.771, 0.882) | | 0.837 (0.769, 0.906) |
|  | all | SL.glmnet | 0.824 (0.770, 0.878) | | 0.858 (0.792, 0.924) |
|  | all | SuperLearner | 0.813 (0.747, 0.880) | | 0.884 (0.836, 0.932) |
|  | geog.glmnet | SL.naiveBayes | 0.781 (0.704, 0.858) | | 0.805 (0.739, 0.872) |
|  | geog.AAchCD4bs | SL.randomForest | 0.774 (0.705, 0.843) | | 0.895 (0.846, 0.944) |
|  | geog.corP | SL.glmnet | 0.769 (0.701, 0.838) | | 0.871 (0.807, 0.936) |
|  | geog.corP | SL.randomForest | 0.763 (0.675, 0.851) | | 0.860 (0.773, 0.948) |
|  | geog.corP | SL.naiveBayes | 0.763 (0.697, 0.828) | | 0.842 (0.779, 0.905) |
|  | geog.AAchCD4bs | SL.glmnet | 0.738 (0.653, 0.824) | | 0.862 (0.805, 0.920) |
|  | geog.AAchESA | SL.randomForest | 0.735 (0.657, 0.813) | | 0.839 (0.773, 0.906) |
|  | Screen | Algorithm | AUC (cross validation) (CI) | | AUC (validated on dataset 1) (CI) |
| Dataset 2 | geog.AAchCD4bs | SL.randomForest | 0.866 (0.807, 0.926) | | 0.850 (0.782, 0.918) |
|  | all | SuperLearner | 0.854 (0.793, 0.914) | | 0.851 (0.779, 0.922) |
|  | all | SL.glmnet | 0.846 (0.779, 0.913) | | 0.865 (0.795, 0.935) |
|  | geog.AAchVRC01 | SL.randomForest | 0.846 (0.786, 0.905) | | 0.829 (0.757, 0.901) |
|  | all | SL.randomForest | 0.845 (0.785, 0.905) | | 0.887 (0.829, 0.944) |
|  | geog.glmnet | SL.randomForest | 0.840 (0.748, 0.932) | | 0.854 (0.751, 0.957) |
|  | geog.AAchCD4bs | SL.glmnet | 0.832 (0.740, 0.924) | | 0.822 (0.751, 0.893) |
|  | geog.corP | SL.randomForest | 0.824 (0.725, 0.924) | | 0.789 (0.675, 0.904) |
|  | geog.glmnet | SL.glmnet | 0.819 (0.751, 0.886) | | 0.848 (0.779, 0.918) |
|  | geog.glmnet | SL.glm | 0.818 (0.735, 0.901) | | 0.822 (0.754, 0.891) |
|  | geog.corP | SL.glmnet | 0.814 (0.731, 0.897) | | 0.810 (0.729, 0.891) |
|  | geog.glmnet | SL.naiveBayes | 0.800 (0.726, 0.874) | | 0.850 (0.784, 0.917) |
|  | geog.glmnet | SL.step | 0.803 (0.722, 0.884) | | 0.824 (0.753, 0.896) |
| Algorithms with coefficients >0.02 used in the SuperLearner | | | | | |
|  | Screen and algorithm | | | SuperLearner algorithm.coefficient | |
| Dataset 1 | geog.glmnet_SL.glmnet | | | 0.268 | |
|  | geog.corP_SL.naivebayes | | | 0.173 | |
|  | geog.glmnet_SL.step.interaction | | | 0.167 | |
|  | geog.AAchCD4bs_SL.naivebayes | | | 0.123 | |
|  | geog.AAchVRC01_SL.naivebayes | | | 0.091 | |
|  | geog.st_SL.step.interaction | | | 0.058 | |
|  | geog.AAchESA_SL.naivebayes | | | 0.024 | |
|  | geog.glmnet_SL.randomForest | | | 0.024 | |
| Dataset 2 | geog.AAchCD4bs_SL.randomForest | | | 0.443 | |
|  | geog.AAchgp41_SL.randomForest | | | 0.122 | |
|  | geog.glmnet_SL.step.interaction | | | 0.108 | |
|  | geog.corP_SL.step.interaction | | | 0.078 | |
|  | geog.AAchVRC01_SL.stumpboost | | | 0.070 | |
|  | geog.glmnet_SL.glm | | | 0.068 | |
|  | geog.glmnet_SL.naivebayes | | | 0.044 | |
|  | geog.AAchCD4bs_SL.naivebayes | | | 0.037 | |
|  | geog.corP_SL.randomForest | | | 0.025 | |
