# Supplementary material for: Prediction of VRC01 neutralization sensitivity by HIV-1 gp160 sequence features
Source: PLoS Comput Biol. 2019 Apr 1;15(4):e1006952. doi: 10.1371/journal.pcbi.1006952 (PMC6459550; doi:10.1371/journal.pcbi.1006952)
Supplement: S2 Table — Point estimates of the area under the receiver operating characteristic curve (AUC) are included for cross-validated performance within each of the two datasets, and for validation on the other separate data set. 95% confidence intervals are provided in parentheses. The Super Learner algorithm coefficients are the weights assigned by the ensemble to individual learners. (DOCX) [file pcbi.1006952.s014.docx]

S2 Table. The top ten performing models/algorithms and Super Learner, trained to predict the dichotomous sensitive/resistant only outcome, for Dataset 1 and Dataset 2. Point estimates of the area under the receiver operating characteristic curve (AUC) are included for cross-validated performance within each of the two datasets, and for validation on the other separate data set. 95% confidence intervals are provided in parentheses. The Super Learner algorithm coefficients are the weights assigned by the ensemble to individual learners.

|  | Screen | Algorithm | AUC (cross validation) (CI) | | AUC (validated on dataset 2) (CI) |
| --- | --- | --- | --- | --- | --- |
| Dataset 1 | all | SL.randomForest | 0.886 (0.834, 0.938) | | 0.925 (0.883, 0.968) |
|  | all | SuperLearner | 0.828 (0.764, 0.893) | | 0.921 (0.882, 0.959) |
|  | geog.AAchPNGS | SL.randomForest | 0.819 (0.758, 0.881) | | 0.784 (0.717, 0.852) |
|  | all | SL.glmnet | 0.816 (0.741, 0.890) | | 0.878 (0.824, 0.933) |
|  | geog.AAchGlyGP160 | SL.randomForest | 0.814 (0.739, 0.890) | | 0.673 (0.583, 0.764) |
|  | geog.AAchCD4bs | SL.randomForest | 0.812 (0.749, 0.875) | | 0.902 (0.856, 0.948) |
|  | geog.AAchVRC01 | SL.randomForest | 0.810 (0.737, 0.884) | | 0.896 (0.843, 0.950) |
|  | geog.AAchESA | SL.glmnet | 0.801 (0.733, 0.868) | | 0.838 (0.769, 0.908) |
|  | geog.glmnet | SL.glmnet | 0.795 (0.716, 0.873) | | 0.871 (0.818, 0.924) |
|  | geog.AAchESA | SL.randomForest | 0.771 (0.697, 0.846) | | 0.857 (0.791, 0.924) |
|  | geog.AAchCOVAR | SL.randomForest | 0.754 (0.672, 0.837) | | 0.766 (0.681, 0.850) |
|  | geog.glmnet | SL.randomForest | 0.740 (0.664, 0.816) | | 0.878 (0.821, 0.935) |
|  | geog.AAchVRC01 | SL.glmnet | 0.711 (0.595, 0.827) | | 0.856 (0.787, 0.926) |
|  | geog.AAchCD4bs | SL.glmnet | 0.745 (0.661, 0.829) | | 0.848 (0.787, 0.908) |
|  | geog.corP | SL.randomForest | 0.720 (0.628, 0.812) | | 0.844 (0.759, 0.930) |
|  | Screen | Algorithm | AUC (cross validation) (CI) | | AUC (validated on dataset 1) (CI) |
| Dataset 2 | all | SL.randomForest | 0.881 (0.822, 0.940) | | 0.904 (0.851, 0.957) |
|  | geog.AAchCD4bs | SL.randomForest | 0.873 (0.815, 0.931) | | 0.876 (0.812, 0.940) |
|  | all | SuperLearner | 0.872 (0.818, 0.925) | | 0.881 (0.813, 0.948) |
|  | geog.AAchVRC01 | SL.randomForest | 0.839 (0.774, 0.904) | | 0.858 (0.794, 0.923) |
|  | geog.corP | SL.naiveBayes | 0.813 (0.740, 0.886) | | 0.754 (0.665, 0.843) |
|  | geog.glmnet | SL.glmnet | 0.811 (0.739, 0.883) | | 0.880 (0.820, 0.940) |
|  | geog.AAchCD4bs | SL.glmnet | 0.802 (0.732, 0.871) | | 0.850 (0.787, 0.913) |
|  | geog.corP | SL.randomForest | 0.794 (0.689, 0.899) | | 0.772 (0.666, 0.877) |
|  | geog.glmnet | SL.randomForest | 0.783 (0.679, 0.886) | | 0.862 (0.778, 0.947) |
|  | geog.AAchESA | SL.randomForest | 0.780 (0.705, 0.856) | | 0.833 (0.766, 0.901) |
|  | all | SL.glmnet | 0.779 (0.697, 0.862) | | 0.883 (0.822, 0.944) |
|  | geog.glmnet | SL.step | 0.663 (0.544, 0.781) | | 0.840 (0.746, 0.933) |
|  | geog.AAchVRC01 | SL.glmnet | 0.747 (0.668, 0.825) | | 0.833 (0.768, 0.898) |
| Algorithms with coefficients >0.02 used in the SuperLearner | | | | | |
|  | Screen and algorithm | | | SuperLearner algorithm.coefficient | |
| Dataset 1 | geog.AAchCD4bs_SL.randomForest | | | 0.298 | |
|  | geog.AAchCD4bs_SL.naivebayes | | | 0.162 | |
|  | geog.glmnet_SL.glmnet | | | 0.117 | |
|  | all_SL.randomForest | | | 0.107 | |
|  | geog.glmnet_SL.randomForest | | | 0.085 | |
|  | geog.AAchVRC01_SL.randomForest | | | 0.071 | |
|  | geog.glmnet_SL.glm | | | 0.056 | |
|  | geog.AAchESA_SL.naivebayes | | | 0.051 | |
|  | geog.AAchGlyGP160_SL.naivebayes | | | 0.031 | |
| Dataset 2 | geog.AAchCD4bs_SL.randomForest | | | 0.457 | |
|  | geog.glmnet_SL.glmnet | | | 0.209 | |
|  | geog.corP_SL.glm | | | 0.077 | |
|  | geog.corP_SL.step | | | 0.077 | |
|  | geog.AAchgp41_SL.randomForest | | | 0.069 | |
|  | geog.corP_SL.naivebayes | | | 0.050 | |
|  | geog.glmnet_SL.step.interaction | | | 0.032 | |
