# Supplementary material for: Prediction of VRC01 neutralization sensitivity by HIV-1 gp160 sequence features
Source: PLoS Comput Biol. 2019 Apr 1;15(4):e1006952. doi: 10.1371/journal.pcbi.1006952 (PMC6459550; doi:10.1371/journal.pcbi.1006952)
Supplement: S3 Table — Point estimates of the area under the receiver operating characteristic curve (AUC) are included for cross-validated performance within each of the two datasets, and for validation on the other separate data set. 95% confidence intervals are provided in parentheses. The Super Learner algorithm coefficients are the weights assigned by the ensemble to individual learners. (DOCX) [file pcbi.1006952.s015.docx]

S3 Table. The top ten performing models/algorithms and Super Learner, trained to predict the quantitative log IC_50_ outcome, for dataset 1 and dataset 2. Point estimates of the R^2^ are included for cross-validated performance within each of the two datasets, and for validation on the other separate data set. 95% confidence intervals are provided in parentheses. The Super Learner algorithm coefficients are the weights assigned by the ensemble to individual learners.

|  | Screen | Algorithm | R^2^ (cross validation) (CI) | | R^2^ (validated on dataset 2) (CI) |
| --- | --- | --- | --- | --- | --- |
| Dataset 1 | all | SuperLearner | 0.349 (0.259, 0.429) | | 0.331 (0.277, 0.380) |
|  | all | SL.randomForest | 0.337 (0.286, 0.383) | | 0.366 (0.320, 0.409) |
|  | geog.glmnet | SL.randomForest | 0.327 (0.264, 0.385) | | 0.329 (0.270, 0.382) |
|  | all | SL.glmnet | 0.326 (0.263, 0.384) | | 0.211 (0.122, 0.291) |
|  | geog.AAchVRC01 | SL.randomForest | 0.292 (0.222, 0.356) | | 0.348 (0.292, 0.399) |
|  | geog.AAchCD4bs | SL.randomForest | 0.272 (0.197, 0.340) | | 0.320 (0.261, 0.375) |
|  | all | SL.xgboost | 0.261 (0.176, 0.338) | | 0.217 (0.131, 0.294) |
|  | geog.glmnet | SL.xgboost | 0.244 (0.159, 0.321) | | 0.112 (0.002, 0.209) |
|  | geog.AAchGlyGP160 | SL.randomForest | 0.233 (0.165, 0.295) | | 0.094 (0.004, 0.175) |
|  | geog.AAchCD4bs | SL.xgboost | 0.229 (0.144, 0.305) | | 0.253 (0.177, 0.322) |
|  | geog.AAchESA | SL.randomForest | 0.228 (0.158, 0.292) | | 0.287 (0.226, 0.344) |
|  | geog.AAchVRC01 | SL.glmnet | 0.173 (0.105, 0.236) | | 0.241 (0.182, 0.296) |
|  | geog.corP | SL.randomForest | 0.182 (0.106, 0.251) | | 0.241 (0.173, 0.303) |
|  | geog.AAchCD4bs | SL.glmnet | 0.198 (0.128, 0.263) | | 0.232 (0.166, 0.293) |
|  | geog.AAchESA | SL.xgboost | 0.120 (0.022, 0.209) | | 0.222 (0.150, 0.288) |
|  | Screen | Algorithm | R^2^ (cross validation) (CI) | | R^2^ (validated on dataset 1) (CI) |
| Dataset 2 | all | SL.randomForest | 0.303 (0.251, 0.352) | | 0.396 (0.349, 0.439) |
|  | geog.AAchCD4bs | SL.randomForest | 0.299 (0.241, 0.354) | | 0.354 (0.290, 0.412) |
|  | all | SuperLearner | 0.261 (0.183, 0.332) | | 0.379 (0.327, 0.427) |
|  | geog.AAchVRC01 | SL.randomForest | 0.239 (0.175, 0.298) | | 0.357 (0.299, 0.410) |
|  | geog.glmnet | SL.randomForest | 0.214 (0.136, 0.286) | | 0.335 (0.261, 0.402) |
|  | geog.AAchCD4bs | SL.glmnet | 0.211 (0.157, 0.261) | | 0.233 (0.170, 0.291) |
|  | geog.AAchCD4bs | SL.xgboost | 0.206 (0.131, 0.275) | | 0.276 (0.204, 0.342) |
|  | geog.corP | SL.randomForest | 0.193 (0.124, 0.258) | | 0.216 (0.133, 0.292) |
|  | geog.AAchESA | SL.randomForest | 0.183 (0.107, 0.252) | | 0.292 (0.230, 0.350) |
|  | geog.corP | SL.glmnet | 0.173 (0.110, 0.231) | | 0.207 (0.135, 0.273) |
|  | geog.AAchVRC01 | SL.glmnet | 0.171 (0.113, 0.225) | | 0.240 (0.177, 0.298) |
|  | all | SL.glmnet | 0.169 (0.109, 0.225) | | 0.257 (0.199, 0.311) |
|  | all | SL.xgboost | 0.168 (0.075, 0.253) | | 0.245 (0.150, 0.329) |
|  | geog.glmnet | SL.xgboost | 0.096 (-0.014, 0.195) | | 0.241 (0.148, 0.324) |
| Algorithms with coefficients >0.02 used in the SuperLearner | | | | | |
|  | Screen and algorithm | | | SuperLearner algorithm.coefficient | |
| Dataset 1 | geog.AAchVRC01_SL.randomForest | | | 0.179 | |
|  | all_SL.randomForest | | | 0.164 | |
|  | geog.glmnet_SL.step | | | 0.158 | |
|  | geog.AAchCD4bs_SL.stumpboost | | | 0.150 | |
|  | all_SL.stumpboost | | | 0.104 | |
|  | geog.glmnet_SL.glm | | | 0.095 | |
|  | geog.AAchGlyGP160_SL.randomForest | | | 0.067 | |
|  | geog.AAchGlyGP160_SL.stumpboost | | | 0.034 | |
|  | geog.AAchCD4bs_SL.randomForest | | | 0.023 | |
|  | geog.glmnet_SL.stumpboost | | | 0.022 | |
| Dataset 2 | geog.AAchCD4bs_SL.randomForest | | | 0.400 | |
|  | all_SL.randomForest | | | 0.307 | |
|  | all_SL.stumpboost | | | 0.136 | |
|  | geog.corP_SL.step.interaction | | | 0.088 | |
|  | geog.sequonCt_SL.step.interaction | | | 0.054 | |
