# Supplementary material for: Prediction of VRC01 neutralization sensitivity by HIV-1 gp160 sequence features
Source: PLoS Comput Biol. 2019 Apr 1;15(4):e1006952. doi: 10.1371/journal.pcbi.1006952 (PMC6459550; doi:10.1371/journal.pcbi.1006952)
Supplement: S4 Table — Point estimates of the area under the receiver operating characteristic curve (AUC) are included for cross-validated performance within each of the two datasets, and for validation on the other separate data set. 95% confidence intervals are provided in parentheses. The Super Learner algorithm coefficients are the weights assigned by the ensemble to individual learners. (DOCX) [file pcbi.1006952.s016.docx]

S4 Table. The top ten performing models/algorithms and Super Learner, trained to predict the quantitative log IC_80_ outcome, for dataset 1 and dataset 2. Point estimates of the R^2^ are included for cross-validated performance within each of the two datasets, and for validation on the other separate data set. 95% confidence intervals are provided in parentheses. The Super Learner algorithm coefficients are the weights assigned by the ensemble to individual learners.

|  | Screen | Algorithm | R^2^ (cross validation) (CI) | | R^2^ (validated on dataset 2) (CI) |
| --- | --- | --- | --- | --- | --- |
| Dataset 1 | geog.AAchCD4bs | SL.glmnet | 0.207 (0.139, 0.270) | | 0.160 (0.088, 0.225) |
|  | all | SL.randomForest | 0.203 (0.133, 0.268) | | 0.205 (0.139, 0.267) |
|  | all | SL.glmnet | 0.194 (0.108, 0.273) | | 0.194 (0.122, 0.260) |
|  | geog.AAchCD4bs | SL.randomForest | 0.185 (0.095, 0.266) | | 0.166 (0.064, 0.257) |
|  | geog.glmnet | SL.randomForest | 0.162 (0.051, 0.261) | | 0.212 (0.122, 0.293) |
|  | geog.AAchGlyGP160 | SL.randomForest | 0.156 (0.062, 0.241) | | -0.038 (-0.161, 0.072) |
|  | geog.AAchCD4bs | SL.xgboost | 0.148 (0.035, 0.248) | | 0.110 (-0.021, 0.224) |
|  | geog.glmnet | SL.xgboost | 0.126 (-0.023, 0.253) | | 0.079 (-0.070, 0.206) |
|  | geog.corP | SL.randomForest | 0.118 (0.021, 0.205) | | 0.191 (0.114, 0.261) |
|  | geog.corP | SL.step.interaction | 0.115 (-0.005, 0.222) | | 0.167 (0.064, 0.259) |
|  | all | SuperLearner | 0.102 (-0.046, 0.229) | | 0.208 (0.131, 0.278) |
|  | geog.AAchVRC01 | SL.randomForest | 0.099 (-0.010, 0.196) | | 0.216 (0.137, 0.288) |
|  | geog.corP | SL.glmnet | 0.090 (-0.019, 0.188) | | 0.177 (0.088, 0.258) |
|  | geog.corP | SL.xgboost | 0.003 (-0.105, 0.100) | | 0.177 (0.087, 0.258) |
|  | geog.corP | SL.glm | 0.100 (-0.012, 0.200) | | 0.177 (0.087, 0.258) |
|  | geog.corP | SL.step | 0.100 (-0.012, 0.200) | | 0.177 (0.087, 0.258) |
|  | Screen | Algorithm | R^2^ (cross validation) (CI) | | R^2^ (validated on dataset 1) (CI) |
| Dataset 2 | geog.AAchCD4bs | SL.randomForest | 0.252 (0.180, 0.317) | | 0.208 (0.103, 0.301) |
|  | geog.AAchVRC01 | SL.randomForest | 0.231 (0.159, 0.298) | | 0.220 (0.115, 0.313) |
|  | all | SuperLearner | 0.208 (0.120, 0.287) | | 0.238 (0.148, 0.318) |
|  | geog.corP | SL.randomForest | 0.204 (0.134, 0.268) | | 0.178 (0.079, 0.266) |
|  | geog.AAchCD4bs | SL.glmnet | 0.191 (0.116, 0.261) | | 0.137 (0.037, 0.226) |
|  | geog.corP | SL.step.interaction | 0.191 (0.108, 0.265) | | 0.138 (0.006, 0.253) |
|  | all | SL.randomForest | 0.189 (0.124, 0.249) | | 0.275 (0.208, 0.336) |
|  | geog.AAchESA | SL.randomForest | 0.159 (0.074, 0.236) | | 0.142 (0.038, 0.235) |
|  | geog.corP | SL.glm | 0.151 (0.084, 0.213) | | 0.174 (0.077, 0.262) |
|  | geog.corP | SL.step | 0.151 (0.084, 0.213) | | 0.174 (0.077, 0.262) |
|  | geog.corP | SL.glmnet | 0.148 (0.086, 0.205) | | 0.172 (0.080, 0.255) |
|  | geog.corP | SL.xgboost | 0.070 (0.005, 0.130) | | 0.174 (0.077, 0.262) |
|  | geog.AAchCD4bs | SL.xgboost | 0.114 (0.015, 0.202) | | 0.169 (0.063, 0.263) |
|  | geog.glmnet | SL.randomForest | 0.114 (-0.005, 0.218) | | 0.168 (0.046, 0.275) |
| Algorithms with coefficients >0.02 used in the SuperLearner | | | | | |
|  | Screen and algorithm | | | SuperLearner algorithm.coefficient | |
| Dataset 1 | geog.AAchCD4bs_SL.stumpboost | | | 0.250 | |
|  | geog.AAchGlyGP160_SL.randomForest | | | 0.211 | |
|  | geog.AAchCD4bs_SL.glmnet | | | 0.179 | |
|  | geog.glmnet_SL.step | | | 0.167 | |
|  | all_SL.stumpboost | | | 0.121 | |
|  | geog.AAchGlyGP160_SL.stumpboost | | | 0.052 | |
| Dataset 2 | geog.AAchCD4bs_SL.randomForest | | | 0.589 | |
|  | geog.corP_SL.step.interaction | | | 0.224 | |
|  | geog.AAchgp41_SL.randomForest | | | 0.072 | |
|  | geog.sequonCt_SL.step.interaction | | | 0.064 | |
|  | geog.AAchCD4bs_SL.stumpboost | | | 0.039 | |
