# Supplementary material for: Prediction of VRC01 neutralization sensitivity by HIV-1 gp160 sequence features
Source: PLoS Comput Biol. 2019 Apr 1;15(4):e1006952. doi: 10.1371/journal.pcbi.1006952 (PMC6459550; doi:10.1371/journal.pcbi.1006952)
Supplement: S5 Table — Point estimates of the area under the receiver operating characteristic curve (AUC) are included for cross-validated performance within each of the two datasets, and for validation on the other separate data set. 95% confidence intervals are provided in parentheses. The Super Learner algorithm coefficients are the weights assigned by the ensemble to individual learners. (DOCX) [file pcbi.1006952.s017.docx]

S5 Table. The top ten performing models/algorithms and Super Learner, trained to predict the neutralization slope outcome, for dataset 1 and dataset 2. Point estimates of the R^2^ are included for cross-validated performance within each of the two datasets, and for validation on the other separate data set. 95% confidence intervals are provided in parentheses. The Super Learner algorithm coefficients are the weights assigned by the ensemble to individual learners.

|  | Screen | Algorithm | R^2^ (cross validation) (CI) | | R^2^ (validated on dataset 2) (CI) |
| --- | --- | --- | --- | --- | --- |
| Dataset 1 | all | SL.randomForest | 0.100 (0.032, 0.163) | | 0.065 (-0.003, 0.128) |
|  | geog.sequonCt | SL.glmnet | 0.093 (0.017, 0.164) | | 0.087 (0.009, 0.159) |
|  | geog.AAchVRC01 | SL.glmnet | 0.093 (0.035, 0.149) | | 0.068 (0.014, 0.120) |
|  | geog.AAchGLYCO | SL.glmnet | 0.093 (0.044, 0.139) | | 0.048 (-0.005, 0.098) |
|  | geog.AAchESA | SL.glmnet | 0.077 (0.010, 0.140) | | 0.070 (0.019, 0.118) |
|  | geog.sbulk | SL.glmnet | 0.072 (0.007, 0.133) | | 0.053 (-0.011, 0.112) |
|  | geog.AAchgp41 | SL.glmnet | 0.069 (0.015, 0.120) | | NA |
|  | all | SuperLearner | 0.066 (-0.035, 0.158) | | 0.110 (0.042, 0.172) |
|  | geog.st | SL.glmnet | 0.062 (0.020, 0.102) | | 0.027 (-0.022, 0.073) |
|  | geog.sequonCt | SL.glm | 0.059 (-0.068, 0.171) | | 0.073 (-0.021, 0.159) |
|  | geog.sequonCt | SL.step | 0.059 (-0.068, 0.171) | | 0.073 (-0.021, 0.159) |
|  | all | SL.glmnet | 0.003 (-0.059, 0.062) | | 0.071 (-0.008, 0.143) |
|  | geog.sequonCt | SL.randomForest | 0.055 (-0.075, 0.170) | | 0.071 (-0.049, 0.177) |
|  | geog.glmnet | SL.glmnet | -0.226 (-0.569, 0.042) | | 0.067 (-0.066, 0.184) |
|  | geog.AAchVRC01 | SL.randomForest | 0.049 (-0.050, 0.139) | | 0.066 (-0.033, 0.154) |
|  | Screen | Algorithm | R^2^ (cross validation) (CI) | | R^2^ (validated on dataset 1) (CI) |
| Dataset 2 | geog.sequonCt | SL.glmnet | 0.084 (-0.002, 0.162) | | 0.077 (-0.017, 0.163) |
|  | geog.sequonCt | SL.glm | 0.043 (-0.076, 0.150) | | 0.043 (-0.085, 0.156) |
|  | geog.sequonCt | SL.step | 0.043 (-0.076, 0.150) | | 0.043 (-0.085, 0.156) |
|  | geog.sequonCt | SL.randomForest | 0.024 (-0.093, 0.129) | | 0.017 (-0.105, 0.126) |
|  | all | SL.randomForest | 0.024 (-0.049, 0.091) | | 0.008 (-0.090, 0.098) |
|  | geog.AAchGlyGP160 | SL.glmnet | 0.022 (-0.023, 0.065) | | 0.056 (-0.001, 0.110) |
|  | all | SL.glmnet | 0.021 (-0.028, 0.067) | | -0.032 (-0.127, 0.054) |
|  | geog.AAchGLYCO | SL.randomForest | 0.017 (-0.116, 0.134) | | -0.247 (-0.555, 0.001) |
|  | geog.AAchGLYCO | SL.glmnet | 0.016 (-0.085, 0.107) | | -0.298 (-0.660, -0.015) |
|  | all | SuperLearner | 0.011 (-0.081, 0.096) | | 0.063 (-0.028, 0.147) |
|  | geog.geom | SL.glm | 0.010 (-0.096, 0.106) | | -0.030 (-0.166, 0.091) |
|  | geog.sbulk | SL.glmnet | -0.056 (-0.125, 0.009) | | 0.078 (0.038, 0.115) |
|  | geog.AAchESA | SL.randomForest | -0.163 (-0.304, -0.038) | | 0.075 (-0.016, 0.157) |
|  | geog.cys | SL.glm | -0.020 (-0.094, 0.048) | | 0.071 (0.007, 0.131) |
|  | geog.cys | SL.step | -0.020 (-0.094, 0.048) | | 0.071 (0.007, 0.131) |
|  | geog.cys | SL.step.interaction | -0.049 (-0.133, 0.029) | | 0.071 (0.007, 0.131) |
|  | geog | SL.glmnet | -0.001 (-0.053, 0.049) | | 0.071 (0.021, 0.119) |
|  | geog | SL.xgboost | 0.004 (-0.058, 0.063) | | 0.071 (0.007, 0.130) |
|  | geog | SL.glm | 0.004 (-0.058, 0.063) | | 0.071 (0.007, 0.130) |
|  | geog | SL.step.interaction | 0.004 (-0.058, 0.063) | | 0.071 (0.007, 0.130) |
|  | geog | SL.step | 0.004 (-0.058, 0.063) | | 0.071 (0.007, 0.130) |
| Algorithms with coefficients >0.02 used in the SuperLearner | | | | | |
|  | Screen and algorithm | | | SuperLearner algorithm.coefficient | |
| Dataset 1 | geog.AAchGlyGP160_SL.stumpboost | | | 0.229 | |
|  | geog.sequonCt_SL.step.interaction | | | 0.171 | |
|  | geog.geom_SL.stumpboost | | | 0.151 | |
|  | geog.AAchgp41_SL.randomForest | | | 0.141 | |
|  | geog.glmnet_SL.glmnet | | | 0.127 | |
|  | geog.AAchVRC01_SL.glmnet | | | 0.066 | |
|  | geog.AAchCD4bs_SL.randomForest | | | 0.064 | |
|  | geog.sequonCt_SL.randomForest | | | 0.047 | |
| Dataset 2 | geog.sequonCt_SL.glmnet | | | 0.292 | |
|  | geog.AAchGLYCO_SL.randomForest | | | 0.292 | |
|  | geog.geom_SL.step | | | 0.123 | |
|  | geog.geom_SL.glm | | | 0.123 | |
|  | geog.sequonCt_SL.randomForest | | | 0.097 | |
|  | geog.sequonCt_SL.step.interaction | | | 0.050 | |
