# Supplementary material for: Prediction of VRC01 neutralization sensitivity by HIV-1 gp160 sequence features
Source: PLoS Comput Biol. 2019 Apr 1;15(4):e1006952. doi: 10.1371/journal.pcbi.1006952 (PMC6459550; doi:10.1371/journal.pcbi.1006952)
Supplement: S6 Table — Features with a positive coefficient are associated with neutralization resistance, while features with a negative coefficient are associated with neutralization sensitivity. This table of coefficients can be used to build a linear model for classifying neutralization resistance from the content of a given HIV-1 envelope sequence. (DOCX) [file pcbi.1006952.s018.docx]

S6 Table. The 80 variables contributing to prediction of neutralization resistance by the *lasso with pre-screening of features by lasso* method and their estimated coefficients. Features with a positive coefficient are associated with VRC01 resistance, while features with a negative coefficient are associated with VRC01 sensitivity. This table of coefficients can be used to build a linear model for predicting VRC01 resistance from the content of a given HIV-1 envelope sequence.

| Variable | Coefficient |
| --- | --- |
| hxb2.279.E.1mer | 5.89065774 |
| hxb2.181.M.1mer | 4.61843726 |
| hxb2.456.R.1mer | -4.00165024 |
| hxb2.406.P.1mer | 3.97919283 |
| hxb2.456.H.1mer | 3.93788707 |
| hxb2.457.D.1mer | -3.6488855 |
| hxb2.397.Q.1mer | 3.62171427 |
| hxb2.321.T.1mer | 3.38782622 |
| hxb2.280.D.1mer | 3.29111212 |
| hxb2.459.G.1mer | -2.99951763 |
| hxb2.281.E.1mer | 2.67100335 |
| hxb2.386.Y.1mer | 2.6599456 |
| hxb2.268.sequon_actual.1mer | 2.6118435 |
| hxb2.365.R.1mer | 2.5833504 |
| hxb2.415.X.1mer | -2.57950375 |
| hxb2.655.N.1mer | 2.39400311 |
| hxb2.138.Q.1mer | 2.34345588 |
| hxb2.186.R.1mer | -2.26041186 |
| hxb2.461.R.1mer | 2.08014907 |
| hxb2.156.I.1mer | 2.01311443 |
| hxb2.365.V.1mer | 1.95814394 |
| hxb2.461.P.1mer | 1.94393878 |
| hxb2.429.A.1mer | 1.9123487 |
| hxb2.461.Q.1mer | 1.89204902 |
| hxb2.232.sequon_actual.1mer | 1.86405949 |
| hxb2.29.sequon_actual.1mer | 1.85723484 |
| hxb2.410.P.1mer | 1.7945506 |
| hxb2.455.E.1mer | 1.68517023 |
| hxb2.280.N.1mer | -1.64695162 |
| hxb2.144.Y.1mer | 1.60068456 |
| hxb2.179.I.1mer | 1.59348088 |
| hxb2.144.G.1mer | 1.57220042 |
| hxb2.190.F.1mer | 1.47217693 |
| hxb2.677.H.1mer | 1.45859762 |
| cysteines.total.v5 | 1.40846891 |
| hxb2.143.A.1mer | 1.39367373 |
| hxb2.408.K.1mer | 1.37706517 |
| hxb2.404.sequon_actual.1mer | -1.35232419 |
| hxb2.139.E.1mer | 1.33882426 |
| hxb2.179.Q.1mer | 1.32685847 |
| hxb2.278.S.1mer | 1.30133364 |
| hxb2.394.K.1mer | -1.29348054 |
| hxb2.406.V.1mer | 1.28915014 |
| hxb2.544.V.1mer | 1.23912743 |
| hxb2.150.A.1mer | 1.21497936 |
| subtype.is.A1 | -1.19590601 |
| hxb2.442.I.1mer | 1.19539921 |
| hxb2.471.I.1mer | 1.16940579 |
| hxb2.321.gap.1mer | -1.15472997 |
| hxb2.462.K.1mer | 1.11785871 |
| hxb2.279.D.1mer | -1.07510905 |
| hxb2.616.sequon_actual.1mer | -1.04158013 |
| hxb2.144.T.1mer | -0.92729373 |
| hxb2.186.K.1mer | 0.87440008 |
| hxb2.397.R.1mer | 0.86510397 |
| hxb2.408.V.1mer | 0.84602267 |
| hxb2.130.E.1mer | 0.84542692 |
| hxb2.396.L.1mer | 0.82436578 |
| hxb2.197.N.1mer | -0.7810908 |
| hxb2.362.E.1mer | 0.77889504 |
| hxb2.463.R.1mer | 0.75263471 |
| sequons.total.v5 | -0.73219773 |
| hxb2.139.D.1mer | 0.71842444 |
| hxb2.397.G.1mer | -0.70373139 |
| hxb2.406.R.1mer | 0.68128365 |
| hxb2.187.N.1mer | 0.65994006 |
| geographic.region.of.origin.is.S.Africa | 0.63287621 |
| hxb2.471.E.1mer | 0.62130403 |
| hxb2.360.sequon_actual.1mer | 0.55841473 |
| hxb2.408.S.1mer | -0.41876186 |
| subtype.is.C | 0.40382479 |
| hxb2.683.R.1mer | 0.35911048 |
| hxb2.371.V.1mer | 0.34323124 |
| hxb2.132.G.1mer | 0.33765778 |
| hxb2.460.T.1mer | 0.29331425 |
| hxb2.97.E.1mer | 0.26907793 |
| hxb2.463.E.1mer | 0.25290763 |
| hxb2.477.N.1mer | 0.08910589 |
| hxb2.241.K.1mer | -0.06441929 |
| length.env | 0.03325985 |
