# Supplementary material for: Prediction of VRC01 neutralization sensitivity by HIV-1 gp160 sequence features
Source: PLoS Comput Biol. 2019 Apr 1;15(4):e1006952. doi: 10.1371/journal.pcbi.1006952 (PMC6459550; doi:10.1371/journal.pcbi.1006952)
Supplement: S7 Table — Variable importance measure (VIM) information for the features that have a Holm-Bonferroni p-value less than 0.05, ranked by their contribution to the prediction of the (A) sensitive/resistant only outcome, (B) quantitative log IC80 outcome, or (C) neutralization slope outcome. (DOCX) [file pcbi.1006952.s019.docx]

S7 Table: Variable importance measure (VIM) information for the features that have a Holm-Bonferroni p-value less than 0.05, ranked by their contribution to the prediction of the (A) sensitive/resistant only outcome, (B) quantitative log IC_80_ outcome, or (C) neutralization slope outcome.

| Feature | MCCV Composite VIM | Ensemble VIM | Ensemble VIM Rank | Direction of Effect^1^ | p-value^2^ | q-value^3^ | FWER  p-value^4^ |
| --- | --- | --- | --- | --- | --- | --- | --- |
| A. Sensitive/Resistant Only Outcome | | | | | | | |
| 456 is R | 90.863 | 0.081 | 1 | Sensitive | 1.13E-33 | 9.33E-31 | 9.33E-31 |
| 459 is G | 69.595 | 0.057 | 2 | Sensitive | 7.46E-29 | 3.09E-26 | 6.17E-26 |
| 458 is G | 33.841 | 0.045 | 12 | Sensitive | 1.98E-22 | 5.47E-20 | 1.64E-19 |
| 280 is N | 33.769 | 0.051 | 5 | Sensitive | 4.77E-22 | 9.88E-20 | 3.94E-19 |
| 655 is N | 30.074 | 0.032 | 552 | Resistant | 6.14E-08 | 1.34E-06 | 4.86E-05 |
| Length of gp120 | 29.694 | 0.041 | 46 | Resistant | 6.75E-06 | 9.81E-05 | 0.005 |
| 181 is M | 27.983 | 0.033 | 464 | Resistant | 1.16E-06 | 1.84E-05 | 0.001 |
| 124 is P | 22.097 | 0.039 | 109 | Sensitive | 3.21E-16 | 2.21E-14 | 2.63E-13 |
| Length of Env | 17.365 | 0.038 | 126 | Resistant | 4.43E-06 | 6.80E-05 | 0.003 |
| 471 is I | 15.999 | 0.044 | 15 | Resistant | 6.60E-11 | 2.10E-09 | 5.30E-08 |
| 466 is E | 15.331 | 0.033 | 521 | Sensitive | 2.36E-17 | 2.79E-15 | 1.94E-14 |
| 428 is Q | 14.905 | 0.039 | 91 | Sensitive | 2.09E-16 | 1.92E-14 | 1.71E-13 |
| Total cysteines in Env | 14.21 | 0.029 | 749 | Resistant | 7.33E-07 | 1.24E-05 | 0.001 |
| 471 is G | 12.903 | 0.04 | 74 | Sensitive | 2.12E-10 | 6.05E-09 | 1.69E-07 |
| 469 is R | 12.653 | 0.036 | 285 | Sensitive | 7.79E-07 | 1.29E-05 | 0.001 |
| 589 is D | 10.891 | 0.032 | 574 | Sensitive | 6.31E-12 | 2.18E-10 | 5.08E-09 |
| 374 is H | 9.824 | 0.034 | 442 | Sensitive | 2.46E-15 | 1.57E-13 | 2.01E-12 |
| 425 is N | 9.71 | 0.033 | 494 | Sensitive | 1.40E-10 | 4.21E-09 | 1.13E-07 |
| 569 is T | 9.657 | 0.039 | 113 | Sensitive | 1.83E-13 | 7.20E-12 | 1.48E-10 |
| 456 is W | 9.223 | 0.034 | 443 | Resistant | 4.98E-10 | 1.33E-08 | 3.97E-07 |
| 616 is PNGS | 9.047 | 0.027 | 780 | Sensitive | 1.43E-10 | 4.21E-09 | 1.14E-07 |
| 365 is S | 8.432 | 0.035 | 341 | Sensitive | 1.99E-08 | 4.58E-07 | 1.58E-05 |
| Total cysteines in gp120 | 7.25 | 0.039 | 98 | Resistant | 6.34E-07 | 1.09E-05 | 4.95E-04 |
| 156 is PNGS | 5.877 | NA | 805 | Sensitive | 4.08E-09 | 9.65E-08 | 3.24E-06 |
| 156 is N | 5.871 | 0.038 | 138 | Sensitive | 4.08E-09 | 9.65E-08 | 3.24E-06 |
| 397 is C | 5.731 | 0.032 | 591 | Resistant | 2.48E-19 | 4.11E-17 | 2.05E-16 |
| 455 is E | 5.633 | 0.041 | 42 | Resistant | 4.56E-05 | 4.90E-04 | 0.034 |
| 459 is D | 5.259 | 0.041 | 34 | Resistant | 2.07E-08 | 4.63E-07 | 1.64E-05 |
| 619 is PNGS | 0.182 | 0.047 | 7 | Resistant | 1.51E-05 | 1.90E-04 | 0.012 |
| B. Quantitative Log IC_80_ Outcome | | | | | | | |
| 456 is R | 99.983 | 0.022 | 2 | Sensitive | 2.46E-12 | 1.94E-09 | 1.94E-09 |
| 459 is G | 76.911 | 0.036 | 1 | Sensitive | 2.03E-10 | 8.03E-08 | 1.60E-07 |
| 234 is PNGS | 43.672 | -0.001 | 5 | Resistant | 4.67E-06 | 7.39E-4 | 0.004 |
| Subtype is A1 | 32.107 | -0.015 | 663 | Sensitive | 2.20E-05 | 0.002 | 0.017 |
| 471 is G | 28.264 | 0.016 | 3 | Sensitive | 2.57E-06 | 6.78E-4 | 0.002 |
| C. Neutralization Slope Outcome | | | | | | | |
| 276 is PNGS | 47.211 | NA | 810 | Resistant | 1.23E-06 | 4.70E-4 | 9.39E-4 |
| Total PNG sites in Loop D | 26.124 | -0.021 | 1 | Resistant | 1.23E-06 | 4.70E-4 | 9.39E-4 |
| 459 is gap | 19.922 | -0.04 | 600 | Sensitive | 8.08E-06 | 0.002 | 0.006 |
| 276 is N | 4.005 | -0.04 | 615 | Resistant | 4.59E-05 | 0.009 | 0.035 |

Features shown were ranked among the top 50 features by either VIM method and had a Holm-Bonferroni 2-sided p-value less than 0.05 for an association with the outcome in a (A) logistic or (B, C) linear regression model using both datasets (with adjustment for geographic region as in all analyses).

^1^ When the direction of effect is “Sensitive” (“Resistant”), the presence of or a higher quantity of the feature associates with VRC01 sensitivity (resistance).

^2^The p-value is from a Wald test in a (A) logistic or (B, C) linear regression model testing the association of the feature with outcome, controlling for the sequences’ geographic region of origin information to control for possible confounding.

^3^The q-value is the Benjamini-Hochberg false discovery rate.

^4^ The FWER p-value is the Holm-Bonferroni family-wise error-rate adjusted p-value.

FWER, family-wise error rate; MCCV, Monte Carlo cross-validation; VIM, variable importance measure.
