# Supplementary material for: Prediction of VRC01 neutralization sensitivity by HIV-1 gp160 sequence features
Source: PLoS Comput Biol. 2019 Apr 1;15(4):e1006952. doi: 10.1371/journal.pcbi.1006952 (PMC6459550; doi:10.1371/journal.pcbi.1006952)
Supplement: S8 Table — (DOCX) [file pcbi.1006952.s020.docx]

S8 Table: Numbers of HIV-1 Envelope sequences in the CATNAP database by subtype/recombinant subtype, country of origin, and geographic region of origin, broken down by dataset 1, dataset 2, and all sequences (datasets 1+2 combined).

| Subtype | All | Dataset 1 | Dataset 2 |
| --- | --- | --- | --- |
| 01_AE | 32 | 16 | 16 |
| 02_AG | 12 | 6 | 6 |
| 07_BC | 39 | 22 | 17 |
| 08_BC | 6 | 4 | 2 |
| 14_BG | 1 | 0 | 1 |
| A1 | 50 | 28 | 22 |
| B | 81 | 38 | 43 |
| C | 293 | 140 | 153 |
| D | 24 | 13 | 11 |
| G | 9 | 5 | 4 |
| N | 2 | 0 | 2 |
| O | 12 | 6 | 6 |
| P | 1 | 1 | 0 |
|  |  |  |  |
| Recombinant Subtype | All | Dataset 1 | Dataset 2 |
| 02A1 | 4 | 3 | 1 |
| A1C | 10 | 6 | 4 |
| A1CD | 2 | 1 | 1 |
| A1D | 14 | 5 | 9 |
| A1G | 1 | 0 | 1 |
| A2D | 3 | 2 | 1 |
| BC | 4 | 3 | 1 |
| BG | 1 | 1 | 0 |
| CD | 8 | 4 | 4 |
| DO | 1 | 1 | 0 |
| DU | 1 | 1 | 0 |
|  |  |  |  |
| Country of Origin | All | Dataset 1 | Dataset 2 |
| Belgium | 5 | 3 | 2 |
| Botswana | 6 | 3 | 3 |
| Brazil | 3 | 1 | 2 |
| Cameroon | 22 | 11 | 11 |
| China | 97 | 48 | 49 |
| Cote d'Ivoire | 1 | 1 | 0 |
| Dem. Repub. Congo | 1 | 1 | 0 |
| Ethiopia | 2 | 1 | 1 |
| France | 13 | 6 | 7 |
| Haiti | 1 | 0 | 1 |
| India | 12 | 6 | 6 |
| Italy | 2 | 1 | 1 |
| Kenya | 50 | 25 | 25 |
| Malawi | 61 | 30 | 31 |
| Peru | 12 | 6 | 6 |
| Rwanda | 6 | 3 | 3 |
| South Africa | 137 | 69 | 68 |
| Spain | 12 | 6 | 6 |
| Tanzania | 63 | 32 | 31 |
| Thailand | 17 | 8 | 9 |
| Trinidad and Tobago | 5 | 2 | 3 |
| Uganda | 25 | 13 | 12 |
| United States | 43 | 22 | 21 |
| Zambia | 15 | 8 | 7 |
|  |  |  |  |
| Geographic Region of Origin | All | Set 1 | Set 2 |
| Asia | 126 | 62 | 64 |
| Europe/Americas | 96 | 47 | 49 |
| N. Africa | 170 | 87 | 83 |
| S. Africa | 219 | 110 | 109 |
