# Supplementary material for: Prediction of VRC01 neutralization sensitivity by HIV-1 gp160 sequence features
Source: PLoS Comput Biol. 2019 Apr 1;15(4):e1006952. doi: 10.1371/journal.pcbi.1006952 (PMC6459550; doi:10.1371/journal.pcbi.1006952)
Supplement: S1 Text — (DOCX) [file pcbi.1006952.s021.docx]

Supplementary Text for “Prediction of VRC01 neutralization sensitivity by HIV-1 gp160 sequence features” by Magaret, Benkeser, and Williamson et al.

This text was adapted from the Statistical Analysis Plan for developing a VRC01 TZM-bl in vitro neutralization resistance score based on HIV-1 gp160 sequence features. It describes the feature selection process and provides additional details related to the analysis.

**Table of Contents**

CATNAP data set restructuring 2

Annotation data 2

Super learning considerations 2

Feature Groups: Additional information for the amino acid site sets constituting the sites of interest for the analysis 2

Feature **Group** **1**: The VRC01 binding footprint 2

Feature **Group** **2**: CD4 binding sites 2

Feature **Group** **3**: Sites with sufficient exposed surface area 3

Feature **Group** **4**: Sites identified as important for glycosylation 3

Feature **Group 5**: Sites with residues that covary with the VRC01 binding footprint 3

Feature **Group 6**: Sites associated with VRC01-specific potential N-linked glycosylation (PNGS) effects 3

Feature **Group 7**: Sites in gp41 associated with VRC01 neutralization or sensitivity in one or more publications, corresponding to the sites identified in [7-16]. 3

Combining Feature **Groups** **1** through **7** and application of the minimum variability criterion 4

Representation of sites in Feature **Groups** **1**-**6** in the analysis data sets 4

Feature Groups: Additional information for the other genotypic features included as input features 4

Feature **Group** **8**: Sites for indicating N-linked glycosylation 4

Feature **Group** **9**: Majority subtypes 4

Feature **Group** **10**: Region-specific counts of PNGS 5

Feature **Group** **11**: Viral geometry 5

Feature **Group** **12**: Cysteine counts 5

Feature **Group** **13**: Steric bulk at critical locations 5

Creation of new TZM-bl neutralization resistance outcome variables from the CATNAP data (infectivity results) 5

Single vs. multiple studies of VRC01-mediated neutralization of a given HIV-Env pseudovirus 6

Handling right-censored values 6

Supplementary References 7

## CATNAP data set restructuring

Sequence-related annotation and infectivity results were retrieved from CATNAP [1] and restructured as described below for the analysis:

### Annotation data

Countries of origin were reclassified into geographic regions of origin [“Asia” (107 total, 18.2%) = China (78), India (12), and Thailand (17); “N.Africa” (147 total, 29.0%) = Cameroon (22), Cote d'Ivoire (1), Democratic Republic of the Congo (1), Ethiopia (2), Kenya (58), Rwanda (6), Tanzania (62), and Uganda (19); “S.Africa” (218 total, 37.1%) = Botswana (6), Malawi (61), South Africa (136), and Zambia (15); and “Europe.Americas” (92 total, 15.7%) = Belgium (5), Brazil (3), France (12), Haiti (1), Italy (2), Peru (12), Spain (12), Trinidad and Tobago (4), and the United States (41)]. Geographic region information was rendered as binary indicator variables. Coreceptor (CCR5, CXCR4) information was rendered as binary indicator variables. Neutralization sensitivity tier was converted into an ordinal monotonic form (1 = Tier 1A, 2 = Tier 1, 3 = Tier 1B, 4 = Tier “1 or 2”, 5 = Tier “1B or 2”, 6 = Tier 2, 7 = Tier “2 or 3”, 8 = Tier 3).

## Super learning considerations

In this section, we explore benefits and drawbacks of the Super Learner approach in our analysis. An appealing argument for utilizing Super Learner is that it permits one to explore many different learning algorithms in a fully pre-specified manner. This is crucial for drawing valid statistical inference about our prediction model’s performance, which was a primary objective of our work.

A potential drawback of the Super Learner is that one may risk overfitting when too many learners are included as candidates. The estimation of the Super Learner weights is comparable to a regression with the number of predictors in the regression model equal to the number of candidate learners considered. Our analysis took a fairly aggressive approach by including >50 candidate learners for each outcome. Nevertheless, the cross-validated performance measures indicated consistently strong performance of the ensemble model. It would, however, be interesting to see whether ensemble performance could be improved (or at least similar performance achieved) by including fewer learners. This is an important consideration for future research in this area, as it could cut down considerably on computational cost.

## Feature Groups: Additional information for the amino acid site sets constituting the sites of interest for the analysis

### Feature **Group** **1**: The VRC01 binding footprint

Amino acid sites: **97, 123, 124, 198, 276, 278, 279, 280, 281, 282, 365, 366, 367, 368, 371, 427, 428, 429, 430, 455, 456, 457, 458, 459, 460, 461, 463, 465, 466, 467, 469, 472, 473, 474 and 476.**

### Feature **Group** **2**: CD4 binding sites

Amino acid sites: **124, 125, 126, 127, 196, 198, 279, 280, 281, 282, 283, 365, 366, 367, 368, 369, 370, 374, 425, 426, 427, 428, 429, 430, 431, 432, 455, 456, 457, 458, 459, 460, 461, 469, 471, 472, 473, 474, 475, 476 and 477.**

### Feature **Group** **3**: Sites with sufficient exposed surface area

The Dictionary of Protein Secondary Structure was used to calculate the buried surface area of each of the 35 amino acids identified in [2] as a contact site between VRC01 and HIV-1 Env using Protein Data Bank IDs 3NGB, 4LSS, 4LST, 5FYJ, and 5FYK [3, 4]. The Env protein sequences were aligned to reference strain HXB2 using HIVAlign [5]. Env residues were sorted by maximum buried surface area across the five structures and then divided into three groups based on manually selected cutoffs based on breaks observed in the average surface area. Amino acid sites in the first two groups (i.e. with the least buried and next-to-least buried surface areas) were included in the analysis as input genotypic features.

Amino acid sites: **97, 198, 276, 278, 279, 280, 281, 282, 365, 366, 367, 368, 371, 415, 428, 429, 430, 455, 457, 458, 459, 460, 461, 467, 469, 473, 474 and 476.**

### Feature **Group** **4**: Sites identified as important for glycosylation

We included all AA positions pertaining the glycan fence identified by Stewart-Jones et al. [7] and Crooks et al. [8,9] and the sites identified by Stewart-Jones et al. [7] where VRC01 interacts with the trimer. Aggregated together, these sites are:

Amino acid sites: **61, 64, 197, 276, 362, 363, 386, 392, 462 and 463.**

### Feature **Group 5**: Sites with residues that covary with the VRC01 binding footprint

Covariability analysis was conducted separately for subtypes A (including A1 and A2), B, and C. For each subtype set, and for every pair of sites where (a) both sites pass the minimum variability filter, and (b) one site is outside the VRC01 binding footprint and one is within the VRC01 binding footprint, the normalized mutual information statistic and a 2-sided p-value was calculated to test for covariability. Q-values were computed for all analyzed site pairs. Sites outside of the VRC01 binding footprint with q < 0.001 for at least two of the three subtype analyses were added to the list of positions for the primary analysis. The rationale is that documented covariability (and falling far short of collinearity) may reflect structural or functional constraints of connected amino acid positions, such that the connected positions could potentially be involved in VRC01 escape in a coordinated way.

Amino acid sites: **46, 132, 138, 144, 150, 179, 181, 186, 190, 290, 321, 328, 354, 389, 394, 396, 397 and 406.**

### Feature **Group 6**: Sites associated with VRC01-specific potential N-linked glycosylation (PNGS) effects

A Bayesian machine learning approach that assessed bNAb binding against a panel of 94 recombinant gp120 monomers [6] identified these sites as being associated with potential N-linked glycosylation, where the presence or absence of a PNGS results in improved or reduced VRC01 binding.

Amino acid sites: **130, 139, 143, 156, 187, 197, 241, 262, 289, 339, 355, 363, 406, 408, 410, 442, 448, 460 and 462.**

### Feature **Group 7**: Sites in gp41 associated with VRC01 neutralization or sensitivity in one or more publications, corresponding to the sites identified in [7-16].

Amino acid sites: **544, 569, 582, 589, 655, 668, 675, 677, 680, 681, 683, 688, and 702.**

### Combining Feature **Groups** **1** through **7** and application of the minimum variability criterion

Combining Feature **Groups 1** through **7** results in the following sites (n=104):

**46, 61, 64, 97, 123, 124, 125, 126, 127, 130, 132, 138, 139, 143, 144, 150, 156, 179, 181, 186, 187, 190, 196, 197, 198, 241, 262, 276, 278, 279, 280, 281, 282, 283, 289, 290, 321, 328, 339, 354, 355, 362, 363, 365, 366, 367, 368, 369, 370, 371, 374, 386, 389, 392, 394, 396, 397, 406, 408, 410, 415, 425, 426, 427, 428, 429, 430, 431, 432, 442, 448, 455, 456, 457, 458, 459, 460, 461, 462, 463, 465, 466, 467, 469, 471, 472, 473, 474, 475, 476, 477, 544, 569, 582, 589, 655, 668, 675, 677, 680, 681, 683, 688, and 702.**

Application of the minimum variability filter described in Methods, “Input Variable Set”, to the 104 sites listed above yields the following 91 sites to be included in the analysis:

**46, 61, 97, 124, 125, 127, 130, 132, 138, 139, 143, 144, 150, 156, 179, 181, 186, 187, 190, 197, 198, 241, 276, 278, 279, 280, 281, 282, 283, 289, 290, 321, 328, 339, 354, 355, 362, 363, 365, 369, 371, 374, 386, 389, 392, 394, 396, 397, 406, 408, 410, 415, 425, 426, 428, 429, 430, 431, 432, 442, 448, 455, 456, 457, 458, 459, 460, 461, 462, 463, 465, 466, 467, 469, 471, 474, 475, 476, 477, 544, 569, 589, 655, 668, 675, 677, 680, 681, 683, 688 and 702.**

### Representation of sites in Feature **Groups** **1**-**6** in the analysis data sets

Each of these sites was represented in the analysis data sets by binary indicators for the presence or absence of amino acid residue information at these positions. There was one indicator for each residue observed at each location.

## Feature Groups: Additional information for the other genotypic features included as input features

### Feature **Group** **8**: Sites for indicating N-linked glycosylation

Sites were included if at least ten sequences in the database featured the lead position of the canonical N-linked glycosylation motif ([N][S|T][!P])[6], and if at least ten sequences did not have this motif. These sites were represented by binary indicator variables. N-linked glycosylation indicators were not be included for sites that are insertions relative to HXB2, due to the fact that these sites are be difficult to precisely reproduce in a different alignment.

The first-positions of PNGSes for analysis are: **29, 49, 59, 130, 133, 134, 135, 136, 137, 138, 139, 140, 141, 142, 143, 144, 145, 146, 147, 148, 149, 150, 156, 160, 185, 186, 187, 188, 197, 229, 230, 234, 241, 276, 289, 293, 295, 301, 332, 334, 337, 339, 344, 354, 355, 356, 358, 360, 362, 363, 386, 392, 393, 394, 396, 397, 398, 399, 400, 401, 402, 403, 404, 405, 406, 407, 408, 409, 410, 411, 412, 413, 442, 444, 446, 448, 460, 461, 462, 463, 465, 611, 616, 618, 624, 625, 637, 674, 743, 816 and 824.**

### Feature **Group** **9**: Majority subtypes

Subtype information was included for every subtype found in at least ten sequences. The subtypes included in this analysis were: CRF01_AE, CRF02_AG, CRF07_BC, A1, B, C, D, (group) O, and the recombinants A1C and A1D. All other minority subtypes were grouped into a category described as “Other”. Subtypes were represented as a single categorical variable, and as binary indicator variables, one for each subtype (including “Other”).

### Feature **Group** **10**: Region-specific counts of PNGS

To account for the possibility that an enrichment of PNGSes in a specific region could influence VRC01 neutralization or sensitivity, we included variables that indicate the total number of PNGSes in key regions of the HIV-1 envelope. To determine whether a given site is a PNGS, the standard, three-position N-linked glycosylation motif [N][!P][S|T] was used [17]. The total number of PNGSes for the following site sets were included in the analysis: all positions in Env, all positions in gp120, all positions in the VRC01 footprint, all CD4 binding sites, all positions in the V5 region, all positions in Loop D, all positions in Loop E, the 8 sites (197, 276, 362, 363, 386, 392, 462, and 463) identified in [18-20] as constituting a glycan shield to VRC01 binding, the five sites (61, 64, 197, 276, and 386) identified by [18] as sites where VRC01 interacts with the HIV-1 trimer, and the 91 amino acid sites identified in Feature **Groups** **1** to **7**.

### Feature **Group** **11**: Viral geometry

The total lengths (excluding gaps and frameshifts) of the following regions within the Env sequence were used: the entire Env polyprotein, the entire gp120 protein, the V5 region, Loop D, and Loop E.

The last three variables listed above (V5, Loop D and Loop E) are also present as binary indicator variables for whether they are outliers from the main body of the distribution of values. For V5, the main body of the distribution is a length of 9 or 10 AA. For Loop D, the main body of the distribution is a length of 9 AA. For Loop E, the main body of the distribution is a length of 2 or 3 AA. Any lengths of these regions outside of these values (above or below) are regarded as outliers. These variable names are the same as those above, except for the addition of the outliers indicator, resulting in three new binary indicator variables.

### Feature **Group** **12**: Cysteine counts

Also included in the analysis was the total number of cysteines present within the following regions of the Env sequence: the entire Env polyprotein, the entire gp120 protein, the V5 region, Loop D, and Loop E.

### Feature **Group** **13**: Steric bulk at critical locations

As described in Methods, three variables were included to evaluate the impact of steric bulk of residues in critical locations of the HIV-1 envelope. One variable was included for each of the V5, Loop D and Loop E regions. For each of these regions, the variable included in the analysis was the sum of the residues in the respective region that was defined as “small” by Taylor [21].

### Creation of new TZM-bl neutralization resistance outcome variables from the CATNAP data (infectivity results)

The neutralization studies yielded the readouts of IC_50_ and/or IC_80_ (in units of μg/ml). IC_50_ was recorded for all of the VRC01 studies. IC_80_ was captured most (79.6%) of the time. Neutralization studies compared one (or many) pseudoviruses with one (or many) antibodies, and some antibody-pseudovirus combinations were studied more than once, typically by different groups. The following process was performed to reconcile these different results into an appropriate form to be used in the analysis here:

### Single vs. multiple studies of VRC01-mediated neutralization of a given HIV-Env pseudovirus

HIV-Env pseudoviruses for which VRC01-mediated neutralization was assessed with a single study were relatively simple to handle, as their IC_50_/IC_80_ values come from that single study. For HIV-Env pseudoviruses for which VRC01-mediated neutralization was assessed in multiple studies, however, CATNAP summarizes their IC_50_/IC_80_ values with the geometric mean. We continued the use of this value out of consistency.

### Handling right-censored values

Occasionally, the VRC01 IC_50_ and IC_80_ values retrieved from CATNAP were right-censored (e.g., “UD:>50”). In these cases it should be considered that neutralization was not detected. Each right-censored VRC01 IC_50_ or IC_80_ value was imputed with the value of twice the upper limit of detection (e.g. the value “UD:>10” was imputed as “20”). For those pseudoviruses which had neutralization results from multiple studies, we took the arithmetic mean of the natural log of all available neutralization values, both observed and (when necessary) imputed. This results in a single composite neutralization value for each pseudovirus, for the purpose of predicting the continuous neutralization outcomes.

Thirteen pseudoviruses were excluded from the analysis, because their single IC50 measurement was recorded as right-censored at 1 μg/ml. According to the study that produced these results [22], this unusually low limit of censorship was due to a lack of reagent. As such, we excluded these pseudoviruses from the study, as their neutralization results were regarded as unreliable.

## Supplementary References

1. Yoon H, Macke J, West AP, Jr., Foley B, Bjorkman PJ, Korber B, et al. CATNAP: a tool to compile, analyze and tally neutralizing antibody panels. Nucleic Acids Res. 2015;43(W1):W213-9.

2. Zhou T, Georgiev I, Wu X, Yang ZY, Dai K, Finzi A, et al. Structural basis for broad and potent neutralization of HIV-1 by antibody VRC01. Science. 2010;329(5993):811-7.

3. Kabsch W, Sander C. Dictionary of protein secondary structure: pattern recognition of hydrogen-bonded and geometrical features. Biopolymers. 1983;22(12):2577-637.

4. Joosten RP, te Beek TA, Krieger E, Hekkelman ML, Hooft RW, Schneider R, et al. A series of PDB related databases for everyday needs. Nucleic Acids Res. 2011;39(Database issue):D411-9.

5. Gaschen B, Kuiken C, Korber B, Foley B. Retrieval and on-the-fly alignment of sequence fragments from the HIV database. Bioinformatics. 2001;17(5):415-8.

6. Yu WH, Zhao P, Draghi M, Arevalo C, Karsten CB, Suscovich TJ, et al. Exploiting Glycan Topography for Computational Design of Env Glycoprotein Antigenicity. PLoS Comput Biol (accepted; in press). 2018.

7. Blish CA, Nguyen MA, Overbaugh J. Enhancing exposure of HIV-1 neutralization epitopes through mutations in gp41. PLoS Med. 2008;5(1):e9. Epub 2008/01/08.

8. O'Rourke SM, Schweighardt B, Phung P, Mesa KA, Vollrath AL, Tatsuno GP, et al. Sequences in glycoprotein gp41, the CD4 binding site, and the V2 domain regulate sensitivity and resistance of HIV-1 to broadly neutralizing antibodies. J Virol. 2012;86(22):12105-14. Epub 2012/08/31.

9. O'Rourke SM, Schweighardt B, Scott WG, Wrin T, Fonseca DP, Sinangil F, et al. Novel ring structure in the gp41 trimer of human immunodeficiency virus type 1 that modulates sensitivity and resistance to broadly neutralizing antibodies. J Virol. 2009;83(15):7728-38. Epub 2009/05/29.

10. Bradley T, Trama A, Tumba N, Gray E, Lu X, Madani N, et al. Amino Acid Changes in the HIV-1 gp41 Membrane Proximal Region Control Virus Neutralization Sensitivity. EBioMedicine. 2016;12:196-207. Epub 2016/10/26.

11. Park EJ, Gorny MK, Zolla-Pazner S, Quinnan GV, Jr. A global neutralization resistance phenotype of human immunodeficiency virus type 1 is determined by distinct mechanisms mediating enhanced infectivity and conformational change of the envelope complex. J Virol. 2000;74(9):4183-91. Epub 2001/02/07.

12. Ringe R, Bhattacharya J. Association of enhanced HIV-1 neutralization by a single Y681H substitution in gp41 with increased gp120-CD4 interaction and macrophage infectivity. PLoS One. 2012;7(5):e37157. Epub 2012/05/19.

13. Thali M, Charles M, Furman C, Cavacini L, Posner M, Robinson J, et al. Resistance to neutralization by broadly reactive antibodies to the human immunodeficiency virus type 1 gp120 glycoprotein conferred by a gp41 amino acid change. J Virol. 1994;68(2):674-80. Epub 1994/02/01.

14. Wilson C, Reitz MS, Jr., Aldrich K, Klasse PJ, Blomberg J, Gallo RC, et al. The site of an immune-selected point mutation in the transmembrane protein of human immunodeficiency virus type 1 does not constitute the neutralization epitope. J Virol. 1990;64(7):3240-8. Epub 1990/07/01.

15. Klasse PJ, McKeating JA, Schutten M, Reitz MS, Jr., Robert-Guroff M. An immune-selected point mutation in the transmembrane protein of human immunodeficiency virus type 1 (HXB2-Env:Ala 582(-->Thr)) decreases viral neutralization by monoclonal antibodies to the CD4-binding site. Virology. 1993;196(1):332-7. Epub 1993/09/01.

16. Back NK, Smit L, Schutten M, Nara PL, Tersmette M, Goudsmit J. Mutations in human immunodeficiency virus type 1 gp41 affect sensitivity to neutralization by gp120 antibodies. J Virol. 1993;67(11):6897-902. Epub 1993/11/01.

17. Schwarz F, Aebi M. Mechanisms and principles of N-linked protein glycosylation. Curr Opin Struct Biol. 2011;21(5):576-82.

18. Stewart-Jones GB, Soto C, Lemmin T, Chuang GY, Druz A, Kong R, et al. Trimeric HIV-1-Env Structures Define Glycan Shields from Clades A, B, and G. Cell. 2016;165(4):813-26.

19. Crooks ET, Tong T, Chakrabarti B, Narayan K, Georgiev IS, Menis S, et al. Vaccine-Elicited Tier 2 HIV-1 Neutralizing Antibodies Bind to Quaternary Epitopes Involving Glycan-Deficient Patches Proximal to the CD4 Binding Site. PLoS Pathog. 2015;11(5):e1004932.

20. Crooks ET, Osawa K, Tong T, Grimley SL, Dai YD, Whalen RG, et al. Effects of partially dismantling the CD4 binding site glycan fence of HIV-1 Envelope glycoprotein trimers on neutralizing antibody induction. Virology. 2017;505:193-209.

21. Taylor WR. The Classification of Amino-Acid Conservation. J Theor Biol. 1986;119(2):205-&.

22. Goo L, Jalalian-Lechak Z, Richardson BA, Overbaugh J. A combination of broadly neutralizing HIV-1 monoclonal antibodies targeting distinct epitopes effectively neutralizes variants found in early infection. J Virol. 2012;86(19):10857-61. Epub 2012/07/28.
